# Supplementary material for: Using real-world evidence to evaluate the long-term health and economic impact of the digital tool Grohealth W8Buddy supporting access to specialist weight management services: a protocol for a cohort observational study
Source: BMJ Open. 2026 Jan 21;16(1):e109111. doi: 10.1136/bmjopen-2025-109111 (PMC12853449; doi:10.1136/bmjopen-2025-109111)
Supplement: Supplementary data [file bmjopen-16-1-s005.pdf]

Supplementary table 2. Data management

Information	Description
Data collection tools and source document identification	Study data will be collected on the screening logs, W8Buddy App and validated questionnaires (see Table 1) electronically. Health record data for each site will be accessed by local research team. An online validated, GCP compliant, Electronic Data Capture system will be used to record and store study data, this will be the main Study Database. Individual user log-in access to this database will be granted to only those in the study team that require it for the performance of their role. The W8Buddy App will collect data for participants in the digital arm of the study, then transfer this data in a secure way to the main study database. Only research team and members of the DDM team will have access to the main study database.<br>Source data for standard care procedures will be from hospital medical records. Other outcome measures not collected routinely will either be collected directly from the patient, their clinical care team, or App collaborators and entered directly on to the App/Database.<br>Participants will be able to complete the outcome questionnaires by phone, email or online if they prefer. If done online, they will be emailed a secure, unique link to complete the questionnaires, where their answers will directly input onto the database.<br>The research team at UHCW (see page 1 for details) will maintain oversight of the Study Database during the study to ensure that any missing data is supplied in a timely manner.<br><br>Semi-structured interviews will be audio recorded using teleconferencing software (e.g., Zoom) and transcribed by an external transcription company. The data will be stored on secure University of Warwick computers and accessed only by research team located at Warwick.<br><br>Data entered into the app will be stored in secure Azure cloud.<br>DDM's Data Protection and Information Governance Policy details how we comply to and exceed UK GDPR requirements through:<br>• Consent is requested on referral (website or telephone), paper-based for face-to-face and digitally before using the app. Our privacy policy is available on our website, physically at face-to-face sessions and on in-app/platform. On signup (and signposted in-app), users are informed on how their data will be used, who will access it, their rights and consent/opt-in to

	sharing anonymised data (including with Commissioner and OHID). The app locks users out after several unsuccessful access attempts and logs users out after periods of inactivity. All user access is logged (IP, time, date, browser details) in case of safeguarding or data issues.<br>• Individuals have the right to access their personal data, rectify incorrect data, erase their data and restrict or object to its processing. This is specified in our privacy policy.<br>• We are aware that if we have data breaches, we report within 72 hours of becoming aware of the breach to the Information Commissioners' Office and University's Legal & Compliance Services, in accordance with the University's data protection policies and the ICO as per DDM policies. DDM has a Full Business Continuity Plan which includes a Disaster Recovery Plan tested quarterly.<br>• Service-users' personal data is never transferred outside of the UK. DDM's data servers are hosted by Microsoft Azure in the UK and are fully encrypted-at-rest and in-transit using AES256 cryptography.<br>• Staff are provided an individual account with restricted privileges, dependent on their tasks/role. Any data which is invalid, irrelevant or no longer required (including user removal requests) is securely deleted.<br>• All staff undergo mandatory data/information governance training bi-annually and perform a self-assessment (e.g. passwords, UK GDPR) to identify training needs to provide further formal training.<br>• All information collected, stored and communicated (internally/externally) is carried out in-line with the Data Protection Policy through use of secure digital patient records, encrypted email and private communication software.<br>• All data transfer is done via FTPS (Secure FTP) or encrypted data transferral method.<br>• Paper data and local servers are stored in a locked, CCTV-protected room in DDM Headquarters. All data on the servers is password protected and encrypted using 256-AES (BitLocker on Windows).<br>• One individual via USB/over FTPS takes backups daily. Backups are stored in-the-cloud on a UK-based 256-AES encrypted hard-drive. Paper data is stored in a locked room. Only the DPO has the login details and keys to the data room. All passwords used for data handling

	are 28 characters long and changed weekly. Access is only granted to authorised staff for limited purposes and is time restricted.
Data handling and record keeping	Screening and recruitment logs of all patients approached to take part, and participants enrolled in the study will be held at each site. Upon confirmation of eligibility, participants will be assigned a unique study ID via the study website, which will be used to identify all documents associated with that participant for the duration of the study.<br>It is the responsibility of the PI at each site to ensure that the data that has been submitted is verified and accurate in reflecting the clinical outcome of the participants.<br>This is electronic-based study, with no paper-based collection. Data will be stored in on secure cloud-based databases including the W8Buddy App, with restricted access and regular back-ups in the cloud-based server.<br>Personal data collected during the study will be handled and stored in accordance with the UK GDPR. Handling of personal data will be clearly documented in the participant information sheet and consent obtained.<br>Following all query resolution, the database will be exported for statistical analysis. These procedures along with data entry instructions will be detailed in a Data Management Plan (DMP) produced by the study coordinator and statistician.
Access to data	Direct access will be granted to authorised representatives from the Sponsor, host institution and the regulatory authorities to permit study-related monitoring, audits and inspections - in line with participant consent.<br>Delegated research team members will be responsible for entering data into the database. This may include the site Principal Investigator (PI), research nurses, and study team members based at UHCW, UHB, LTT, and HDUHBT. Access to the data will be restricted to the central study team only. Additionally, all staff granted access to the database will be listed in the delegation log and must hold Good Clinical Practice (GCP) certification.
Archiving	Following the resolution of queries and confirmation of study close-out by the Chief Investigator, all essential documentation will be transferred to a third-party archiving service, which provides suitable fire- and water-resistant facilities. Study files will be archived for a period of 10 years.

	Access to the study documentation will be restricted to named individuals within the study team with express permission from the Chief Investigator.

Supplementary table 3. Safety reporting

Information	Description
Adverse event	An AE is any untoward medical occurrence experienced by a patient while participating in the trial. This includes occurrences which are not necessarily caused by or related to the trial intervention.
Serious adverse event	A SAE is characterised as an untoward occurrence deemed to be related to the intervention under investigation that:<br>1. Results in death within 30 days of the intervention<br>2. Is life-threatening<br>3. Requires hospitalisation or an extension of an existing inpatients' hospitalisation<br>4. Results in persistent or significant disability or incapacity<br>5. Consists of a congenital anomaly or birth defect<br>6. Otherwise considered medically significant by the investigator
Reporting	• The Chief Investigator (or designated representative) will assess all reported SAEs for causality and expectedness.<br>• All intervention related AEs must be documented using the relevant electronic Case Report Form (eCRF), patients will report these to the research team who will then enter this data in the eCRF.<br>• SAEs should be reported for any occurrence from the time informed consent is obtained until study completion.<br>All participants will have contact details to the research team and will be able to inform the research team of any AEs/SAEs occurring between study visits. Participants will be asked to

	contact the research nurse as soon as possible with any potential adverse events. The research nurse will call the patient to discuss the adverse event; this will be escalated as per each standard hospital protocols and will be escalated to each site PI for investigation and recording as required<br>All SAEs must be reported to the study team within 24 hours of the Principal Investigator (or designated representative) becoming aware of the event. This should be done by completing the SAE eCRF and emailing:<br>Email address: W8Buddystudyofficeinbox@uhcw.nhs.uk
Follow up	SAEs must be followed until clinical recovery is complete or the condition stabilises. Outcomes of SAEs should be reported to the study team promptly using the relevant eCRF/CRF. These should be sent to the W8Buddy Study inbox (W8Buddystudyofficeinbox@uhcw.nhs.uk) as soon as the Principal Investigator (or designated representative) is aware of the outcome.<br><br>**EVENTS THAT DO NOT REQUIRE REPORTING AS AN ADVERSE EVENT OR SERIOUS ADVERSE EVENT**<br>This trial recruits' participants with pre-existing long-term conditions (e.g., type 2 diabetes, hypertension), likely requiring medical attention during the trial. Given:<br>• The CE marking of the W8Buddy intervention<br>• Safety not being an outcome of interest<br>A risk-based approach to reporting will apply, in which only AEs deemed related to the W8Buddy intervention will be reported.<br>In addition, the following events will not require reporting as SAEs:<br>• Scheduled hospital visits (e.g., routine outpatient appointments, elective surgeries).<br>• Visits related to pre-existing medical conditions or their progression.<br>• Foreseeable or predictable SAEs attributed to terminal illness or similar conditions.<br>• SAEs related to standard care medications

Supplementary table 4. Study oversight

Information	Description
Roles and responsibilities of the sponsor	The University of Warwick will act as study sponsor. The study will be conducted in accordance with Sponsor's Standard Operating procedures. The University of Warwick has a specialist insurance policy in place which would operate in the event of any participant suffering harm as a result of their involvement in the study Zurich Municipal Insurance. NHS indemnity operates in respect of the clinical treatment that is provided.
Roles and responsibilities of the funder	This study is funded by the National Institute for Health and Care Research Invention for Innovation (NIHR i4i) programme (NIHR208100).
Study manage/co-ordinator	The Trial Co-ordinator / Manager will have responsibility for overseeing day to day coordination of the trial and reporting regularly to the TSC. The Study Manager's responsibilities include, but are not limited to:<br>• Coordinating protocol development, patient and trial management documents<br>• Correspondence with study funder and tracking of progress against agreed milestones<br>• Setting up and maintaining the Trial Master File<br>• Ensuring necessary approvals are in place before the start of the trial at each site<br>• Providing training to trial personnel<br>• Providing data management support; including data input, maintenance of the trial database and raising of queries<br>• Producing trial progress reports and coordinating TSC meetings and minutes<br>• Ensuring data security and quality and ensuring data protection laws are adhered to<br>• Ensuring complete records are in place for audit and monitoring purposes<br>• Ensuring the trial is conducted in accordance with the ICH GCP<br>• Archiving all original trial documents including the data forms in line with UHCW NHS Trust policy.

Site principal investigators	Site Principal Investigator responsibilities include, but are not limited to:<br>• Ensuring that the trial is conducted as set out in the protocol and supporting documents<br>• Delegating trial related responsibilities only to suitably trained and qualified personnel and ensuring that those with delegated responsibilities fully understand and agree to the duties being delegated to them<br>• Ensuring that CVs and evidence of appropriate training for all Site staff are available in the Trial Site File<br>• Ensuring that all delegated duties are captured in the study Delegation Log<br>• Ensuring all Adverse Events are documented and reported promptly to the Trial Manager<br>• Accountability for trial treatments at their site<br>• Ensuring the trial is conducted in accordance with ICH GCP principles.<br>• Allowing access to source data for monitoring, audit and inspection<br>• Ensuring that all source data is complete and provided to the Trial Manager at regular intervals.
Monitoring, audit and inspection	The study will be monitored by the study management group to ensure that the study is being conducted as per protocol, adhering to Research Governance and GCP. Central monitoring activities will be performed such as data quality checks. The approach to, and extent of, monitoring will be specified in a study monitoring plan determined by the risk assessment undertaken prior to the start of the study.<br><br>The recruiting site is obliged to assist the sponsor in monitoring the study. These may include hosting site visits, providing information for remote monitoring, or putting procedures in place to monitor the study internally.<br><br>The study may be monitored by the Warwick University representative of the Sponsor, to ensure that the study is being conducted as per protocol, adhering to Research Governance and GCP. The approach to, and extent of, monitoring will be specified in a study monitoring plan determined by the risk assessment undertaken prior to the start of the study.
