# Supplementary material for: Using real-world evidence to evaluate the long-term health and economic impact of the digital tool Grohealth W8Buddy supporting access to specialist weight management services: a protocol for a cohort observational study
Source: BMJ Open. 2026 Jan 21;16(1):e109111. doi: 10.1136/bmjopen-2025-109111 (PMC12853449; doi:10.1136/bmjopen-2025-109111)
Supplement: Supplementary data [file bmjopen-16-1-s001.pdf]

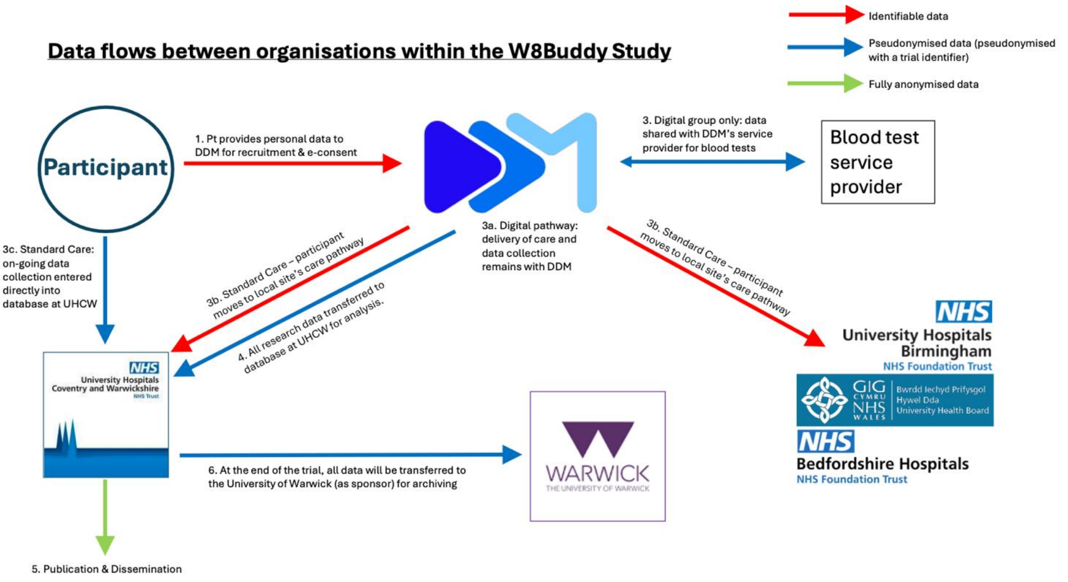

Order of data flows in W8Buddy Study

1. Participant provides personal information to DDM to facilitate online recruitment and e-consent.
2. After giving informed consent, participant chooses pathway:
   a) Digital – participant continues to provide data to DDM for delivery of care and data collection
   b) Standard Care – participant moves to local site's care pathway
   c) Standard Care – on-going data collection for study is entered directly into database at UHCW
3. Digital group only: data shared with DDM's service provider for blood tests.
4. All research data transferred to database at UHCW for analysis.
5. Following completion of the study, the findings will be published and disseminated using fully anonymised data.
6. At the end of the trial, all data will be transferred to the University of Warwick (as sponsor) for archiving
