# Supplementary material for: Using real-world evidence to evaluate the long-term health and economic impact of the digital tool Grohealth W8Buddy supporting access to specialist weight management services: a protocol for a cohort observational study
Source: BMJ Open. 2026 Jan 21;16(1):e109111. doi: 10.1136/bmjopen-2025-109111 (PMC12853449; doi:10.1136/bmjopen-2025-109111)
Supplement: Supplementary data [file bmjopen-16-1-s004.pdf]

Supplementary Table 1

Data collected	When and how data collected from digital (D)/ Standard care (SC) group	Data used for assessment of:
**Weight, height, and BMI**	Month 0,6,12,18,24.<br><br>D: app, dashboard<br><br>SC: survey, clinical notes	Weight loss
**Metabolic markers (HBA1c, lipids)**	Month 0,6,18.<br><br>D: app, dashboard (the same standard of care blood tests will be done remotely instead using clinical phlebotomy partners and samples will be returned for analysis, then discarded as per usual clinical procedures.<br><br>SC: survey, clinical notes (only routine blood test results will be collected in the standard of care pathway, as per usual standard of care for NHS hospitals)	Metabolic impact
**Blood pressure**	Month 0,6,18.<br><br>D: app, dashboard<br><br>SC: survey, clinical notes	Cardiovascular marker

**Waist circumference**	Month 0,6,12,18,24.<br><br>D: app, dashboard<br><br>SC: survey, clinical notes	Body composition changes
**Health related quality of life (EQ-5D-5L)**	Month 0,6,12,18,24.<br><br>D: app<br><br>SC: survey	Cost-effectiveness and quality of life
**PHQ8, satisfaction with life scale, Karolinska sleepiness scale, GAD7, BEDS-7**	Month 0,6,12,18,24.<br><br>D: app<br><br>SC: survey	Biopsychosocial outcomes
**Clinical endpoints (stroke, myocardial infarction)**	Month 12, 24, 36, 48, 60<br><br>D: app, dashboard<br><br>SC: clinical notes	Major adverse cardiovascular events
**Distance to local NHS facility for participants**	Month 0.<br><br>Research team review of GP notes for D and SC	Health economic calculations

**Time needed to travel to local NHS facility for participants**	Month 0.<br><br>Research team review of GP notes for D and SC, plus<br><br>D: app<br><br>SC: survey	Health economic calculations
**Date when participants dropped out of study if applicable**	As applicable.<br><br>D: app<br><br>SC: survey	Accurate tracking
**Reason why participant on standard care dropped out of study if applicable**	SC: Contact with research nurse	Why participants drop out of standard care
**Number of consented participants**<br><br>• **choosing the digital pathway**<br><br>• **starting on the pathway**<br><br>• **dropping out**	Month 0-24.<br><br>Reviews of dashboard and research database	Willingness and engagement level of digital pathway<br><br>Sampling frame for participant interviews
**Participant baseline characteristics: comorbidities, sex, age, ethnicity, disabilities, geographical region, socioeconomic status**	Month 0.<br><br>Research team review of GP notes for D and	Stratification of interview sample, sensitivity analyses, predictors of cost-effectiveness and engagement with digital pathway

	SC, plus<br><br>D: app<br><br>SC: survey	
**Comorbidities at follow up (hypertension, obstructive sleep apnoea, osteoarthritis, non-alcoholic fatty liver disease, polycystic ovarian syndrome, type 2 diabetes, dyslipidaemia)**	Month 6, 12, 18, 24<br><br>D: app, review of health records<br><br>SC: survey, review of health records	Assessment of diseases associated with obesity
**Type of concomitant and weight-management medication, dose, and prescription dates, whether NHS or non-NHS prescribed**	Month 0,6,12,18,24<br><br>D: app<br><br>SC: survey, clinical notes	Calculation of cost<br><br>Medication adherence
**Reason for stopping the medicine, including side effects (including physical and psychological effects as per STEP 5 trial. These include nausea, diarrhoea, constipation, vomiting, abdominal pain, dyspepsia, gastroenteritis, decreased appetite, headache, eructation, nasopharyngitis, headache, back pain, upper respiratory tract infection, gallbladder**	Month 0,6,12,18,24<br><br>D: app, dashboard<br><br>SC: survey, clinical notes	Cost-effectiveness, Biomedical explanation of adherence rates

**related disorders, cholelithiasis, pancreatitis, cardiovascular disorders, injection site reactions, hypoglycaemia, psychiatric disorders , reaching predefined weight loss goals, lack of adherence to prescribed medicine**		
**Use of and response to digital pathway**	Month 0-24<br><br>D: app<br><br>SC: appointment records and clinical notes<br><br>Information collected on all patients who switch	Cost-effectiveness,<br><br>Adherence to digital pathway; variation in adherence by patient characteristics<br><br>User experience and process evaluation

	from SC to D and vice versa, even though this is expected to be low	
**Adverse events, including only intervention-related adverse effects**	Month 0-24<br><br>D: consultation with clinicians on dashboard, medication tracking, captured at real-time<br><br>SC: clinical notes, surveys at month 6,12,18,24	Cost-effectiveness, understanding the experience of users, creating blueprint for safe incorporation of digital pathway
**Healthcare resource use: Data from providers about the multidisciplinary service, resource use (e.g. rates of referral to bariatric surgery)**<br><br>**Data from participants on number of GP visits, unplanned admissions to A&E**	Month 0,6,12,18,24<br><br>D: app and reports from digital provider<br><br>SC: survey, clinical notes	Cost-effectiveness
**Cost associated with implementing and maintaining digital pathway**	D: Digital provider	Cost-effectiveness

**Cost estimates for specialist WMS**	Month 0,6,12,18,24<br><br>SC: clinical records, survey	Cost-effectiveness
**Implementation, adaptations, and contextual influences**	Observation of pathway delivery team meetings, meeting notes, semi-structured interviews with implementers and clinical staff delivering intervention.<br><br>During initial implementation (month 0) and in months 12 & 18	Implementation process to inform formative feedback<br><br>Manual for future NHS implementation and process evaluation
**Availability of digital pathway and medicine to participant including**<br><br>**staff training and quality assurance**	D: app and related organisational records<br><br>SC: clinical records and related organisational records throughout delivery	Fidelity of delivery of intervention<br><br>Experience of delivery of intervention<br><br>Process evaluation
**Brief structured interview with study participants not choosing digital pathway**	Within approx. 1 week of choice<br><br>Remote interview	Why participants do not choose digital pathway (process evaluation)
**Brief structured interview with study participants not adherent to digital pathway and/or medicine**	Sample for participants demonstrating early/late non-adherence or on/off adherence<br><br>Remote interview	Why participants drop out/return to digital pathway/medicine process evaluation)
**Semi-structured interviews with study participants using digital pathway**	Sample stratified by gender and progress	Understand how user engages with digital pathway, why, perceived impacts and user

BMJ Publishing Group Limited (BMJ) disclaims all liability and responsibility arising from any reliance placed on this supplemental material which has been supplied by the author(s)

	through pathway (6, 12, 18 months)<br><br>Remote interview	understanding of mechanism of impact (process evaluation).

Abbreviations: BMI, Body mass index; **EQ-5D-5L**, EuroQol five dimensions and levels; GAD, general anxiety disorder scale; PHQ-8, Patient Health Questionnaire 8, BEDS-7, Binge eating disorder screener
