# Supplementary figures and images for: Using real-world evidence to evaluate the long-term health and economic impact of the digital tool Grohealth W8Buddy supporting access to specialist weight management services: a protocol for a cohort observational study
Source: BMJ Open. 2026 Jan 21;16(1):e109111. doi: 10.1136/bmjopen-2025-109111 (PMC12853449; doi:10.1136/bmjopen-2025-109111)

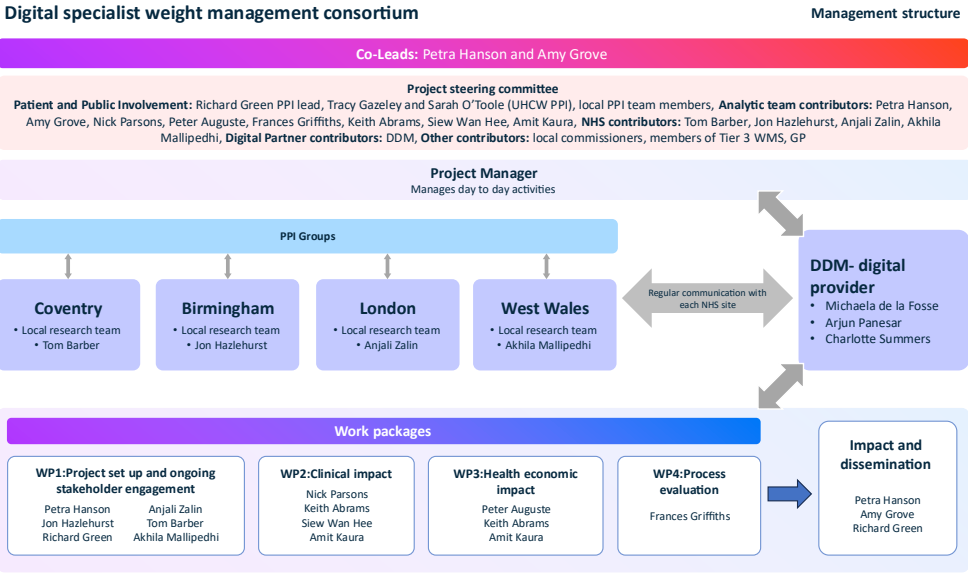

Supplement: Supplementary data [file bmjopen-16-1-s002.pdf]
