# Supplementary material for: Using real-world evidence to evaluate the long-term health and economic impact of the digital tool Grohealth W8Buddy supporting access to specialist weight management services: a protocol for a cohort observational study
Source: BMJ Open. 2026 Jan 21;16(1):e109111. doi: 10.1136/bmjopen-2025-109111 (PMC12853449; doi:10.1136/bmjopen-2025-109111)
Supplement: Supplementary data [file bmjopen-16-1-s003.pdf]

Ymchwil Iechyd a Gofal Cymru
Health and Care Research Wales

NHS Health Research Authority

Dr Petra Hanson
Clinical Lecturer
University of Warwick
Warwick Medical School, University of Warwick,
Coventry
CV4 7AL

Email: approvals@hra.nhs.uk
HCRW.approvals@wales.nhs.uk

29 July 2025

Dear Dr Hanson

HRA and Health and Care Research Wales (HCRW) Approval Letter

**Study title:**	**Long-term health and economic impact of using digital tool Gro Health W8Buddy to support people accessing specialist weight management services**
**IRAS project ID:**	**355370**
**Protocol number:**	**1.0**
**REC reference:**	**25/EM/0147**
**Sponsor**	**Warwick University**

I am pleased to confirm that **HRA and Health and Care Research Wales (HCRW) Approval** has been given for the above referenced study, on the basis described in the application form, protocol, supporting documentation and any clarifications received. You should not expect to receive anything further relating to this application.

Please now work with participating NHS organisations to confirm capacity and capability, in line with the instructions provided in the “Information to support study set up” section towards the end of this letter.

How should I work with participating NHS/HSC organisations in Northern Ireland and Scotland?
HRA and HCRW Approval does not apply to NHS/HSC organisations within Northern Ireland and Scotland.

If you indicated in your IRAS form that you do have participating organisations in either of these devolved administrations, the final document set and the study wide governance report (including this letter) have been sent to the coordinating centre of each participating nation. The relevant national coordinating function/s will contact you as appropriate.

Please see IRAS Help for information on working with NHS/HSC organisations in Northern Ireland and Scotland.

How should I work with participating non-NHS organisations?
HRA and HCRW Approval does not apply to non-NHS organisations. You should work with your non-NHS organisations to obtain local agreement in accordance with their procedures.

What are my notification responsibilities during the study?

The standard conditions document "*After Ethical Review – guidance for sponsors and investigators*", issued with your REC favourable opinion, gives detailed guidance on reporting expectations for studies, including:

- Registration of research
- Notifying amendments
- Notifying the end of the study

The HRA website also provides guidance on these topics, and is updated in the light of changes in reporting expectations or procedures.

Who should I contact for further information?
Please do not hesitate to contact me for assistance with this application. My contact details are below.

Your IRAS project ID is **355370**. Please quote this on all correspondence.

Yours sincerely,
Chris Kitchen

Email: approvals@hra.nhs.uk

Copy to: *Mrs Carole Harris*

List of Documents

The final document set assessed and approved by HRA and HCRW Approval is listed below.

Document	Version	Date
Contract/Study Agreement template [W8Buddy IRAS 355370 mNCA_July_2022]	v2.3	
Evidence of Sponsor insurance or indemnity (non NHS Sponsors only) [UoW Insurance]	N/A	02 July 2024
GP/consultant information sheets or letters [W8Buddy_GPLetter_v1.0_ 06Jun2025]	v1.0	08 May 2025
Interview schedules or topic guides for participants [W8Buddy_ Topic guides for interviews_v1.0_06Jun2025]	v1.0	06 June 2025
Interview schedules or topic guides for participants [W8Buddy_ Topic guides for HCP interviews_v1.0_12 Jun2025]	1.0	12 June 2025
IRAS Application Form [IRAS_Form_10062025]	N/A	10 June 2025
Letter from funder [Outcome Letter NIHR208100]	N/A	03 September 2024
Letter from sponsor [SOC.11 24-25 W8Buddy Sponsorship Approval Letter]	N/A	06 June 2025
Non-validated questionnaire [W8Buddy_CRSI_v1.0_06Jun2025]	1.0	06 June 2025
Organisation Information Document [Organisation_Information_Document_NonCommercial_v1-8_April_2024+W8Buddy(2)]		01 June 2025
Other [W8Buddy_Websiteconsentprocess_v1.0_06Jun2025]	v1.0	06 June 2025
Other [W8Buddy_Storyboard_v1.0_13Jun2025]	vX.0	13 June 2025
Other [W8Buddy Website Screenshots_v1.0_13Jun2025]	v1.0	13 June 2025
Other [Ethics Response 11Jul2025]	N/A	11 July 2025
Participant consent form [W8Buddy_ConsentForm_HCP_v2.0_ 11Jul2025]	2.0	11 July 2025
Participant consent form [W8Buddy_ConsentForm_v1.0_06Jun2025]	v1.0	06 June 2025
Participant information sheet (PIS) [W8Buddy_CondensedPIS_v1.0_11Jul2025]	v1.0	11 July 2025
Participant information sheet (PIS) [W8Buddy_PIS_HCP_v2.0_11Jul2025]	v2.0	11 July 2025
Participant information sheet (PIS) [W8Buddy_PIS_v2.0_11Jul2025]	v2.0	11 July 2025
Research protocol or project proposal [W8Buddy Protocol_ v1.0_06Jun2025]	v1.0	06 June 2025
Schedule of Events or SoECAT [Funder Export_342299_SoECAT_Gro Health W8Buddy in Specialist Weight Management Services]		
Summary CV for Chief Investigator (CI) [PH CV june 2025]		06 June 2025
Validated questionnaire [PHQ-8_English-UK]		
Validated questionnaire [GAD7_English for the UK]		
Validated questionnaire [Effective_UK (English) EQ-5D-5L Paper Self-Complete v1.2]	v1.2	
Validated questionnaire [BEDS-7_AU1.0_eng-USori]		
Validated questionnaire [Satisfaction With Life Scale]		
Validated questionnaire [Karolinska Sleepiness Scale]		

IRAS project ID	355370

Information to support study set up

The below provides all parties with information to support the arranging and confirming of capacity and capability with participating NHS organisations in England and Wales. This is intended to be an accurate reflection of the study at the time of issue of this letter.

Types of participating NHS organisation	Expectations related to confirmation of capacity and capability	Agreement to be used	Funding arrangements	Oversight expectations	HR Good Practice Resource Pack expectations
Research activities and procedures as per the protocol and other study documents will take place at participating NHS organisations.	Research activities should not commence at participating NHS organisations in England or Wales prior to their formal confirmation of capacity and capability to deliver the study in accordance with the contracting expectations detailed.	An Organisation Information Document has been submitted but the sponsor is intending to use a separate agreement. The sponsor has supplied the unmodified model agreement and intends to use this with participating NHS organisations.	Study funding arrangements are detailed in the Organisation Information Document.	A Principal Investigator should be appointed at participating NHS organisations.	Where an external individual who does not already hold an NHS employment contract will be conducting any of the research activities that will be undertaken at this site type then they would be expected to hold an Honorary Research Contract. This should be issued be on the basis of a Research Passport (if university employed) or an NHS to NHS confirmation of pre-engagement checks letter (if NHS employed). These should confirm Occupational Health Clearance. These should confirm enhanced DBS checks and appropriate barred list checks.<br>External staff holding pre-existing NHS employment contracts, or staff only

					undertaking survey or interview activities should obtain a Letter of Access. This should be issued be on the basis of a Research Passport (if university employed) or an NHS to NHS confirmation of pre-engagement checks letter (if NHS employed). These should confirm Occupational Health Clearance if there is direct contact with participants These should confirm standard DBS checks.

Other information to aid study set-up and delivery

This details any other information that may be helpful to sponsors and participating NHS organisations in England and Wales in study set-up.
The applicant has indicated that they intend to apply for inclusion on the NIHR CRN Portfolio.
