# Supplementary material for: Using real-world evidence to evaluate the long-term health and economic impact of the digital tool Grohealth W8Buddy supporting access to specialist weight management services: a protocol for a cohort observational study
Source: BMJ Open. 2026 Jan 21;16(1):e109111. doi: 10.1136/bmjopen-2025-109111 (PMC12853449; doi:10.1136/bmjopen-2025-109111)
Supplement: Author's manuscript [file bmjopen-2025-109111.draft_revisions.pdf]

BMJ Open

BMJ Open is committed to open peer review. As part of this commitment we make the peer review history of every article we publish publicly available.

When an article is published we post the peer reviewers' comments and the authors' responses online. We also post the versions of the paper that were used during peer review. These are the versions that the peer review comments apply to.

The versions of the paper that follow are the versions that were submitted during the peer review process. They are not the versions of record or the final published versions. They should not be cited or distributed as the published version of this manuscript.

BMJ Open is an open access journal and the full, final, typeset and author-corrected version of record of the manuscript is available on our site with no access controls, subscription charges or pay-per-view fees (http://bmjopen.bmj.com).

If you have any questions on BMJ Open's open peer review process please email info.bmjopen@bmj.com

BMJ Open

A protocol for long-term health and economic impact of using digital tool Gro Health W8Buddy to support people accessing specialist weight management services

Journal:	*BMJ Open*
Manuscript ID	bmjopen-2025-109111
Article Type:	Protocol
Date Submitted by the Author:	08-Aug-2025
Complete List of Authors:	Deo, Pranay; Warwick Medical School<br>Grove, Amy; University of Birmingham<br>Zhang, Mengxi; Warwick Medical School,<br>Abrams, Keith; University of Warwick, Department of Statistics; University of York, Centre for Health Economics<br>Auguste, Peter; University of Birmingham, School of Social Policy and Society<br>Barber, Thomas; University of Warwick; University Hospitals Coventry and Warwickshire NHS Trust<br>Gazeley, Tracy; University Hospitals Coventry and Warwickshire NHS Trust<br>Green, Richard; University Hospitals Coventry and Warwickshire NHS Trust<br>Griffiths, Frances; University of Warwick, Warwick Medical School<br>Hazlehurst, Jonathan; Birmingham Health Partners; University Hospitals Birmingham NHS Foundation Trust, Department of Diabetes and Endocrinology; University of Birmingham<br>Hee, Siew Wan; University Hospitals Coventry and Warwickshire NHS Trust, Research and Development; University of Warwick Medical School<br>Kaura, Amit; Imperial College London, Faculty of medicine<br>Mallipedhi, Akhila; Hywel Dda Health Board<br>O'Toole, Sarah; University Hospitals Coventry and Warwickshire NHS Trust<br>Panesar, Arjun; DDM Health,<br>Parsons, Nicholas; Warwick Medical School, Warwick Medical School<br>Scott, Emma; University of Warwick - Coventry Campus, Warwick medical school<br>Thind, Manreet; University Hospitals Coventry and Warwickshire NHS Trust<br>Summers, Charlotte; DDM Technology House University of Warwick Coventry, UK CV4 7EZ<br>Thorpe, Marie; University of Warwick, Warwick medical school<br>Zalin, Anjali ; Barts Health NHS Trust<br>Hanson, Petra; University of Warwick, Applied Health; University Hospitals Coventry and Warwickshire NHS Trust; South Warwickshire NHS Foundation Trust
Keywords:	Obesity, Digital Technology, Clinical Protocols, Mobile Applications

SCHOLARONE™
Manuscripts

A protocol for long-term health and economic impact of using digital tool Gro Health W8Buddy to support people accessing specialist weight management services

1. **Pranay Singh Deo** – University Of Warwick Medical School Coventry CV4 4AL pranay.deo@warwick.ac.uk
2. **Amy Grove** - A.L.Grove@bham.ac.uk - University Of Birmingham, Centre for Evidence and Implementation Science, Edgbaston, Birmingham B15 2TT
3. **Mengxi Zhang** - mengxi.zhang@warwick.ac.uk, University Of Warwick Medical School Coventry CV4 4AL
4. **Keith Abrams** - keith.abrams@warwick.ac.uk, University Of Warwick Medical School Coventry CV4 4AL
5. **Peter Auguste** - p.e.auguste.1@bham.ac.uk, University Of Birmingham Health Services Management Centre, Birmingham B15 2TT
6. **Thomas M Barber** - t.barber@warwick.ac.uk, Division of Biomedical Sciences, Warwick Medical School, University of Warwick, Coventry, CV4 7AL, UK Warwickshire Institute for the Study of Diabetes, Endocrinology and Metabolism, University Hospitals Coventry and Warwickshire, Clifford Bridge Road, Coventry, CV2 2DX, UK Research Centre for Health and Life Sciences, Coventry University, Richard Crossman Building, Jordan Well, Coventry, CV1 5RW, UK
7. **Tracy Gazeley** - tracy.gazeley@uhcw.nhs.uk, University Hospitals Coventry & Warwickshire NHS trust, Clifford Bridge Road, Coventry CV2 2DX PPI
8. **Richard Green** - rmguk@hotmail.com, University Hospitals Coventry & Warwickshire NHS trust, Clifford Bridge Road, Coventry CV2 2DX PPI
9. **Frances Griffiths -** f.e.griffiths@warwick.ac.uk University Of Warwick Medical School Coventry CV4 4AL
10. **Jonathan Hazlehurst** - jonathan.hazlehurst3@uhb.nhs.uk, University Hospital Birmingham Mindelsohn Way, Birmingham B15 2GW
11. **Siew Wan Hee** - s.w.hee@warwick.ac.uk, Warwick Medical School, University of Warwick, Coventry CV4 7AL, and Research and Development, University Hospitals Coventry and Warwickshire NHS Trust, Clifford Bridge Road, Coventry CV2 2DX
12. **Amit Kaura** - a.kaura@imperial.ac.uk, Imperial College London Exhibition Road, London SW7 2AZ , Faculty of Medicine
13. **Akhila Mallipedhi** - akhila.mallipedhi@wales.nhs.uk, Hyweldda Health Board, 1 Penlan Road, Carmarthen SA31 1DS
14. **Sarah O'Toole** - sarah.otoole@uhcw.nhs.uk, University Hospitals Coventry & Warwickshire NHS trust, Clifford Bridge Road, Coventry CV2 2DX PPI
15. **Arjun Panesar -** arjun@ddm.health, Diabetes Digital Mangement (DDM) Technology House Sir William Lyons Road, Coventry CV4 7EZ
16. **Nick Parsons** - nick.parsons@warwick.ac.uk, University Of Warwick Medical School Coventry CV4 4AL

For peer review only - http://bmjopen.bmj.com/site/about/guidelines.xhtml

17. **Emma Scott -** e.j.scott@warwick.ac.uk, University Of Warwick Medical School Coventry CV4 4AL
18. **Charlotte Summers** - charlotte@ddm.health, Diabetes Digital Mangement (DDM) Technology House Sir William Lyons Road, Coventry CV4 7EZ
19. **Manreet Thind** - manreet.thind@uhcw.nhs.uk, University Hospitals Coventry & Warwickshire NHS trust, Clifford Bridge Road, Coventry CV2 2DX
20. **Marie Thorpe** - marie.thorpe@warwick.ac.uk, University Of Warwick Medical School Coventry CV4 4AL
21. **Anjali Zalin** - anjali.zalin3@nhs.net, Barts Health NHS Trust, 9 Prescot Street, London E1 8PR
22. **Petra Hanson - Petra.hanson.1@warwick.ac.uk, University Of Warwick Medical School Coventry CV4 4AL** *

Corresponding Author: Petra Hanson - Petra.hanson.1@warwick.ac.uk, University Of Warwick Medical School Coventry CV4 4AL *

Trial Sponsor: University of Warwick, Coventry, CV4 7AL, 024 7652 3523

Version/08.08.2025

For peer review only - http://bmjopen.bmj.com/site/about/guidelines.xhtml

Abstract

Introduction: Obesity affects over a quarter of the UK population and can lead to serious health issues. NHS Specialist Weight Management Services offer treatments including lifestyle advice, psychological support, and medications, but access and availability vary by region. Although around 4 million people could be eligible for NHS Specialist Weight Management Services (WMS) annually, capacity is limited to 35,000, severely limiting overall access for those who need it. While digital technology has started to be used in WMS, more evidence is needed to confirm its long-term effectiveness, acceptability, and cost-effectiveness. This study explores the use of Gro Health W8Buddy, a digital platform and app providing remote Specialist WMS. It aims to determine the long-term health benefits of remote WMS pathway Gro Health W8Buddy compared to standard NHS WMS delivered in hospitals, and to improve patients access to services.

Methods and analysis: The study is a real-world evaluation with observational data collection. We will recruit 450 study participants from four NHS specialist WMS who will choose either standard NHS WMS or the digital pathway Gro Health W8Buddy. We will measure and analyse health outcomes including weight loss, time taken to be treated and cost-effectiveness, at 18 and 24-month. We will gather experiential data from patients and healthcare professionals through surveys, observation, and interviews.

Ethics and dissemination: Ethical approval has been obtained from NHS Health Research Authority (HRA) and Health and Care Research Wales (HCRW) (REC reference: 25/EM/0147). Our findings will be disseminated through academic publications, conference presentations and stakeholder engagement.

Strengths and limitations of this study:

- The study is structured around four distinct work packages, each employing a unique research methodology
- The digital intervention has been co-created with NHS staff and patients and is backed by research
- This research methodology has been shaped by PPI input from all four NHS sites
- The study methods enable Real World Evidence to be generated by allowing participants to choose their own pathway and will therefore provide credible evidence for further roll out of digital technology in weight management services across the UK
- Allocation is, by design, not randomised which may lead to more participants in digital care or vice versa

For peer review only - http://bmjopen.bmj.com/site/about/guidelines.xhtml

Introduction

Background

In the UK, a quarter of adults live with obesity, with higher rates among people from Black and Asian ethnic groups and people living in the most deprived areas (1). People living with obesity are at increased risk of chronic diseases including Type 2 Diabetes Mellitus (T2DM) and cardiovascular disease as well as at risk of shorter life expectancy (2). Treatment of obesity cost the NHS £6.5bn in 2022, with an associated ill health annual full cost of £58bn (3). The NHS and Office for Life Sciences (OLS) identified treatment and prevention of obesity as one of its healthcare missions (4).

A tiered approach to weight management exists in the UK. Tiers 1 and 2 are delivered in community and primary care. Tier 3 is delivered by specialist Weight Management Services (WMS) which are often based in a hospital setting. WMS comprise of multidisciplinary teams who provide dietetics, psychology, and consultation by specialist doctors. Tier 4 services include bariatric surgery (5). In the UK, NICE recommends that people with BMI over 40 kg/m^2 or over 35 kg/m^2 with medical problems (e.g., T2DM, hypertension) are eligible for referral to WMS (6) however there is geographical variation, with many areas lacking access to services (7).

Outcomes from traditional face-to-face WMS are mixed, with 50% of patients in most NHS WMS achieving a 5% weight loss over 6-12 months according to a recent review (8). Retention rates are low (<55%) and there is little long-term (>12 months) data on sustainability of weight loss (8). WMS have limited capacity for just 35,000 patients (9), yet 4 million people are eligible for services. This represents a significant capacity issue (10). Due to these access constraints only 3.13% of this population receive a referral to WMS (9). There is also marked regional variation in service design and accessibility (7).

For peer review only - http://bmjopen.bmj.com/site/about/guidelines.xhtml

In March 2023, NICE recommended the obesity medication semaglutide (Wegovy) for people accessing WMS (11). Additionally, tirzpatide was recommended for obesity treatment from December 2024 (12). These treatments represent a step change in Tier 3 WMS services and are part of the government-backed pilot to widen access to this medication with the use of digital technology (13). However, guidance on the incorporation of digital technology to address how to safely incorporate the drugs remains unclear. We aim to fill the gap with this study.

Digital technology for managing chronic disease, including obesity, is expanding rapidly in the NHS (14). Acceptability of digital technology for weight loss is high in both community and WMS settings (15-18). However, effectiveness varies. Evidence shows weight loss ranging from 13% at 4-month (digital + meal replacements) (19) to 7.6% at 12-month (digital + coaching) (20). A community based digital weight management intervention recorded 5% weight loss at 2 years in 18% of users (18). According to the evidence, there is a gap and need for further digital weight management research to investigate standard and digital care, alongside the integration of pharmacotherapy (e.g., semaglutide) and the use of a multi-disciplinary team (21). Currently the evidence on the digital divide created by digital technology for weight management is lacking (21). Therefore, innovative approaches are needed in the NHS WMS to reduce health inequality, address efficiency, increase capacity, and improve access to pharmacotherapy for obesity.

As a result of the above evidence, we co-created a digital support tool *Gro Health W8Buddy* (W8Buddy) with a digital company Diabetes Digital Media (DDM) to provide digital technology to deliver NHS WMS (7). W8Buddy provides personalised WMS to support patients to achieve their self-selected health goals and support weight loss with real-time remote monitoring. The creation of W8Buddy was supported by an *HEE Topol Digital*

For peer review only - http://bmjopen.bmj.com/site/about/guidelines.xhtml

Fellowship and *NHS Clinical Entrepreneur scheme* (22). Evidence has shown that W8Buddy helps to reduce weight, improve glycaemic control and wellbeing among people living with obesity, overweight or with T2DM (23, 24). W8Buddy was endorsed by *NICE Early Value Assessment* for Digital Technologies in Specialist Weight Management in October 2023 (25).

Our initial 1-year evaluation of 412 patients showed that 220 patients using W8Buddy (version 1 – information resource) *alongside* traditional WMS lost significantly more weight (0.75 kg/month) than 192 patients using traditional WMS (34). Since then, W8Buddy has been adapted to provide a fully remote WMS. This study aims to provide longer term real-world evidence (RWE) of the clinical and cost-effectiveness of the adapted W8Buddy in the NHS.

Research question: What is the long-term (18-month) impact on clinical outcomes, including health-related quality of life (QoL) and cost-effectiveness of W8Buddy with and without weight management medication and lifestyle support to people living with obesity, compared with WMS standard care?

Objectives

1. To generate longitudinal evidence of the clinical and cost-effectiveness of the W8Buddy from four NHS WMS, University Hospital Coventry & Warwickshire (UHCW), University Hospitals Birmingham (UHB), London Luton Trust (LTT) and Hywel Dda University Health Board Trust (HDUHBT), using the digital pathway W8Buddy.
2. To generate sufficient evidence to satisfy NICE guidance requirements (25).
3. To understand the process of implementation and use of W8Buddy within WMS.

For peer review only - http://bmjopen.bmj.com/site/about/guidelines.xhtml

4. To create a manual for safe incorporation of a digital pathway delivering weight management medication into existing WMS across the UK.
5. To make recommendations for how we can optimise existing infrastructure of WMS to improve ongoing real world evidence generation.

For peer review only

For peer review only - http://bmjopen.bmj.com/site/about/guidelines.xhtml

Methods

Study Design

Multi-site prospective comparative observational cohort study with embedded cost-effectiveness analysis and process evaluation. Comprising of four work packages (WP) to assess the following; (1) Set up, Patient and Public Involvement and Engagement (PPIE) & Stakeholder engagement, (2) Clinical impact, (3) Health economic impact and (4) Process evaluation.

Eligibility criteria to enter WMS

Inclusion criteria reflect the eligibility of people to enter the clinical services participating in this research, including services with additional eligibility criteria restrictions (subject to local processes that may differ slightly). However, whilst individual services may have further restrictive criteria, which is reflective of many WMS all participants are included within:

- Adults aged 18 years and above
- Class 2 obesity (BMI ≥ 35kg/m^2) with at least 1 medical complication (e.g., prediabetes, type 2 diabetes (T2DM)); or patients with obesity class 3 (BMI ≥ 40kg/m^2), without any complications.

The eligibility criteria for access to Tier 3 WMS for adults from South Asian, Chinese, other Asian, Middle Eastern, Black African, or African-Caribbean background have BMI cut offs 37.5 kg/m^2 without comorbidities.

Exclusion criteria:

For peer review only - http://bmjopen.bmj.com/site/about/guidelines.xhtml

For peer review only

- Pregnant or breastfeeding
- Type 1 diabetes mellitus
- End stage renal disease (eGFR<15, receiving renal replacement therapy)
- Active or untreated alcohol or drug dependency
- Decompensated liver disease
- History of anorexia, undergoing treatment or awaiting treatment for DSM-V classified eating disorder
- Severe mental health disease, including acute mental health crisis or self-harm in the last 12 months
- Unstable mental health disease including unstable personality disorder, schizophrenia, and bipolar disorder
- Unwilling or unable to commit to participation across the length of the research study.
- Patients targeting bariatric surgery within the length of the research study.
- Participating in another weight management research intervention
- Participating in the diabetes remission program

Study setting and sites

This study will run at four NHS sites (UHCW, UHB, LLT, HDUHBT). Standard care differs across the recruiting sites, including the mixed provision of obesity medication. Sites without medication as standard care will be noted as the digital pathway *without medication.*

For peer review only - http://bmjopen.bmj.com/site/about/guidelines.xhtml

Patient and public involvement (PPI)

A W8Buddy PPI Group has been established with a minimum of four PPI Representatives with lived experience of WMS from each site. Potential PPI representatives have been identified through WMS teams at each participating site and obesity charities. Those interested in joining the groups are invited to meet with the PPIE Lead and/or UHCW PPIE Team to discuss the opportunity, commitment required, and support offered and to find out about their experience and background. PPI representatives selected represent the diverse population of the WMS users in each area. The Group meet quarterly to ensure ongoing input from people with lived experience, including identifying outcome measures that are meaningful for people with obesity, providing feedback on participant facing documents, working on creating local pathways and training materials. The PPI Group will also shape analysis and risk mitigation, may take part in conducting semi-structured interviews as part of qualitative research, and support dissemination of findings afterwards.

Sample size calculation

We will collect weight data at baseline and a minimum of three occasions during the study, for both pathways. Powering the study to show that the digital pathway is non-inferior to the standard of care pathway, with a difference less than 4kg, at 90% power and a two-sided 5% level, gives a sample size of approximately 180 participants. Further assuming a correlation between the repeated weight measurements in the range 0.2 to 0.5, which previously collected data suggests is reasonable (34), based on a longitudinal mixed model/repeated measures ANCOVA gives a total sample size of 360. Conservatively assuming 20% loss during follow-up, the target sample size is 450.

Given the current widespread use of the digital (W8Buddy) intervention at the lead centre (UHCW), the control (standard of care; SC) intervention at this site will be different from that

For peer review only - http://bmjopen.bmj.com/site/about/guidelines.xhtml

offered at the other sites. For this reason, we plan to characterise the control intervention at UHCW as a “hybrid” digital intervention. We will aim to recruit sufficient participants (i.e. 360) at the three other sites (UHB, LLT, HDUHB) to fully power the study for the planned comparison between the digital and standard of care groups. Additionally, we will collect data from the “hybrid” digital intervention at UHCW to augment and further refine the primary analysis.

Screening, recruitment, and consent

Potential participants will be identified by research nurses once they have been referred (or self-referred) to the local Tier 3 WMS. Participants will be screened electronically against pre-specified criteria.

Eligible participants will be able to choose how they receive their WMS - either in the standard NHS delivery, or via fully digital platform Gro Health W8Buddy. Site specific standard of care pathways will be delivered by the usual health care professionals, employed by each NHS site. The digital pathway will be delivered by health care professionals employed by DDM (providers of Gro Health W8Buddy). We aim to recruit a total of 450 participants across both arms from September 2025 to February 2026.

Those who satisfy the study inclusion criteria will be contacted by the research team via telephone. Brief information about the study will be provided to potential participants, including a link to a website with information about the study. This website will be password protected as only eligible participants will be able to view it. Digital information sheets and consent forms will be hosted on the website.

For peer review only - http://bmjopen.bmj.com/site/about/guidelines.xhtml

If a patient is unable to access the website (therefore, unable to complete electronic consent) the local site research nurse will invite them to the hospital. They will show the patient the research website and help them with electronic consent. Later, the research nurse will print out the consent form (signed copy) only for the patient if they do not have access to email.

Electronic consent for data collection (24 months), with an option to consent for ongoing data collection, will be obtained. Following informed consent, participants will be able to choose the digital or standard care pathway via the website. A copy of the signed consent form will be sent to the participants, local research team and site Principal Investigator (PI).

To support data collection in the standard care pathway, participants will be offered a £10 voucher for each completed survey at month 0, 6, 12, 18 and 24; vouchers will not be provided for just signing up to standard care, surveys must be completed.

Intervention Gro Health W8Buddy

Gro Health W8Buddy is a virtual specialist WMS pathway. It is accessed through a digital behavioural change platform called the Gro Health app (see, Figure 1). Participants on the W8Buddy digital arm will receive 52 weeks of support from health care professionals employed by DDM. At week 52 each participant on digital pathway will be assessed and the following options offered: discharge to primary care, onward referral to local Tier 4 if interested in surgery, onward referral for local Tier 3 MDT discussion, extension of digital pathway by 6 months, referral to local Tier 2 weight management service.

Figure 1: Gro Health W8Buddy platform and app

For peer review only - http://bmjopen.bmj.com/site/about/guidelines.xhtml

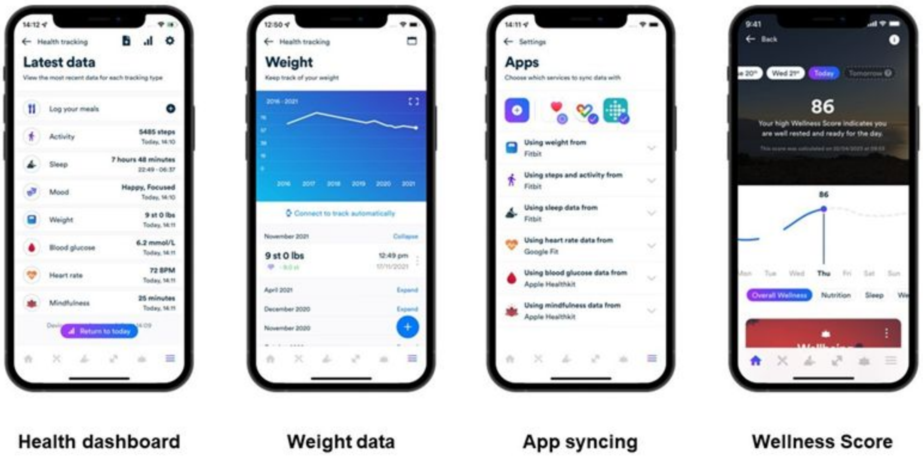

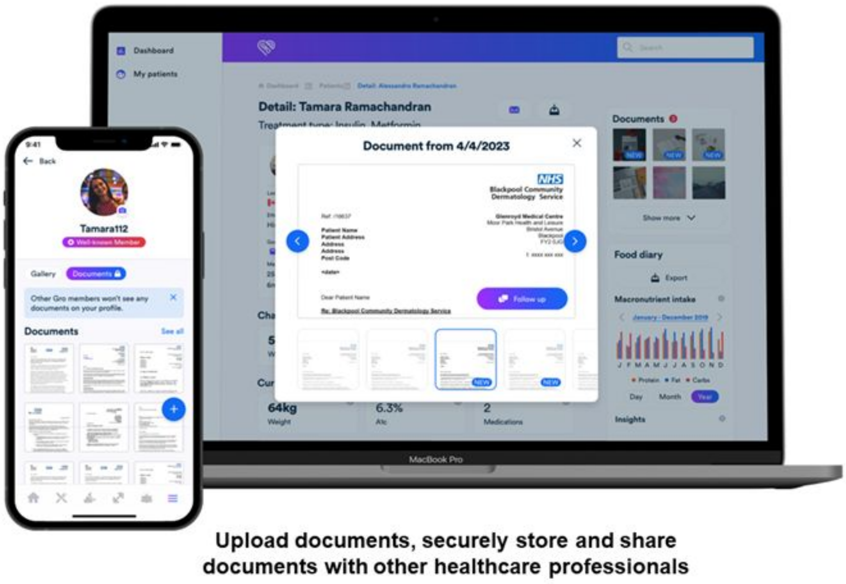

Standard care

Participants in the standard care arm will receive the tier 3 WMS that is delivered by their local NHS trust. All four NHS trusts included in the research have differences in their tier 3 service, which will be explored in detail as part of the study. The differences range from the amount of input from healthcare professionals (psychologist, dietitian, physician), modality

For peer review only - http://bmjopen.bmj.com/site/about/guidelines.xhtml

of sessions (1-1 or group sessions) and frequency. Post trial care for participants in standard care will be delivered in accordance to their NHS trust policies for tier 3 weight management.

Figure 2: Participant flow chart

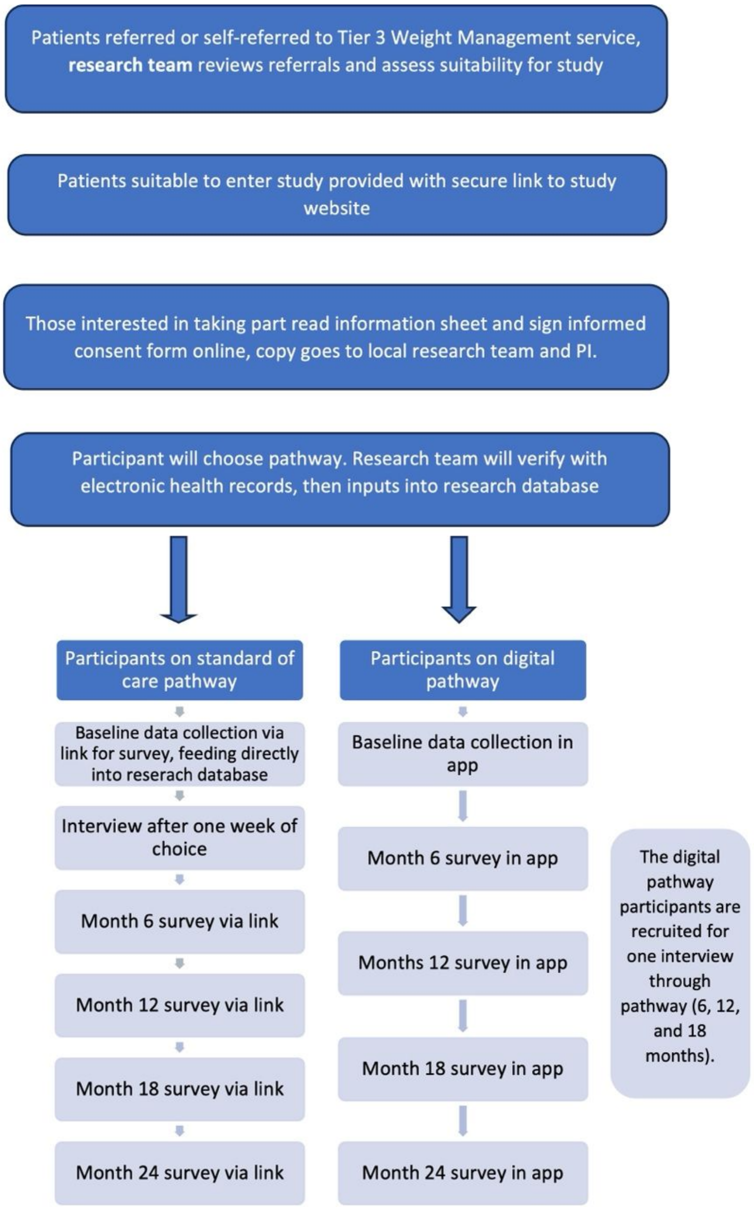

Assessment and management of risk

Risks relate to data management/reporting across 4 sites and management of the digital pathway delivered by DDM. The latter is assured by DDM. Table 1 lists these.

For peer review only - http://bmjopen.bmj.com/site/about/guidelines.xhtml

Table 1 Assessment and management of risk

Data reporting	Not reporting data by participants, mitigated by regular support via the app or contact from research team if not completing surveys.
Data protection	As with any online service proper data handling is assured by high security data storage in line with NHS Digital policies. Data from NHS sites transferred to analytical partner using secure pathways.
Delivery	The site research team will oversee all participants, including those accessing digital pathway. The first contact for any queries for participants on digital pathway will be the digital provider, and if the query is not resolved the research site team will be contacted. The project will be managed by PH and AG via weekly WP meetings between sites and project manager, with monthly oversight by each site PI. Three-monthly meetings of PPIE group will ensure ongoing input from patients. Annual project steering committee meetings will review risk registers.
Imbalanced recruitment	More patients opting for digital rather than standard of care pathway (or vice-versa), mitigated by over-recruiting standard of care (or digital) patients and adjusting as per analysis plan using propensity-weighted approach, purposive sampling and ongoing monitoring of recruitment, communication to sites, telephone and offline packs as another option for those choosing digital pathway.
Engagement with the digital pathway	Low engagement from high-risk/target groups resulting in lower enrolments and participation from those most in need of the service. Mitigated by patient friendly resources, onboarding support, research nurse at each site to act as a point of call.
Patient safety	Without face-to-face contact, healthcare professionals may miss important physical signs and symptoms that might indicate a health concern for study participants. Participants might also misunderstand or misuse instructions regarding their medication, increasing the risk of side effects or adverse events. This is mitigated through symptom tracking, face-to-face videoconferencing/virtual calls, identifying signs of body language, and recording sessions for audit to ensure ongoing patient safety. Digital phenotyping of patients occurs in real-time, and any thresholds of anxiety or depression are noted in the clinical dashboard and the clinical team are notified. Omni-directional communication through the platform allows clinicians to engage directly with patients and vice-versa with up-to-date, real-time data. Patient expectations are managed by informing the patient if their coach

For peer review only

For peer review only - http://bmjopen.bmj.com/site/about/guidelines.xhtml

	is offline and encouraging them to seek emergency medical assistance if at risk.
Medication safety on digital pathway	Prescribing medication remotely requires a comprehensive understanding of the patient's current health status, medications, and lifestyle, which can be challenging to obtain in a virtual environment. There is also the risk of potential drug interactions that might be overlooked without a detailed in-person evaluation. This is mitigated through patient completion of psychological assessments, medication eligibility assessment, and approval for medication from the multidisciplinary team (MDT) team providing the patient's care. Regular reviews ensure medication is not mismanaged and appropriate for the patient. Patient is provided with videos and resources on how to inject.
Psychological safety on digital pathway	Weight management is closely tied to self-esteem and body image, which can be negatively impacted if the focus is solely on weight loss. Additionally, the digital nature of virtual services can exacerbate feelings of isolation and lack of support. This is mitigated through support from a psychotherapist/psychologist throughout the programme, access to private/group coaching for on-demand support, and artificial intelligence (AI) algorithms that provide digital phenotype measurements for a patient's likelihood of anxiety, stress, and depression based on conversations/engagements in the community.
Eating disorders	Weight loss can trigger or exacerbate eating disorders. For individuals with a history of eating disorders, weight loss medications could potentially be misused in a harmful way. This is mitigated through patient completion of psychological assessments, medication eligibility assessment, and approval for medication from the MDT team providing the patient's care. On the digital pathway, regular reviews with a pharmacist and psychologist ensure medication is not mismanaged and appropriate for the patient. Signposting and referral back to the GP will be provided to any patients who display signs of disordered eating. On the standard of care pathway, local protocols will be followed.
Engagement with the standard of care pathway	High drop out from the standard of care pathway: there is a high drop out from the standard of care service in Tier 3 Weight Management, resulting in high loss to follow up. This will be mitigated by having a dedicated research nurse who will contact patients to ensure they complete all data collection in timely fashion. Additionally, participants in the standard of care will receive vouchers to compensate them for the additional surveys they will be asked to do.

For peer review only - http://bmjopen.bmj.com/site/about/guidelines.xhtml

Outcomes

The primary outcome is changes in body weight after 18-months of using either (i) a fully digital weight management programme (remote digital pathway Gro Health W8Buddy) or (ii) site-specific delivered weight management programmes incorporating face to face and/or virtual group sessions (standard care).

Secondary outcomes are metabolic markers (HBA1c, lipids), blood pressure, waist circumference and health related quality of life (EQ-5D-5L) (35).

For peer review only

For peer review only - http://bmjopen.bmj.com/site/about/guidelines.xhtml

Data collection

In Table 2 we detail what baseline and follow up data will be collected to assess primary and secondary outcomes. During the process evaluation, economic evaluation, additional cost, and qualitative data will be collected.

Table 2 ***Types of outcome measures and data collected***

**Data collected**	**When and how data collected from digital (D)/ Standard care (SC) group**	**Data used for assessment of:**
**Weight, height, and BMI**	Month 0,6,12,18,24.<br><br>D: app, dashboard<br><br>SC: survey, clinical notes	Weight loss
**Metabolic markers (HBA1c, lipids)**	Month 0,6,18.<br><br>D: app, dashboard (the same standard of care blood tests will be done remotely instead using clinical phlebotomy partners and samples will be returned for analysis, then discarded as per usual clinical procedures.<br><br>SC: survey, clinical notes (only routine blood test results will be collected in the standard of care pathway, as per usual standard of care for NHS hospitals)	Metabolic impact

For peer review only - http://bmjopen.bmj.com/site/about/guidelines.xhtml

**Blood pressure**	Month 0,6,18.<br><br>D: app, dashboard<br><br>SC: survey, clinical notes	Cardiovascular marker
**Waist circumference**	Month 0,6,12,18,24.<br><br>D: app, dashboard<br><br>SC: survey, clinical notes	Body composition changes
**Health related quality of life (EQ-5D-5L)**	Month 0,6,12,18,24.<br><br>D: app<br><br>SC: survey	Cost-effectiveness and quality of life
**PHQ8, satisfaction with life scale, Karolinska sleepiness scale, GAD7, BEDS-7**	Month 0,6,12,18,24.<br><br>D: app<br><br>SC: survey	Biopsychosocial outcomes
**Clinical endpoints (stroke, myocardial infarction)**	Month 12, 24, 36, 48, 60<br><br>D: app, dashboard<br><br>SC: clinical notes	Major adverse cardiovascular events
**Distance to local NHS facility for participants**	Month 0.	Health economic calculations

For peer review only - http://bmjopen.bmj.com/site/about/guidelines.xhtml

	Research team review of GP notes for D and SC	
**Time needed to travel to local NHS facility for participants**	Month 0.<br><br>Research team review of GP notes for D and SC, plus<br><br>D: app<br><br>SC: survey	Health economic calculations
**Date when participants dropped out of study if applicable**	As applicable.<br><br>D: app<br><br>SC: survey	Accurate tracking
**Reason why participant on standard care dropped out of study if applicable**	SC: Contact with research nurse	Why participants drop out of standard care
**Number of consented participants**<br><br>• **choosing the digital pathway**<br><br>• **starting on the pathway**<br><br>• **dropping out**	Month 0-24.<br><br>Reviews of dashboard and research database	Willingness and engagement level of digital pathway<br><br>Sampling frame for participant interviews
**Participant baseline characteristics: comorbidities, sex, age, ethnicity, disabilities, geographical region, socioeconomic status**	Month 0.	Stratification of interview sample, sensitivity analyses, predictors of cost-effectiveness and engagement with digital pathway

For peer review only - http://bmjopen.bmj.com/site/about/guidelines.xhtml

	Research team review of GP notes for D and SC, plus<br><br>D: app<br><br>SC: survey	
**Comorbidities at follow up (hypertension, obstructive sleep apnoea, osteoarthritis, non-alcoholic fatty liver disease, polycystic ovarian syndrome, type 2 diabetes, dyslipidaemia)**	Month 6, 12, 18, 24<br><br>D: app, review of health records<br><br>SC: survey, review of health records	Assessment of diseases associated with obesity
**Type of concomitant and weight-management medication, dose, and prescription dates, whether NHS or non-NHS prescribed**	Month 0,6,12,18,24<br><br>D: app<br><br>SC: survey, clinical notes	Calculation of cost<br><br>Medication adherence
**Reason for stopping the medicine, including side effects (including physical and psychological effects as per STEP 5 trial. These include nausea, diarrhoea, constipation, vomiting, abdominal pain, dyspepsia, gastroenteritis, decreased appetite, headache, eructation, nasopharyngitis, headache, back pain, upper respiratory tract infection, gallbladder related disorders, cholelithiasis, pancreatitis,**	Month 0,6,12,18,24<br><br>D: app, dashboard<br><br>SC: survey, clinical notes	Cost-effectiveness, Biomedical explanation of adherence rates

For peer review only - http://bmjopen.bmj.com/site/about/guidelines.xhtml

**cardiovascular disorders, injection site reactions, hypoglycaemia, psychiatric disorders , reaching predefined weight loss goals, lack of adherence to prescribed medicine**		
**Use of and response to digital pathway**	Month 0-24<br><br>D: app<br><br>SC: appointment records and clinical notes	Cost-effectiveness,<br><br>Adherence to digital pathway; variation in adherence by patient characteristics<br><br>User experience and process evaluation

For peer review only

	Information collected on all patients who switch from SC to D and vice versa, even though this is expected to be low	
**Adverse events, including only intervention-related adverse effects**	Month 0-24<br><br>D: consultation with clinicians on dashboard, medication tracking, captured at real-time<br><br>SC: clinical notes, surveys at month 6,12,18,24	Cost-effectiveness, understanding the experience of users, creating blueprint for safe incorporation of digital pathway
**Healthcare resource use: Data from providers about the multidisciplinary service, resource use (e.g. rates of referral to bariatric surgery)**<br><br>**Data from participants on number of GP visits, unplanned admissions to A&E**	Month 0,6,12,18,24<br><br>D: app and reports from digital provider<br><br>SC: survey, clinical notes	Cost-effectiveness
**Cost associated with implementing and maintaining digital pathway**	D: Digital provider	Cost-effectiveness
**Cost estimates for specialist WMS**	Month 0,6,12,18,24	Cost-effectiveness

For peer review only - http://bmjopen.bmj.com/site/about/guidelines.xhtml

	SC: clinical records, survey	
**Implementation, adaptations, and contextual influences**	Observation of pathway delivery team meetings, meeting notes, semi-structured interviews with implementers and clinical staff delivering intervention.<br><br>During initial implementation (month 0) and in months 12 & 18	Implementation process to inform formative feedback<br><br>Manual for future NHS implementation and process evaluation
**Availability of digital pathway and medicine to participant including**<br><br>**staff training and quality assurance**	D: app and related organisational records<br><br>SC: clinical records and related organisational records throughout delivery	Fidelity of delivery of intervention<br><br>Experience of delivery of intervention<br><br>Process evaluation
**Brief structured interview with study participants not choosing digital pathway**	Within approx. 1 week of choice<br><br>Remote interview	Why participants do not choose digital pathway (process evaluation)
**Brief structured interview with study participants not adherent to digital pathway and/or medicine**	Sample for participants demonstrating early/late non-adherence or on/off adherence<br><br>Remote interview	Why participants drop out/return to digital pathway/medicine process evaluation)
**Semi-structured interviews with study participants using digital pathway**	Sample stratified by gender and progress through pathway (6, 12, 18 months)<br><br>Remote interview	Understand how user engages with digital pathway, why, perceived impacts and user understanding of mechanism of impact (process evaluation).

Abbreviations: BMI, Body mass index; **EQ-5D-5L**, EuroQol five dimensions and levels; GAD, general anxiety disorder scale; PHQ-8, Patient Health Questionnaire 8, BEDS-7, Binge eating disorder screener

For peer review only - http://bmjopen.bmj.com/site/about/guidelines.xhtml

Monitoring

The study will be monitored by the study management group and the University of Warwick representative of the Sponsor to ensure that the study is being conducted as per protocol, adhering to Research Governance and Good Clinical Practice (GCP). Central monitoring activities will be performed such as data quality checks. The recruiting site is obliged to assist the sponsor in monitoring the study. These may include hosting site visits, providing information for remote monitoring, or putting procedures in place to monitor the study internally. The approach to, and extent of, monitoring will be specified in a study monitoring plan determined by the risk assessment undertaken prior to the start of the study.

Analysis

Primary outcome analysis

The primary analysis will compare temporal changes in weight for the W8Buddy and standard care group, from baseline to completion of follow-up (at 18 months), using longitudinal mixed effects regression models. The effectiveness of the W8Buddy will be quantified by differences in rates of weight change compared to standard of care during follow-up.

Statistical analysis plan A detailed statistical analysis plan will be prepared by the statisticians, and agreed with the study team, prior to final analysis, and which will follow the NICE RWE Framework (26). Comparisons will be primarily on an as-started (intention to treat, ITT) basis. We do not expect any or only minor levels of treatment switching or discontinuation for the W8Buddy group. Therefore, we would expect the on-treatment and as-started treatment effect estimates to be equivalent in this population. We will report the result according to the STROBE guideline (27).

For peer review only - http://bmjopen.bmj.com/site/about/guidelines.xhtml

Summary of baseline data Demographic and baseline data will be summarised overall and by treatment arms and sites. Categorical data will be summarised as count and percentage, and continuous data will be summarised as number of missing data, mean with standard deviation, and median with 25th and 75th percentiles.

Missing data Patterns of missing data/discontinuation will be reported and assessed if missingness was at random or provided useful information on treatment efficacy.

Secondary outcome analysis We will consider adjusting for a range of demographics and clinical characteristics (e.g. age, gender, ethnicity, IMD/WIMD, comorbidities, NHS site, mode and distance of travel and other confounders identified using Directed Acyclic Graphs with stakeholders PPI members and members of the steering committee) (28) using propensity-weighted longitudinal mixed models. We expect the fixed effects to be demographics and clinical characteristics as mentioned above and the random effects to be the sites and individuals. A sensitivity analysis using "distance to NHS site" as an instrumental variable (29) will also be conducted to assess the assumption of no unmeasured confounders.

The comparison between the hybrid intervention and both the standard of care and fully digital intervention will be an unpowered secondary analysis, that we hope will offer additional insights and help to characterise the magnitude of intervention effects from the primary analysis.

Economic evaluation

We will develop a costing toolkit to provide transparent resource use and costs for the delivery of the standard care and digital delivery pathways. The toolkit development process

For peer review only - http://bmjopen.bmj.com/site/about/guidelines.xhtml

will comprise three stages: planning, data collection and synthesis/analysis (30, 31). We will require resource use and cost estimates for delivery of W8Buddy providing weight management medication and standard of care (Tier 3 WMS). We will produce a systematic review of cost effectiveness literature to inform the development of a decision-analytic model. The model will be used to estimate the cost-effectiveness of Tier 3 WMS in addition to W8Buddy compared to weight management services delivering weight management medication to support people with obesity. This will be fundamental to help to understand the long-term cost-effectiveness of weight management programmes as they allow for the extrapolation of the short-term benefits of interventions.

Process Evaluation

A mixed methods process evaluation will be conducted to understand the implementation of W8Buddy. Process evaluation data is listed in Table 2 above.

Electronic data from intervention and control pathways: We will collate data from the electronic clinical records, intervention, and study records of all study participants data on:

- Fidelity: delivery of coaching sessions, check-ins and physical activity sessions and progress reviews
- Adherence: Including biomedical explanations of adherence (comorbidities), we will assess user adherence to each consultation in the digital pathway (a patient attending 75% of consultations or more is considered adherent)
- Who chooses the digital pathway or not
- Who engages with the digital pathway, their level of engagement and predictors of engagement

For peer review only - http://bmjopen.bmj.com/site/about/guidelines.xhtml

Qualitative data collection and analysis

We will record observation data as field notes and collate documents. Qualitative data will be obtained from participants interviews via telephone or videoconference. We will audio record the interviews, transcribe using software and check the transcripts against the audio recording. We will undertake thematic analysis (32). This analysis will be undertaken independently of the quantitative analysis team.

Initial implementation and health professional experience of delivering digital pathway

We will observe site team meetings and implementation of the intervention, access team documents and undertake interviews with team members implementing the digital pathway to understand how they are meeting implementation challenges, training delivered, and quality assurance processes put in place. During the observations, the patient will be asked for permission for the researcher to remain present during the health professional-patient interaction. If this is refused, the researcher will leave.

In months 12 and 18 we will interview those delivering the digital pathway to understand their experiences, how they adapt WMS and any unexpected pathways or consequences (estimate 5/site). We will use implementation frameworks (33) to guide thematic analysis of implementation data whilst remaining alert to new themes. Early analysis of data on implementation will be used to provide formative feedback for subsequent intervention site teams.

User experience of digital pathway

For peer review only - http://bmjopen.bmj.com/site/about/guidelines.xhtml

In semi-structured interviews with users, we will explore their experiences of engaging with the digital pathway, influences on their engagement, challenges and how these were met, adaptations, impact, and mechanisms of impact (estimate 10/site, total 40).

We will undertake brief structured interviews with participants not choosing the digital pathway (5/site) and participants not adherent to the digital pathway and/or medicine (5/site). We will ask participants why they did not choose the digital pathway/drop out or return to the digital pathway/medicine. We will develop our brief structured questions with input from the study PPI group to ensure neutral, non-judgemental phrasing. We will sample participants consecutively across study sites and approach them for recruitment at least a week after their decision.

Describing the control intervention patient pathway

A researcher will visit each site to observe the control intervention. The team providing the control intervention will provide a written description of the control intervention patient pathway. From observation and conversation, the researcher will add additional details and variation on that pathway. The study team will analyse the observation notes to compile a description of the control intervention and check this with staff delivering the control intervention. Study sites will extract from the appointment system and relevant clinical records of their control intervention pathway, patient adherence data in terms of attendance at appointments, whether they are individual or group and duration of appointments, and use of medication.

Ethics and dissemination Ethical approval for this study has been obtained from the Health Research Authority (HRA) Research Ethics Committee (REC) and Health and Care Research

For peer review only - http://bmjopen.bmj.com/site/about/guidelines.xhtml

Wales (HCRW) (REC reference: 25/EM/0147). Informed consent will be required to enter the study, as well as to share anonymised data with local health care team and study team. Participants will be consented for long term follow-up using routinely collected data. Protocol amendments (if any) will be reported and discussed with National Institute for Health and Care Research (NIHR) and local ethics committee.

Study data will be collected on the screening logs, W8Buddy App, and validated questionnaires (see Table 2) electronically. Health record data for each site will be accessed by local research team. An Electronic Data Capture system will be used to record and store study data transferred securely from the W8Buddy App and hospital medical records. This will be the main Study Database. Only research team and members of the DDM team will have access to it. They will be listed in the delegation log and must hold Good Clinical Practice (GCP) certification. Audio recordings of interviews will be stored on secure University of Warwick computers and accessed only by research team located at Warwick.

We have a tiered approach to dissemination to ensure our findings reach participants, policy makers and practitioners. We will host a stakeholder engagement dissemination event at the end of the study to maximise the reach of our finding.

Our results will inform UK guidelines (incorporation of digital tool delivering pharmacotherapy into obesity management guideline). Academic publications will include protocol paper, main results of RWE evaluation, the health economic assessment and a paper describing the scale-up in NHS services and impact on multiple long-term conditions. We will create NHS business case, build on ongoing partnership developed across four adoption sites, produce equality and health inequality impact assessment, and have evidence for health and financial impact to support NHS adoption. We will improve NHS services by facilitating the testing and scale-up of our digital innovation which can support other Trusts wanting to

For peer review only - http://bmjopen.bmj.com/site/about/guidelines.xhtml

For peer review only

deliver digitally enabled services. This will include recommendations to other service providers, targeted education, and creation of a virtual learning environment (hosted by an establish Warwick University platform). Our study outputs will provide evidence to apply for a NICE review of clinical guidelines and an update to NHS reimbursement criteria, which will open the door to national adoption by weight management services.

For peer review only

Discussion

Research is needed to determine how we can most effectively deliver WMS to more people (21). One potential solution to address issues of patient access is the introduction of digital services (14-18). However, long term evidence about clinical and cost-effectiveness is required before digital pathways are fully integrated into NHS WMS across the whole country.

In this study we aim to provide longer term real-world evidence (RWE) of the clinical and cost-effectiveness, as well as barriers and facilitators of uptake of W8Buddy digital pathway implemented in four NHS sites. All these potential findings are essential considering the current data on the demand for WMS, as well as the health and economic impact of obesity (2,3). Guidance on the incorporation of weight management drugs alongside digital interventions is also an unclear gap we intend to fill with our research. With capturing the

For peer review only - http://bmjopen.bmj.com/site/about/guidelines.xhtml

variation that exists amongst our four NHS sites, we will provide guidance on implementation into further NHS sites.

Allowing study participants to choose their own pathway will allow RWE to understand uptake of the digital pathway when incorporated into standard care. Furthermore, we are assessing whether W8Buddy can decrease NHS waiting times and costs. Individuals living with obesity could in time be supported virtually without the need to attend in person specialist services with the associated costs and challenges of access. Investigating pathways, waiting times and accessibility is important for Obesity research considering the low retention rates and high demand but low availability in WMS (7,9,10). The use of a digital pathway will also provide further insight for innovative diagnostic pathways for obesity management, such as moving from acute to home care and being offered to more patients in a cost-effective way, which would further contribute to the literature base.

We acknowledge the challenges that may occur with the adoption of a digital pathway into NHS sites such as recruitment rates, levels of engagement and differences in WMS at NHS sites. However, capturing any challenges that may arise and providing solutions will benefit the future implementation of W8Buddy and contribute to generalisable knowledge.

For peer review only

Conclusion

We aim to improve NHS Specialist WMS services by facilitating the evidence generation and scale-up of digital innovation Gro Health W8Buddy. This could in turn support other NHS organisations wanting to deliver digitally enabled services. The findings of this study will be used to generate recommendations for other service providers, targeted education, and creation of a virtual learning environment. It is aimed the study outputs will provide evidence

For peer review only - http://bmjopen.bmj.com/site/about/guidelines.xhtml

needed for full NICE technology appraisal, as well as evidence for NHS commissioners to facilitate national adoption of technology in specialist WMS.

For peer review only

References

1. Baker C. Obesity statistics 2023 [Available from: https://researchbriefings.files.parliament.uk/documents/SN03336/SN03336.pdf.

For peer review only - http://bmjopen.bmj.com/site/about/guidelines.xhtml

2. Peeters A, Barendregt JJ, Willekens F, Mackenbach JP, Al Mamun A, Bonneux L. Obesity in adulthood and its consequences for life expectancy: a life-table analysis. Ann Intern Med. 2003;138(1):24-32.

3. Economics F. Estimating the full costs of obesity 2022 [Available from: https://www.frontier-economics.com/media/hgwd4e4a/the-full-cost-of-obesity-in-the-uk.pdf.

4. Sciences OfL. Life Sciences Vision, Build Back Better: our plan for growth 2021 [Available from: https://assets.publishing.service.gov.uk/media/612763b4e90e0705437230c3/life-sciences-vision-2021.pdf.

5. NHSEngland. Appendix 9 Guidance for Clinical Commissioning Groups (CCGs): Service Specification Guidance for Obesity Surgery 2016 [Available from: https://www.england.nhs.uk/wp-content/uploads/2016/05/appndx-9-serv-spec-ccg-guid.pdf.

6. NICE. Obesity: Identification, assessment and management 2014 [Available from: https://www.nice.org.uk/guidance/cg189/chapter/1-Recommendationsassessment.

7. Elhariry M, Iyer P, Isack N, Sousa B, Singh P, Abbott S, Wiggins T, Nirantharakumar K, Bellary S, Flint SW, Pournaras DJ, Hazlehurst JM. Variation in the commissioning of specialist weight management services and bariatric surgery across England: Results of a freedom of information-based mapping exercise across the 42 integrated Care Systems of England. Clin Obes. 2025 Jun;15(3):e12731. doi: 10.1111/cob.12731. Epub 2025 Jan 19. PMID: 39828897; PMCID: PMC12096057.

For peer review only

8. Hazlehurst JM, Logue J, Parretti HM, Abbott S, Brown A, Pournaras DJ, et al. Developing Integrated Clinical Pathways for the Management of Clinically Severe Adult Obesity: a Critique of NHS England Policy. Curr Obes Rep. 2020;9(4):530-43.

9. GOV.UK. New Drugs pilot to tackle obesity and cut NHS waiting list 2023 [Available from: https://www.gov.uk/government/news/new-drugs-pilot-to-tackle-obesity-and-cut-nhs-waiting-lists.

10. Desogus D, Menon V, Singhal R, Oyebode O. An Examination of Who Is Eligible and Who Is Receiving Bariatric Surgery in England: Secondary Analysis of the Health Survey for England Dataset. Obes Surg. 2019;29(10):3246-51.

11. NICE. NICE recommended weight-loss drug to be made available in specialist NHS services 2023 [Available from: https://www.nice.org.uk/news/articles/nice-recommended-weight-loss-drug-to-be-made-available-in-specialist-nhs-services.

12. NICE. Tirzepatide for managing overweight and obesity (ID6179) 2024 [Available from: https://www.nice.org.uk/guidance/indevelopment/gid-ta11156.

13. Centre DoHaSCM. Accessing Wegovy for weight loss: everything you need to know. 2023 [Available from: https://healthmedia.blog.gov.uk/2023/09/04/accessing-wegovy-for-weight-loss-everything-you-need-to-know/.

14. Seixas AA, Olaye IM, Wall SP, Dunn P. Optimizing Healthcare Through Digital Health and Wellness Solutions to Meet the Needs of Patients With Chronic Disease During the COVID-19 Era. Front Public Health. 2021;9:667654.

For peer review only

For peer review only - http://bmjopen.bmj.com/site/about/guidelines.xhtml

15. Batsis JA, Petersen CL, Clark MM, Cook SB, Kotz D, Gooding TL, et al. Feasibility and acceptability of a technology-based, rural weight management intervention in older adults with obesity. BMC Geriatr. 2021;21(1):44.

16. Huntriss R, Haines M, Jones L, Mulligan D. A service evaluation exploring the effectiveness of a locally commissioned tier 3 weight management programme offering face-to-face, telephone and digital dietetic support. Clin Obes. 2021;11(3):e12444.

17. Hanson P, Summers C, Panesar A, Oduro-Donkor D, Lange M, Menon V, et al. Low Carb Program Health App Within a Hospital-Based Obesity Setting: Observational Service Evaluation. JMIR Form Res. 2021;5(9):e29110.

18. Richards R, Wren G, Whitman M. The Potential of a Digital Weight Management Program to Support Specialist Weight Management Services in the UK National Health Service: Retrospective Analysis. JMIR Diabetes. 2024;9:e52987.

19. Senecal C, Collazo-Clavell M, Larrabee BR, de Andrade M, Lin W, Chen B, et al. A digital health weight-loss intervention in severe obesity. Digit Health. 2020;6:2055207620910279.

20. Silberman JM, Kaur M, Sletteland J, Venkatesan A. Outcomes in a digital weight management intervention with one-on-one health coaching. PLoS One. 2020;15(4):e0232221.

21. Szinay D, Forbes CC, Busse H, DeSmet A, Smit ES, König LM. Is the uptake, engagement, and effectiveness of exclusively mobile interventions for the promotion of weight-related behaviors equal for all? A systematic review. Obes Rev. 2023;24(3):e13542.

For peer review only

22. NHS. NHS Clinical Entrepreneur 2022 [Available from: https://nhscep.com/2022/07/04/our-entrepreneurs-petra-hanson/.

23. Saslow LR, Summers C, Aikens JE, Unwin DJ. Outcomes of a Digitally Delivered Low-Carbohydrate Type 2 Diabetes Self-Management Program: 1-Year Results of a Single-Arm Longitudinal Study. JMIR Diabetes. 2018;3(3):e12.

24. Abdelhameed F, Pearson E, Parsons N, Barber TM, Panesar A, Summers C, et al. Health Outcomes Following Engagement With a Digital Health Tool Among People With Prediabetes and Type 2 Diabetes: Prospective Evaluation Study. JMIR Diabetes. 2023;8:e47224.

25. NICE. Digital technologies for delivering specialist weight-management services to manage weight-management medicine: early value assessment 2023 [Available from: https://www.nice.org.uk/guidance/hte14/resources/digital-technologies-for-delivering-specialist-weightmanagement-services-to-manage-weightmanagement-medicine-early-value-assessment-pdf-50261971277509.

26. NICE. NICE real-world evidence framework 2022 [Available from: https://www.nice.org.uk/corporate/ecd9/chapter/overview.

27. Ghaferi AA, Schwartz TA, Pawlik TM. STROBE Reporting Guidelines for Observational Studies. JAMA Surgery. 2021;156(6):577.

28. Tennant PWG, Murray EJ, Arnold KF, Berrie L, Fox MP, Gadd SC, et al. Use of directed acyclic graphs (DAGs) to identify confounders in applied health research: review and recommendations. Int J Epidemiol. 2021;50(2):620-32.

For peer review only

For peer review only - http://bmjopen.bmj.com/site/about/guidelines.xhtml

29. Greenland S. An introduction to instrumental variables for epidemiologists. Int J Epidemiol. 2000;29(4):722-9.

30. Anderson DM, Wren Tracy J, Cronk R, Schram H, Behnke N, Bartram J. A toolkit for costing environmental health services in healthcare facilities. J Water Sanit Hyg Dev. 2021;11(4):668-75.

31. Boardman F. Reevaluating the remote: a realist evaluation of the practical, epistemological and ethical status of remote qualitative data collection in health. Funder MRC [Available from: https://gtr.ukri.org/projects?ref=MR%2FW021161%2F1.

32. Braun V, Clarke V. Can I use TA? Should I use TA? Should I not use TA? Comparing reflexive thematic analysis and other pattern-based qualitative analytic approaches. Counselling and Psychotherapy Research. 2021;21(1):37-47.

33. Damschroder LJ, Aron DC, Keith RE, Kirsh SR, Alexander JA, Lowery JC. Fostering implementation of health services research findings into practice: a consolidated framework for advancing implementation science. Implement Sci. 2009;4:50.

34. Hanson P, Abdelhameed F, Sahir M, Parsons N, Panesar A, de la Fosse M, Summers C, Kaura A, Randeva H, Menon V, Barber T Evaluation of the Digital Support Tool Gro Health W8Buddy as Part of Tier 3 Weight Management Service: Observational Study J Med Internet Res 2025;27:e62661URL: https://www.jmir.org/2025/1/e62661 DOI: 10.2196/62661

35. Hernández Alava M, Wailoo A, Pudney S, Gray L, Manca A. Mapping clinical outcomes to generic preference-based outcome measures: development and comparison of methods. Health Technol Assess. 2020 Jun;24(34):1-68. doi: 10.3310/hta24340. PMID: 32613941; PMCID: PMC7357250.

For peer review only - http://bmjopen.bmj.com/site/about/guidelines.xhtml

Author's contributions

Petra Hanson (chief investigator) initiated the research project, wrote the grant application and developed protocol, Amy Grove (chief investigator) initiated the research project wrote the grant application and developed protocol.

Emma Scott and Marie Thorpe (project managers) supported with writing grant application, securing funding, and developing the protocol.

Mengxi Zhang and Pranay Singh Deo (post-doctoral researchers) supported writing the protocol and are involved in conducting and analysing the research.

Manreet Thind, Keith Abrahams, Peter Auguste, Frances Griffiths, Amit Kaura, Nick Parsons and Siew Wan Hee (all co-applicants) are all involved in the methodology and analysis of the research, they also supported developing the protocol and grant applications.

Tracy Gazeley, Richard Green and Sarah O'Toole (co-applicants) supported with PPI involvement, supported with grant applications and writing the protocol.

Thomas Barber, Jonathan Hazlehurst, Akhila Mallipedhi and Anjali Zalin (co-applicants) all site principal investigators, supported with grant application and protocol.

For peer review only

Competing Interests Statement

- Dr Petra Hanson led the co-creation of W8Buddy on behalf of UHCW. She is not a stakeholder in DDM or does not have any employment contract with DDM. She is a named inventor at UHCW for W8Buddy. She has received honoraria for delivering talks from Sanofi, AstraZeneca and Royal College of Emergency Medicine.

For peer review only - http://bmjopen.bmj.com/site/about/guidelines.xhtml

- Dr Thomas M Barber has received funding for investigator-initiated research from Bayer, AstraZeneca, Shire and TDeltaS, and honoraria for educational talks and attendance at advisory board meetings for Sanofi, Novo Nordisk, Astrazeneca, Boehringer Ingelheim, MSD, Lilly and Napp.
- Prof Keith Abrams is a member of the National Institute for Health and Care Excellence (NICE) Diagnostics Advisory Committee, the NICE Decision and Technical Support Units, and is a National Institute for Health Research (NIHR) Senior Investigator Emeritus [NF-SI-0512-10159]. He has served as a paid consultant, providing unrelated methodological and strategic advice, to the pharmaceutical and life sciences industry generally, as well as to DHSC/NICE, and has received unrelated research funding from Association of the British Pharmaceutical Industry (ABPI), European Federation of Pharmaceutical Industries & Associations (EFPIA), Pfizer, Sanofi and Swiss Precision Diagnostics/Clearblue. He has also received course fees from ABPI and the University of Bristol, and is a Partner and Director of Visible Analytics Limited, a health technology assessment consultancy company.
- Arjun Panesar is the CEO and Head of AI and Ethics at DDM Health, Honorary Associate Professor at Warwick Medical School, University of Warwick, and serve as an Advisor to the Information School at the University of Sheffield, EPSRC Future Blood Testing Network, Science for Africa Foundation, NNEdPro Global Centre for Nutrition and Health in Cambridge and mentor at the Global Business School for Health at University College London. He is actively involved in the development and commercialisation of AI-based tools for digital screening, behaviour change and self-management.
- Dr Amit Kaura is the Chief Medical Officer for DDM Health but his involvement in the project is as an academic at Imperial College London. DDM Health will not be

For peer review only

For peer review only - http://bmjopen.bmj.com/site/about/guidelines.xhtml

paying Dr Kaura for his involvement, nor will they receive any project funds for his involvement.

- Dr Jonathan M. Hazlehurst declares advisory work previously for Novo Nordisk as well as honoraria for speaking engagements from Novo Nordisk and Eli Lilly and support for attendance at academic meetings from Novo Nordisk as well as Eli Lilly as well as academic funding from the NIHR.
- No other members of the research team have declared any conflicts of interest.

Patient and Public involvement (PPI)

PPI were involved in the design of the research, please refer to the methods section for further information

Funding Statement

This study/project is funded by the NIHR (grant number NIHR208100 https://fundingawards.nihr.ac.uk/award/NIHR208100). The views expressed are those of the author(s) and not necessarily those of the NIHR or the Department of Health and Social Care.

Role and responsibilities of the Sponsor

The University of Warwick will act as study sponsor. The study will be conducted in accordance with Sponsor's Standard Operating procedures. The University of Warwick has a specialist insurance policy in place which would operate in the event of any participant suffering harm as a result of their involvement in the study Zurich Municipal Insurance. NHS indemnity operates in respect of the clinical treatment that is provided.

Role and responsibilities of the Funder

For peer review only - http://bmjopen.bmj.com/site/about/guidelines.xhtml

This study is funded by the National Institute for Health and Care Research Invention for Innovation (NIHR i4i) programme (NIHR208100). Figure 4 displays the design and management of the trial are entirely independent of the funder.

Data Availability Statement

Included in supplementary material.

For peer review only

For peer review only - http://bmjopen.bmj.com/site/about/guidelines.xhtml

BMJ Open

A protocol for a cohort observational study using real-world evidence to evaluate the long-term health and economic impact of a digital tool Grohealth W8Buddy supporting access to specialist weight management services

Journal:	*BMJ Open*
Manuscript ID	bmjopen-2025-109111.R1
Article Type:	Protocol
Date Submitted by the Author:	24-Nov-2025
Complete List of Authors:	Deo, Pranay; Warwick Medical School<br>Grove, Amy; University of Birmingham<br>Zhang, Mengxi; Warwick Medical School,<br>Abrams, Keith; University of Warwick, Department of Statistics; University of York, Centre for Health Economics<br>Auguste, Peter; University of Birmingham, School of Social Policy and Society<br>Barber, Thomas; University of Warwick; University Hospitals Coventry and Warwickshire NHS Trust<br>Gazeley, Tracy; University Hospitals Coventry and Warwickshire NHS Trust<br>Green, Richard; University Hospitals Coventry and Warwickshire NHS Trust<br>Griffiths, Frances; University of Warwick, Warwick Medical School<br>Hazlehurst, Jonathan; Birmingham Health Partners; University Hospitals Birmingham NHS Foundation Trust, Department of Diabetes and Endocrinology; University of Birmingham<br>Hee, Siew Wan; University Hospitals Coventry and Warwickshire NHS Trust, Research and Development; University of Warwick Medical School<br>Kaura, Amit; Imperial College London, Faculty of medicine<br>Mallipedhi, Akhila; Hywel Dda Health Board<br>O'Toole, Sarah; University Hospitals Coventry and Warwickshire NHS Trust<br>Panesar, Arjun; DDM Health,<br>Parsons, Nicholas; Warwick Medical School, Warwick Medical School<br>Scott, Emma; University of Warwick - Coventry Campus, Warwick medical school<br>Thind, Manreet; University Hospitals Coventry and Warwickshire NHS Trust<br>Summers, Charlotte; DDM Technology House University of Warwick Coventry, UK CV4 7EZ<br>Thorpe, Marie; University of Warwick, Warwick medical school<br>Zalin, Anjali ; Barts Health NHS Trust<br>Hanson, Petra; University of Warwick, Applied Health; University Hospitals Coventry and Warwickshire NHS Trust; South Warwickshire NHS Foundation Trust

**Primary Subject Heading**:	Health services research
Secondary Subject Heading:	Evidence based practice
Keywords:	Obesity, Digital Technology, Clinical Protocols, Mobile Applications

SCHOLARONE™ Manuscripts

A protocol for a cohort observational study using real-world evidence to evaluate the long-term health and economic impact of a digital tool Grohealth W8Buddy supporting access to specialist weight management services

1. **Pranay Singh Deo** – University of Warwick Medical School Coventry CV4 4AL pranay.deo@warwick.ac.uk
2. **Amy Grove** - A.L.Grove@bham.ac.uk - University of Birmingham, Centre for Evidence and Implementation Science, Edgbaston, Birmingham B15 2TT
3. **Mengxi Zhang** - mengxi.zhang@warwick.ac.uk, University of Warwick Medical School Coventry CV4 4AL
4. **Keith Abrams** - keith.abrams@warwick.ac.uk, University of Warwick Medical School Coventry CV4 4AL
5. **Peter Auguste** - p.e.auguste.1@bham.ac.uk, University of Birmingham Health Services Management Centre, Birmingham B15 2TT
6. **Thomas M Barber** - t.barber@warwick.ac.uk, Division of Biomedical Sciences, Warwick Medical School, University of Warwick, Coventry, CV4 7AL, UK Warwickshire Institute for the Study of Diabetes, Endocrinology and Metabolism, University Hospitals Coventry and Warwickshire, Clifford Bridge Road, Coventry, CV2 2DX, UK Research Centre for Health and Life Sciences, Coventry University, Richard Crossman Building, Jordan Well, Coventry, CV1 5RW, UK
7. **Tracy Gazeley** - tracy.gazeley@uhcw.nhs.uk, University Hospitals Coventry & Warwickshire NHS trust, Clifford Bridge Road, Coventry CV2 2DX PPI
8. **Richard Green** - rmguk@hotmail.com, University Hospitals Coventry & Warwickshire NHS trust, Clifford Bridge Road, Coventry CV2 2DX PPI
9. **Frances Griffiths -** f.e.griffiths@warwick.ac.uk University Of Warwick Medical School Coventry CV4 4AL
10. **Jonathan Hazlehurst** - jonathan.hazlehurst3@uhb.nhs.uk, University Hospital Birmingham Mindelsohn Way, Birmingham B15 2GW
11. **Siew Wan Hee** - s.w.hee@warwick.ac.uk, Warwick Medical School, University of Warwick, Coventry CV4 7AL, and Research and Development, University Hospitals Coventry and Warwickshire NHS Trust, Clifford Bridge Road, Coventry CV2 2DX
12. **Amit Kaura** - a.kaura@imperial.ac.uk, Imperial College London Exhibition Road, London SW7 2AZ , Faculty of Medicine
13. **Akhila Mallipedhi** - akhila.mallipedhi@wales.nhs.uk, Hyweldda Health Board, 1 Penlan Road, Carmarthen SA31 1DS
14. **Sarah O'Toole** - sarah.otoole@uhcw.nhs.uk, University Hospitals Coventry & Warwickshire NHS trust, Clifford Bridge Road, Coventry CV2 2DX
15. **Arjun Panesar -** arjun@ddm.health, Diabetes Digital Management [DDM] Technology House Sir William Lyons Road, Coventry CV4 7EZ

For peer review only - http://bmjopen.bmj.com/site/about/guidelines.xhtml

16. **Nick Parsons** - nick.parsons@warwick.ac.uk, University Of Warwick Medical School Coventry CV4 4AL
17. **Emma Scott -** e.j.scott@warwick.ac.uk, University Of Warwick Medical School Coventry CV4 4AL
18. **Charlotte Summers** - charlotte@ddm.health, Diabetes Digital Mangement [DDM] Technology House Sir William Lyons Road, Coventry CV4 7EZ
19. **Manreet Thind** - manreet.thind@uhcw.nhs.uk, University Hospitals Coventry & Warwickshire NHS trust, Clifford Bridge Road, Coventry CV2 2DX
20. **Marie Thorpe** - marie.thorpe@warwick.ac.uk, University Of Warwick Medical School Coventry CV4 4AL
21. **Anjali Zalin** - anjali.zalin3@nhs.net, Barts Health NHS Trust, 9 Prescot Street, London E1 8PR
22. **Petra Hanson - Petra.hanson.1@warwick.ac.uk, University Of Warwick Medical School Coventry CV4 4AL ***

Corresponding Author: Petra Hanson - Petra.hanson.1@warwick.ac.uk, University Of Warwick Medical School Coventry CV4 4AL *

Trial Sponsor: University of Warwick, Coventry, CV4 7AL, 024 7652 3523

Version/24.11.2025

For peer review only

Abstract

Introduction: Obesity affects over a quarter of the UK population and can lead to serious health issues. NHS Specialist Weight Management Services offer treatments including lifestyle advice, psychological support, and medications, but access and availability vary by region. Although around 4 million people could be eligible for NHS Specialist Weight Management Services [WMS] annually, capacity is limited to 35,000, severely limiting overall access for those who need it. While digital technology has started to be used in WMS, more evidence is needed to confirm its long-term effectiveness, acceptability, and cost-effectiveness. This study explores the use of Gro Health W8Buddy, a digital platform and app providing remote Specialist WMS. It aims to determine the long-term health benefits of remote WMS pathway Gro Health W8Buddy compared to standard NHS WMS delivered in hospitals, and to improve patients access to services.

Methods and analysis: The study is a real-world evaluation with observational data collection. We will recruit 450 study participants from four NHS specialist WMS who will choose either standard NHS WMS or the digital pathway Gro Health W8Buddy. Participants are being given the option to choose their pathway to generate real world evidence. We will measure and analyse health outcomes including weight loss, time taken to be treated and cost-effectiveness, at 18 months and follow up at 24 months for later analysis [outside of this core funding]. We will gather experiential data from patients and healthcare professionals through surveys, observation, and interviews.

Ethics and dissemination: Ethical approval has been obtained from NHS Health Research Authority [HRA] and Health and Care Research Wales [HCRW] [Supplementary Figure 3] [REC reference: 25/EM/0147]. Our findings will be disseminated through academic publications, conference presentations and stakeholder engagement.

For peer review only - http://bmjopen.bmj.com/site/about/guidelines.xhtml

Strengths and limitations of this study:

- The study is structured around four distinct work packages, each employing a unique research methodology
- The digital intervention has been co-created with NHS staff and patients and is backed by research
- This research methodology has been shaped by PPI input from all four NHS sites
- The study methods enable real world evidence to be generated by allowing participants to choose their own pathway of care. Therefore, we will provide credible evidence for further roll out of digital technology in weight management services across the UK
- Allocation is not randomised which may lead to more participants in digital care or vice versa. This approach inevitably introduces complexity into the analysis, but it is a design choice advised by patient contributors. Our design means that we will not have matched cohorts across treatment arms, meaning both known confounders [e.g. age, BMI] and unknown factors may influence outcomes. To address this, our planned analyses will incorporate statistical adjustments to account for these differences as far as possible. We recognise the limitations of this approach, but it is consistent with the pragmatic design and will allow us to report findings that are representative of real-world practice.

Introduction

Background

In the UK, a quarter of adults live with obesity, with higher rates among people from Black and Asian ethnic groups and people living in the most deprived areas [1]. People living with obesity are at increased risk of chronic diseases including Type 2 Diabetes Mellitus [T2DM] and cardiovascular disease as well as at risk of shorter life expectancy [2]. Treatment of obesity cost the NHS £6.5bn in 2022, with an associated ill health annual full cost of £58bn [3]. The NHS and Office for Life Sciences [OLS] identified treatment and prevention of obesity as one of its healthcare missions [4].

A tiered approach to weight management exists in the UK. Tiers 1 and 2 are delivered in community and primary care. Tier 3 is delivered by specialist Weight Management Services [WMS] which are often based in a hospital setting. WMS comprise of multidisciplinary teams who provide dietetics, psychology, and consultation by specialist doctors. Tier 4 services include bariatric surgery [5]. In the UK, NICE recommends that people with BMI over 40 kg/m^2 or over 35 kg/m^2 with medical problems [e.g., T2DM, hypertension] are eligible for referral to WMS [6]. However, there is substantial geographical and service variation, with many areas lacking access to services [7].

Outcomes from traditional face-to-face WMS are mixed, with 50% of patients in most NHS WMS achieving a 5% weight loss over 6-12 months according to a recent review [8]. Retention rates are low [<55%] and there is little long-term [>12 months] data on sustainability of weight loss [8]. WMS have limited capacity for just 35,000 patients [9], yet 4 million people are eligible for services. This represents a significant capacity issue [10]. Due to these access constraints only 3.13% of this population receive a referral to WMS [9]. There is also marked regional variation in service design and accessibility [7]. High risk groups such as individuals from deprived areas in the UK often face barriers to accessing in

For peer review only - http://bmjopen.bmj.com/site/about/guidelines.xhtml

person Tier 3 services due to costs of transport and having support with childcare [11]. This is important to note as the obesity prevalence is 37.4% in deprived areas compared to 19.8% in less deprived areas [12].

In March 2023, NICE recommended the obesity medication semaglutide [Wegovy [TM]] for people accessing WMS [13]. Additionally, tirzpatide was recommended for obesity treatment from December 2024 [14]. These treatments represent a step change in Tier 3 WMS services and are part of the government-backed pilot to widen access to this medication with the use of digital technology [15]. However, guidance on the incorporation of digital technology to address how to safely incorporate the drugs remains unclear. We aim to fill the gap with this study.

Digital technology for managing chronic disease, including obesity, is expanding rapidly in the NHS [16]. Acceptability of digital technology for weight loss is high in both community and WMS settings, which is important to note considering the current barriers to WMS uptake and accessibility [17-20]. However, effectiveness varies. Evidence shows weight loss ranging from 13% at 4-month [digital + meal replacements] [21] to 7.6% at 12-month [digital + coaching] [22]. A community based digital weight management intervention recorded 5% weight loss at 2 years in 18% of users [20]. According to the evidence, there is a gap and need for further digital weight management research to investigate standard and digital care, alongside the integration of pharmacotherapy [e.g., semaglutide] and the use of a multi-disciplinary team [21]. Currently the evidence on the digital divide created by digital technology for weight management is lacking [23]. Therefore, innovative approaches are needed in the NHS WMS to reduce health inequality, address efficiency, increase capacity, and improve access to pharmacotherapy for obesity.

For peer review only - http://bmjopen.bmj.com/site/about/guidelines.xhtml

As a result of the above evidence, we co-created a digital support tool *Gro Health W8Buddy* [W8Buddy] with a digital company Diabetes Digital Media [DDM] to provide digital technology to deliver NHS WMS [7]. W8Buddy provides personalised WMS to support patients to achieve their self-selected health goals and support weight loss with real-time remote monitoring. The creation of W8Buddy was supported by an *HEE Topol Digital Fellowship* and *NHS Clinical Entrepreneur scheme* [24]. Evidence has shown that W8Buddy helps to reduce weight, improve glycaemic control and wellbeing among people living with obesity, overweight or with T2DM [25, 26]. W8Buddy was endorsed by *NICE Early Value Assessment* for Digital Technologies in Specialist Weight Management in October 2023 [27].

Our initial 1-year evaluation of 412 patients showed that 220 patients using W8Buddy [version 1 – information resource] *alongside* traditional WMS lost significantly more weight [0.75 kg/month] than 192 patients using traditional WMS [28]. Since then, W8Buddy has been adapted to provide a fully remote WMS. This study aims to provide longer term real-world evidence [RWE] of the clinical and cost-effectiveness of the adapted W8Buddy in the NHS.

Research question: What is the long-term [18-month] impact on clinical outcomes, including health-related quality of life [QoL] and cost-effectiveness of W8Buddy with and without weight management medication and lifestyle support to people living with obesity, compared with WMS standard care?

Objectives

1. To generate longitudinal evidence of the clinical and cost-effectiveness of the W8Buddy from four NHS WMS, University Hospital Coventry & Warwickshire [UHCW], University Hospitals Birmingham [UHB], London Luton Trust [LLT] and

For peer review only - http://bmjopen.bmj.com/site/about/guidelines.xhtml

Hywel Dda University Health Board Trust [HDUHBT], using the digital pathway W8Buddy.

2. To generate sufficient evidence to satisfy NICE guidance requirements [27].
3. To understand the process of implementation and use of W8Buddy within WMS.
4. To create a manual for safe incorporation of a digital pathway delivering weight management medication into existing WMS across the UK.
5. To make recommendations for how we can optimise existing infrastructure of WMS to improve ongoing real world evidence generation.

For peer review only

For peer review only - http://bmjopen.bmj.com/site/about/guidelines.xhtml

Methods

Study Design

Multi-site prospective comparative observational cohort study with embedded cost-effectiveness analysis and process evaluation. Comprising of four work packages [WP] to assess the following; 1] Set up, Patient and Public Involvement and Engagement [PPIE] & Stakeholder engagement, 2] Clinical impact, 3] Health economic impact and 4] Process evaluation.

Eligibility criteria to enter WMS in the UK

Inclusion criteria reflect the eligibility of people to enter the clinical services participating in this research, including services with additional eligibility criteria restrictions [subject to local processes that may differ slightly]. However, whilst individual services may have further restrictive criteria, which is reflective of many WMS all participants are included within:

- Adults aged 18 years and above
- Class 2 obesity [BMI $\geq$ 35kg/m^2] with at least 1 medical complication [e.g., prediabetes, type 2 diabetes [T2DM]]; or patients with obesity class 3 [BMI $\geq$ 40kg/m^2], without any complications.

The eligibility criteria for access to Tier 3 WMS for adults from South Asian, Chinese, other Asian, Middle Eastern, Black African, or African-Caribbean background have BMI cut offs 37.5 kg/m^2 without comorbidities.

Exclusion criteria:

For peer review only - http://bmjopen.bmj.com/site/about/guidelines.xhtml

- Pregnant or breastfeeding
- Type 1 diabetes mellitus
- End stage renal disease [eGFR<15, receiving renal replacement therapy]
- Active or untreated alcohol or drug dependency
- Decompensated liver disease
- History of anorexia, undergoing treatment or awaiting treatment for DSM-V classified eating disorder
- Severe mental health disease, including acute mental health crisis or self-harm in the last 12 months
- Unstable mental health disease including unstable personality disorder, schizophrenia, and bipolar disorder
- Unwilling or unable to commit to participation across the length of the research study.
- Patients targeting bariatric surgery within the length of the research study.
- Participating in another weight management research intervention
- Participating in the diabetes remission program

Study setting and sites

This study will run at four NHS sites [UHCW, UHB, LLT, HDUHBT]. Standard care differs across the recruiting sites, including the mixed provision of obesity medication. Sites without medication as standard care will be noted as the digital pathway *without medication.*

For peer review only - http://bmjopen.bmj.com/site/about/guidelines.xhtml

For peer review only

Patient and public involvement [PPI]

A W8Buddy PPI Group has been established with a minimum of four PPI Representatives with lived experience of WMS from each site. Potential PPI representatives have been identified through WMS teams at each participating site and obesity charities. Those interested in joining the groups are invited to meet with the PPIE Lead and/or UHCW PPIE Team to discuss the opportunity, commitment required, and support offered and to find out about their experience and background. PPI representatives selected represent the diverse population of the WMS users in each area. The Group meet quarterly to ensure ongoing input from people with lived experience, including identifying outcome measures that are meaningful for people with obesity, providing feedback on participant facing documents, working on creating local pathways and training materials. The PPI Group will also shape analysis and risk mitigation, may take part in conducting semi-structured interviews as part of qualitative research, and support dissemination of findings afterwards.

Sample size calculation

We will collect weight data at baseline and a minimum of three occasions during the study, for both pathways. Powering the study to show that the digital pathway is non-inferior to the standard of care pathway, with a difference less than 4kg, at 90% power and a two-sided 5% level, gives a sample size of approximately 180 participants. Further assuming a correlation between the repeated weight measurements in the range 0.2 to 0.5, which previously collected data suggests is reasonable [29], based on a longitudinal mixed model/repeated measures ANCOVA gives a total sample size of 360. Conservatively assuming 20% loss during follow-up, the target sample size is 450.

Given the current widespread use of the digital [W8Buddy] intervention at the lead centre [UHCW], the control [standard of care; SC] intervention at this site will be different from that

For peer review only - http://bmjopen.bmj.com/site/about/guidelines.xhtml

offered at the other sites. For this reason, we plan to characterise the control intervention at UHCW as a “hybrid” digital intervention. We will aim to recruit sufficient participants [i.e. 360] at the three other sites [UHB, LLT, HDUHB] to fully power the study for the planned comparison between the digital and standard of care groups. Additionally, we will collect data from the “hybrid” digital intervention at UHCW to augment and further refine the primary analysis.

Screening, recruitment, and consent

Potential participants will be identified by research nurses once they have been referred [or self-referred] to the local Tier 3 WMS. Participants will be screened electronically against pre-specified criteria.

Eligible participants will be able to choose how they receive their WMS - either in the standard NHS delivery, or via fully digital platform Gro Health W8Buddy. Site specific standard of care pathways will be delivered by the usual health care professionals, employed by each NHS site. The digital pathway will be delivered by health care professionals employed by DDM [providers of Gro Health W8Buddy]. We aim to recruit a total of 450 participants across both arms from September 2025 to February 2026.

Those who satisfy the study inclusion criteria will be contacted by the research team via telephone. Brief information about the study will be provided to potential participants, including a link to a website with information about the study. This website will be password protected as only eligible participants will be able to view it. Digital information sheets and consent forms will be hosted on the website.

For peer review only

If a patient is unable to access the website [therefore, unable to complete electronic consent] the local site research nurse will invite them to the hospital. They will show the participant the research website and help them with electronic consent. Later, the research nurse will print out the consent form [signed copy] only for the patient if they do not have access to email.

Electronic consent for data collection, with an option to consent for ongoing data collection, will be obtained. Following informed consent, participants will be able to choose the digital or standard care pathway via the website. A copy of the signed consent form will be sent to the participants, local research team and site Principal Investigator [PI].

To support data collection in the standard care pathway, participants will be offered a £10 voucher for each completed survey at month 0, 6, 12, 18 and 24; vouchers will not be provided for sign up only, surveys must be completed by participants.

Intervention Gro Health W8Buddy

Gro Health W8Buddy is a virtual specialist WMS pathway. It is accessed through a digital behavioural change platform called the Gro Health app [see, Figure 1]. Participants on the W8Buddy digital arm will receive 52 weeks of support from health care professionals employed by DDM. At week 52 each participant on the digital pathway will be assessed and the following options offered: discharge to primary care, onward referral to local Tier 4 if interested in surgery, onward referral for local Tier 3 MDT discussion, extension of digital pathway by 6-months, referral to local Tier 2 weight management service.

Figure 1: Gro Health W8Buddy platform and app – description and visual representation of the W8Buddy platform in an app format

For peer review only - http://bmjopen.bmj.com/site/about/guidelines.xhtml

Standard care

Participants in the standard care arm will receive the Tier 3 WMS that is delivered by their local NHS trust. All four NHS trusts included in the research have differences in their Tier 3 service, which will be explored in detail as part of the study. The differences range from the amount of input from healthcare professionals [psychologist, dietitian, physician], modality of sessions [1-1 or group sessions] and frequency. Post study care for participants in standard care will be delivered in accordance to their NHS trust policies for Tier 3 weight management. As discussed previously, the W8Buddy intervention is included in the standard of care at the lead centre [UHCW]. It is used as a digital support tool only [no access to clinicians via W8Buddy], enhancing the usual face to face care, and as such, differs from entirely remote digital pathway. For this reason, the model of delivery of care is hybrid at UHCW [refer to table 1 for further detail].

Table 1. Standard of care

NHS Site	Standard of care	Weight management medication offered in current tier 3 service* *please note this information is accurate at time of publication*
UHCW	Hybrid standard of care with digital W8Buddy input and in person MDT support. Mixture of one-to-one and group sessions delivered by dietitians, psychologists, and consultant endocrinologists.	No
UHB	Mixture of group sessions and one-to-one appointments delivered by dietitians, psychologists and consultant endocrinologists following a face-to-face introductory group session.	No

For peer review only - http://bmjopen.bmj.com/site/about/guidelines.xhtml

LLT	Mixture of face to face and group sessions delivered by psychologists, dietitians and consultant endocrinologist.	No
HDUHBT	Mixture of face to face and group sessions delivered by psychologists and dietitians. After completion they are seen by consultant.	Yes

Figure 2: Participant flow chart, the flow chart describes where participants will be providing data in both the digital and standard of care arm.

Assessment and management of risk

Risks relate to data management/reporting across four sites and management of the digital pathway delivered by DDM. The latter is assured by DDM. Table 2 lists these. Further information on data management and flow can be found in supplementary material table 1 (ST1), adverse events (ST2) and supplementary figure 1 (SF1).

Table 2 Assessment and management of risk

Data reporting	Not reporting data by participants, mitigated by regular support via the app or contact from research team if not completing surveys.
Data protection	As with any online service proper data handling is assured by high security data storage in line with NHS Digital policies. Data from NHS sites transferred to analytical partner using secure pathways.
Delivery	The site research team will oversee all participants, including those accessing digital pathway. The first contact for any queries for participants on digital pathway will be the digital provider, and if the query is not resolved the research site team will be contacted. The project will be managed by PH and AG via weekly WP meetings between sites and project manager, with monthly oversight by each site PI. Three-monthly meetings of PPIE group will ensure ongoing input from patients. Annual project steering committee meetings will review risk registers.
Imbalanced recruitment	More patients opting for digital rather than standard of care pathway [or vice-versa], mitigated by over-recruiting standard of care [or digital] patients and adjusting as per analysis plan using propensity-weighted approach, purposive sampling and ongoing monitoring of recruitment, communication to sites, telephone and offline packs as another option for those choosing digital pathway.

For peer review only - http://bmjopen.bmj.com/site/about/guidelines.xhtml

Engagement with the digital pathway	Low engagement from high-risk/target groups resulting in lower enrolments and participation from those most in need of the service. Mitigated by patient friendly resources, onboarding support, research nurse at each site to act as a point of call.
Patient safety	Without face-to-face contact, healthcare professionals may miss important physical signs and symptoms that might indicate a health concern for study participants. Participants might also misunderstand or misuse instructions regarding their medication, increasing the risk of side effects or adverse events. This is mitigated through symptom tracking, face-to-face videoconferencing/virtual calls, identifying signs of body language, and recording sessions for audit to ensure ongoing patient safety. Digital phenotyping of patients occurs in real-time, and any thresholds of anxiety or depression are noted in the clinical dashboard and the clinical team are notified. Omni-directional communication through the platform allows clinicians to engage directly with patients and vice-versa with up-to-date, real-time data. Patient expectations are managed by informing the patient if their coach is offline and encouraging them to seek emergency medical assistance if at risk.
Medication safety on digital pathway	Prescribing medication remotely requires a comprehensive understanding of the patient's current health status, medications, and lifestyle, which can be challenging to obtain in a virtual environment. There is also the risk of potential drug interactions that might be overlooked without a detailed in-person evaluation. This is mitigated through patient completion of psychological assessments, medication eligibility assessment, and approval for medication from the multidisciplinary team [MDT] team providing the patient's care. Regular reviews ensure medication is not mismanaged and appropriate for the patient. Patient is provided with videos and resources on how to inject.
Psychological safety on digital pathway	Weight management is closely tied to self-esteem and body image, which can be negatively impacted if the focus is solely on weight loss. Additionally, the digital nature of virtual services can exacerbate feelings of isolation and lack of support. This is mitigated through support from a psychotherapist/psychologist throughout the programme, access to private/group coaching for on-demand support, and artificial intelligence [AI] algorithms that provide digital phenotype measurements for a patient's likelihood of anxiety, stress, and depression based on conversations/engagements in the community.
Eating disorders	Weight loss can trigger or exacerbate eating disorders. For individuals with a history of eating disorders, weight loss medications could potentially be misused in a harmful way. This is mitigated through patient completion of psychological assessments, medication eligibility

For peer review only - http://bmjopen.bmj.com/site/about/guidelines.xhtml

	assessment, and approval for medication from the MDT team providing the patient's care. On the digital pathway, regular reviews with a pharmacist and psychologist ensure medication is not mismanaged and appropriate for the patient. Signposting and referral back to the GP will be provided to any patients who display signs of disordered eating. On the standard of care pathway, local protocols will be followed.
Engagement with the standard of care pathway	High drop out from the standard of care pathway: there is a high drop out from the standard of care service in Tier 3 Weight Management, resulting in high loss to follow up. This will be mitigated by having a dedicated research nurse who will contact patients to ensure they complete all data collection in timely fashion. Additionally, participants in the standard of care will receive vouchers to compensate them for the additional surveys they will be asked to do.

Outcomes

The primary outcome is changes in body weight after 18-months of using either [i] a fully digital weight management programme [remote digital pathway Gro Health W8Buddy] or [ii] site-specific delivered weight management programmes incorporating face to face and/or virtual group sessions [standard care].

Secondary outcomes are metabolic markers [HBA1c, lipids], blood pressure, waist circumference and health related quality of life [EQ-5D-5L] [30].

For peer review only

For peer review only - http://bmjopen.bmj.com/site/about/guidelines.xhtml

Data collection

In Supplementary Table 4 (ST4) we detail what baseline and follow up data will be collected to assess primary and secondary outcomes. During the economic evaluation, additional cost, and process evaluation qualitative data will be collected.

For peer review only

For peer review only - http://bmjopen.bmj.com/site/about/guidelines.xhtml

Monitoring

The study will be monitored by the study management group and the University of Warwick representative of the Sponsor to ensure that the study is being conducted as per protocol, adhering to Research Governance and Good Clinical Practice [GCP]. Central monitoring activities will be performed such as data quality checks. The recruiting site is obliged to assist the sponsor in monitoring the study. These may include hosting site visits, providing information for remote monitoring, or putting procedures in place to monitor the study internally. The approach to, and extent of, monitoring will be specified in a study monitoring plan determined by the risk assessment undertaken prior to the start of the study.

Analysis

Statistical analysis plan A detailed statistical analysis plan will be prepared by the statisticians, and agreed with the study team, prior to final analysis, and which will follow the NICE RWE Framework [28]. Comparisons will be primarily on an as-started [intention to treat, ITT] basis. We do not expect any or only minor levels of treatment switching or discontinuation for the W8Buddy group. Therefore, we would expect the on-treatment and as-started treatment effect estimates to be equivalent in this population. We will report the result according to the STROBE guideline [29].

Summary of baseline data Demographic and baseline data will be summarised overall and by treatment arms and sites. Categorical data will be summarised as count and percentage, and continuous data will be summarised as number of missing data, mean with standard deviation, and median with 25th and 75th percentiles.

For peer review only

Missing data Patterns of missing data/discontinuation will be reported and assessed if missingness was at random or provided useful information on treatment efficacy.

Primary and secondary outcome analysis The primary analysis will compare temporal changes in weight for the W8Buddy and standard care group, from baseline to completion of follow-up [at 18 months], using longitudinal mixed effects regression models. The effectiveness of the W8Buddy will be quantified by differences in rates of weight change compared to standard of care during follow-up. All the secondary outcomes will be analysed individually with longitudinal mixed effects models.

For all analyses, we will consider adjusting for a range of demographics and clinical characteristics [e.g. age, gender, ethnicity, Index of Multiple Deprivation/Welsh Index of Multiple Deprivation, comorbidities, NHS site, mode and distance of travel and other confounders identified using Directed Acyclic Graphs with stakeholders PPI members and members of the steering committee] [33] using propensity-weighted longitudinal mixed models. We expect the fixed effects to be demographics and clinical characteristics as mentioned above and the random effects to be the sites and individuals. A sensitivity analysis using *distance to NHS site* as an instrumental variable [34] will also be conducted to assess the assumption of no unmeasured confounders.

The comparison between the hybrid intervention and both the standard of care and fully digital intervention will be an unpowered secondary analysis, that we hope will offer additional insights and help to characterise the magnitude of intervention effects from the primary analysis.

Economic evaluation

For peer review only - http://bmjopen.bmj.com/site/about/guidelines.xhtml

We will develop a costing toolkit to provide transparent resource use and costs for the delivery of the standard care and digital delivery pathways. The toolkit development process will comprise three stages: planning, data collection, synthesis and analysis [35, 36]. We will require resource use and cost estimates for delivery of W8Buddy providing weight management medication and standard of care [Tier 3 WMS]. We will produce a systematic review of cost effectiveness literature to inform the development of a decision-analytic model. The model will be used to estimate the cost-effectiveness of Tier 3 WMS in addition to W8Buddy compared to weight management services delivering weight management medication to support people with obesity. This will be fundamental to help to understand the long-term cost-effectiveness of weight management programmes as they allow for the extrapolation of the short-term benefits of interventions.

Long-term follow-up analysis Outcomes collected beyond the 18-month primary outcome will be analysed at a later stage, subject to further funding. The aim of the longer term follow-up is to explore the long-term impact of digital weight management interventions. We expect the analyses will be done similarly to our primary and secondary clinical, and health economic analyses.

Process Evaluation

A mixed methods process evaluation will be conducted to understand the implementation of W8Buddy. Process evaluation data is listed in Supplementary Table 4.

Electronic data from intervention and control pathways: We will collate data from the electronic clinical records, intervention, and study records of all study participants data on:

For peer review only - http://bmjopen.bmj.com/site/about/guidelines.xhtml

- Fidelity: delivery of coaching sessions, check-ins and physical activity sessions and progress reviews
- Adherence: Including biomedical explanations of adherence [comorbidities], we will assess user adherence to each consultation in the digital pathway [a patient attending 75% of consultations or more is considered adherent]
- Who chooses the digital pathway or not
- Who engages with the digital pathway, their level of engagement and predictors of engagement

Qualitative data collection and analysis

We will record observation data as field notes and collate documents. Qualitative data will be obtained from participants interviews via telephone or videoconference. We will audio record the interviews, transcribe using software and check the transcripts against the audio recording. We will undertake thematic analysis, with independent quality checks. [29]. This analysis will be undertaken independently of the quantitative analysis team.

Initial implementation and health professional experience of delivering digital pathway

We will observe site team meetings and implementation of the intervention, access team documents and undertake interviews with team members implementing the digital pathway to understand how they are meeting implementation challenges, training delivered, and quality assurance processes put in place. During the observations, the patient will be asked for permission for the researcher to remain present during the health professional-patient interaction. If this is refused, the researcher will leave.

For peer review only - http://bmjopen.bmj.com/site/about/guidelines.xhtml

In months 12 and 18 we will interview those delivering the digital pathway to understand their experiences, how they adapt WMS and any unexpected pathways or consequences [estimate 5/site]. We will use implementation frameworks [37] to guide thematic analysis of implementation data whilst remaining alert to new themes. Early analysis of data on implementation will be used to provide formative feedback for subsequent intervention site teams.

User experience of digital pathway

In semi-structured interviews with users, we will explore their experiences of engaging with the digital pathway, influences on their engagement, challenges and how these were met, adaptations, impact, and mechanisms of impact [estimate 10/site, total 40].

We will undertake brief structured interviews with participants not choosing the digital pathway [5/site] and participants not adherent to the digital pathway and/or medicine [5/site]. We will ask participants why they did not choose the digital pathway/drop out or return to the digital pathway/medicine. We will develop our brief structured questions with input from the study PPI group to ensure neutral, non-judgemental phrasing. We will sample participants consecutively across study sites and approach them for recruitment at least a week after their decision.

Describing the control intervention patient pathway

A researcher will visit each site to observe the control intervention. The team providing the control intervention will provide a written description of the control intervention patient pathway. From observation and conversation, the researcher will add additional details and variation on that pathway. The study team will analyse the observation notes to compile a description of the control intervention and check this with staff delivering the control

For peer review only - http://bmjopen.bmj.com/site/about/guidelines.xhtml

intervention. Study sites will extract from the appointment system and relevant clinical records of their control intervention pathway, patient adherence data in terms of attendance at appointments, whether they are individual or group and duration of appointments, and use of medication.

Ethics and dissemination Ethical approval for this study has been obtained from the Health Research Authority [HRA] Research Ethics Committee [REC] and Health and Care Research Wales [HCRW] [REC reference: 25/EM/0147][Supplementary Figure 3]. Informed consent will be required to enter the study, as well as to share anonymised data with local health care team and study team. Participants will be consented for long term follow-up using routinely collected data. Protocol amendments [if any] will be reported and discussed with National Institute for Health and Care Research [NIHR] and local ethics committee.

Study data will be collected on the screening logs, W8Buddy App, and validated questionnaires [see Supplementary Table 4] electronically. Health record data for each site will be accessed by local research team. An Electronic Data Capture system will be used to record and store study data transferred securely from the W8Buddy App and hospital medical records. This will be the main Study Database. Only research team and members of the DDM team will have access to it. They will be listed in the delegation log and must hold Good Clinical Practice [GCP] certification. Audio recordings of interviews will be stored on secure University of Warwick computers and accessed only by research team located at Warwick.

We have a tiered approach to dissemination to ensure our findings reach participants, policy makers and practitioners. We will host a stakeholder engagement dissemination event at the end of the study to maximise the reach of our finding.

For peer review only

Our results will inform UK guidelines [incorporation of digital tool delivering pharmacotherapy into obesity management guideline]. Academic publications will include protocol paper, main results of RWE evaluation, the health economic assessment and a paper describing the scale-up in NHS services and impact on multiple long-term conditions. We will create NHS business case, build on ongoing partnership developed across four adoption sites, produce equality and health inequality impact assessment, and have evidence for health and financial impact to support NHS adoption. We will improve NHS services by facilitating the testing and scale-up of our digital innovation which can support other Trusts wanting to deliver digitally enabled services. This will include recommendations to other service providers, targeted education, and creation of a virtual learning environment [hosted by an establish Warwick University platform]. Our study outputs will provide evidence to apply for a NICE review of clinical guidelines and an update to NHS reimbursement criteria, which will open the door to national adoption by weight management services.

For peer review only

Discussion

Research is needed to determine how we can most effectively deliver WMS to more people [21]. One potential solution to address issues of patient access is the introduction of digital services [14-18]. However, long term evidence about clinical and cost-effectiveness is

For peer review only - http://bmjopen.bmj.com/site/about/guidelines.xhtml

required before digital pathways are fully integrated into NHS WMS across the whole country.

In this study we aim to provide longer term real-world evidence [RWE] of the clinical and cost-effectiveness, as well as barriers and facilitators of uptake of W8Buddy digital pathway implemented in four NHS sites. All these potential findings are essential considering the current data on the demand for WMS, as well as the health and economic impact of obesity [2,3]. Guidance on the incorporation of weight management drugs alongside digital interventions is also an unclear gap we intend to fill with our research. With capturing the variation that exists amongst our four NHS sites, we will provide guidance on implementation into further NHS sites.

Allowing study participants to choose their own pathway will allow RWE to understand uptake of the digital pathway when incorporated into standard care. Furthermore, we are assessing whether W8Buddy can decrease NHS waiting times and costs. Individuals living with obesity could in time be supported virtually without the need to attend in person specialist services with the associated costs and challenges of access. Investigating pathways, waiting times and accessibility is important for Obesity research considering the low retention rates and high demand but low availability in WMS [7,11,12]. The use of a digital pathway will also provide further insight for innovative diagnostic pathways for obesity management, such as moving from acute to home care and being offered to more patients in a cost-effective way, which would further contribute to the literature base.

We acknowledge the challenges that may occur with the adoption of a digital pathway into NHS sites such as recruitment rates, levels of engagement and differences in WMS at NHS sites. However, capturing any challenges that may arise and providing solutions will benefit the future implementation of W8Buddy and contribute to generalisable knowledge.

For peer review only - http://bmjopen.bmj.com/site/about/guidelines.xhtml

We aim to improve NHS Specialist WMS services by facilitating the evidence generation and scale-up of digital innovation Gro Health W8Buddy. This could in turn support other NHS organisations wanting to deliver digitally enabled services. The findings of this study will be used to generate recommendations for other service providers, targeted education, and creation of a virtual learning environment. It is aimed the study outputs will provide evidence needed for full NICE technology appraisal, as well as evidence for NHS commissioners to facilitate national adoption of technology in specialist WMS.

For peer review only

For peer review only - http://bmjopen.bmj.com/site/about/guidelines.xhtml

References

1. Baker C. Obesity statistics 2023 [Available from: https://researchbriefings.files.parliament.uk/documents/SN03336/SN03336.pdf.
2. Peeters A, Barendregt JJ, Willekens F, Mackenbach JP, Al Mamun A, Bonneux L. Obesity in adulthood and its consequences for life expectancy: a life-table analysis. Ann Intern Med. 2003;138[1]:24-32.
3. Economics F. Estimating the full costs of obesity 2022 [Available from: https://www.frontier-economics.com/media/hgwd4e4a/the-full-cost-of-obesity-in-the-uk.pdf.
4. Sciences OfL. Life Sciences Vision, Build Back Better: our plan for growth 2021 [Availablefrom:https://assets.publishing.service.gov.uk/media/612763b4e90e0705437230c3/life-sciences-vision-2021.pdf]
5. NHSEngland. Appendix 9 Guidance for Clinical Commissioning Groups [CCGs]: Service Specification Guidance for Obesity Surgery 2016 [Available from: https://www.england.nhs.uk/wp-content/uploads/2016/05/appndx-9-serv-spec-ccg-guid.pdf.
6. NICE. Obesity: Identification, assessment and management 2014 [Available from: https://www.nice.org.uk/guidance/cg189/chapter/1-Recommendationsassessment.
7. Elhariry M, Iyer P, Isack N, Sousa B, Singh P, Abbott S, Wiggins T, Nirantharakumar K, Bellary S, Flint SW, Pournaras DJ, Hazlehurst JM. Variation in the commissioning of specialist weight management services and bariatric surgery across England: Results of a freedom of information-based mapping exercise across the 42 integrated

For peer review only

Care Systems of England. Clin Obes. 2025 Jun;15[3]:e12731. doi: 10.1111/cob.12731. Epub 2025 Jan 19. PMID: 39828897; PMCID: PMC12096057.

8. Hazlehurst JM, Logue J, Parretti HM, Abbott S, Brown A, Pournaras DJ, et al. Developing Integrated Clinical Pathways for the Management of Clinically Severe Adult Obesity: a Critique of NHS England Policy. Curr Obes Rep. 2020;9[4]:530-43.
9. GOV.UK. New Drugs pilot to tackle obesity and cut NHS waiting list 2023 [Available from: https://www.gov.uk/government/news/new-drugs-pilot-to-tackle-obesity-and-cut-nhs-waiting-lists.
10. Desogus D, Menon V, Singhal R, Oyebode O. An Examination of Who Is Eligible and Who Is Receiving Bariatric Surgery in England: Secondary Analysis of the Health Survey for England Dataset. Obes Surg. 2019;29[10]:3246-51.
11. Office for Health Improvement and Disparities. Obesity Profile: Short Statistical Commentary – May 2025 [Available from:https://www.gov.uk/government/statistics/obesity-profile-may-2025-update/obesity-profile-short-statistical-commentary-may-2025].
12. Public Health England. Weight Management Services: National Mapping Exercise Report 2017[https://assets.publishing.service.gov.uk/media/5a7f74d2e5274a2e8ab4c4b1/Final_Weight_Management_Mapping_Report.pdf].
13. NICE. NICE recommended weight-loss drug to be made available in specialist NHS services 2023 [Available from: https://www.nice.org.uk/news/articles/nice-recommended-weight-loss-drug-to-be-made-available-in-specialist-nhs-services.
14. NICE. Tirzepatide for managing overweight and obesity [ID6179] 2024 [Available from: https://www.nice.org.uk/guidance/indevelopment/gid-ta11156.

For peer review only - http://bmjopen.bmj.com/site/about/guidelines.xhtml

15. Centre DoHaSCM. Accessing Wegovy for weight loss: everything you need to know. 2023 [Available from: https://healthmedia.blog.gov.uk/2023/09/04/accessing-wegovy-for-weight-loss-everything-you-need-to-know/.

16. Seixas AA, Olaye IM, Wall SP, Dunn P. Optimizing Healthcare Through Digital Health and Wellness Solutions to Meet the Needs of Patients With Chronic Disease During the COVID-19 Era. Front Public Health. 2021;9:667654.

17. Batsis JA, Petersen CL, Clark MM, Cook SB, Kotz D, Gooding TL, et al. Feasibility and acceptability of a technology-based, rural weight management intervention in older adults with obesity. BMC Geriatr. 2021;21[1]:44.

18. Huntriss R, Haines M, Jones L, Mulligan D. A service evaluation exploring the effectiveness of a locally commissioned tier 3 weight management programme offering face-to-face, telephone and digital dietetic support. Clin Obes. 2021;11[3]:e12444.

19. Hanson P, Summers C, Panesar A, Oduro-Donkor D, Lange M, Menon V, et al. Low Carb Program Health App Within a Hospital-Based Obesity Setting: Observational Service Evaluation. JMIR Form Res. 2021;5[9]:e29110.

20. Richards R, Wren G, Whitman M. The Potential of a Digital Weight Management Program to Support Specialist Weight Management Services in the UK National Health Service: Retrospective Analysis. JMIR Diabetes. 2024;9:e52987.

21. Senecal C, Collazo-Clavell M, Larrabee BR, de Andrade M, Lin W, Chen B, et al. A digital health weight-loss intervention in severe obesity. Digit Health. 2020;6:2055207620910279.

22. Silberman JM, Kaur M, Sletteland J, Venkatesan A. Outcomes in a digital weight management intervention with one-on-one health coaching. PLoS One. 2020;15[4]:e0232221.

For peer review only

For peer review only - http://bmjopen.bmj.com/site/about/guidelines.xhtml

23. Szinay D, Forbes CC, Busse H, DeSmet A, Smit ES, König LM. Is the uptake, engagement, and effectiveness of exclusively mobile interventions for the promotion of weight-related behaviors equal for all? A systematic review. Obes Rev. 2023;24[3]:e13542.
24. NHS. NHS Clinical Entrepreneur 2022 [Available from: https://nhscep.com/2022/07/04/our-entrepreneurs-petra-hanson/.
25. Saslow LR, Summers C, Aikens JE, Unwin DJ. Outcomes of a Digitally Delivered Low-Carbohydrate Type 2 Diabetes Self-Management Program: 1-Year Results of a Single-Arm Longitudinal Study. JMIR Diabetes. 2018;3[3]:e12.
26. Abdelhameed F, Pearson E, Parsons N, Barber TM, Panesar A, Summers C, et al. Health Outcomes Following Engagement With a Digital Health Tool Among People With Prediabetes and Type 2 Diabetes: Prospective Evaluation Study. JMIR Diabetes. 2023;8:e47224.
27. NICE. Digital technologies for delivering specialist weight-management services to manage weight-management medicine: early value assessment 2023 [Available from: https://www.nice.org.uk/guidance/hte14/resources/digital-technologies-for-delivering-specialist-weightmanagement-services-to-manage-weightmanagement-medicine-early-value-assessment-pdf-50261971277509.
28. NICE. NICE real-world evidence framework 2022 [Available from: https://www.nice.org.uk/corporate/ecd9/chapter/overview.
29. Ghaferi AA, Schwartz TA, Pawlik TM. STROBE Reporting Guidelines for Observational Studies. JAMA Surgery. 2021;156[6]:577.
30. Hanson P, Abdelhameed F, Sahir M, Parsons N, Panesar A, de la Fosse M, Summers C, Kaura A, Randeva H, Menon V, Barber T Evaluation of the Digital Support Tool Gro Health W8Buddy as Part of Tier 3 Weight Management Service: Observational

For peer review only

For peer review only - http://bmjopen.bmj.com/site/about/guidelines.xhtml

Study J Med Internet Res 2025;27:e62661URL: https://www.jmir.org/2025/1/e62661 DOI: 10.2196/62661

31. Braun V, Clarke V. Can I use TA? Should I use TA? Should I not use TA? Comparing reflexive thematic analysis and other pattern-based qualitative analytic approaches. Counselling and Psychotherapy Research. 2021;21[1]:37-47.
32. Hernández Alava M, Wailoo A, Pudney S, Gray L, Manca A. Mapping clinical outcomes to generic preference-based outcome measures: development and comparison of methods. Health Technol Assess. 2020 Jun;24[34]:1-68. doi: 10.3310/hta24340. PMID: 32613941; PMCID: PMC7357250.
33. Tennant PWG, Murray EJ, Arnold KF, Berrie L, Fox MP, Gadd SC, et al. Use of directed acyclic graphs [DAGs] to identify confounders in applied health research: review and recommendations. Int J Epidemiol. 2021;50[2]:620-32.
34. Greenland S. An introduction to instrumental variables for epidemiologists. Int J Epidemiol. 2000;29[4]:722-9.
35. Anderson DM, Wren Tracy J, Cronk R, Schram H, Behnke N, Bartram J. A toolkit for costing environmental health services in healthcare facilities. J Water Sanit Hyg Dev. 2021;11[4]:668-75.
36. Boardman F. Reevaluating the remote: a realist evaluation of the practical, epistemological and ethical status of remote qualitative data collection in health. Funder MRC [Available from: https://gtr.ukri.org/projects?ref=MR%2FW021161%2F1.
37. Damschroder LJ, Aron DC, Keith RE, Kirsh SR, Alexander JA, Lowery JC. Fostering implementation of health services research findings into practice: a consolidated framework for advancing implementation science. Implement Sci. 2009;4:50.

For peer review only - http://bmjopen.bmj.com/site/about/guidelines.xhtml

Figure 1: Gro Health W8Buddy platform and app – description and visual representation of the W8Buddy platform in an app format

Figure 2: Participant flow chart, the flow chart describes where participants will be providing data in both the digital and standard of care arm.

For peer review only

For peer review only - http://bmjopen.bmj.com/site/about/guidelines.xhtml

Author's contributions

Petra Hanson [chief investigator and guarantor] initiated the research project, wrote the grant application and developed protocol, Amy Grove [chief investigator] initiated the research project wrote the grant application and developed protocol.

Emma Scott and Marie Thorpe [project managers] supported with writing grant application, securing funding, and developing the protocol.

Pranay Singh Deo [author] and Menxi Zhang [post-doctoral researchers] supported writing the protocol and are involved in conducting and analysing the research.

Manreet Thind, Keith Abrahams, Peter Auguste, Frances Griffiths, Amit Kaura, Nick Parsons and Siew Wan Hee [all co-applicants] are all involved in the methodology and analysis of the research, they also supported developing the protocol and grant applications.

Tracy Gazeley, Richard Green and Sarah O'Toole [co-applicants] supported with PPI involvement, supported with grant applications and writing the protocol.

Thomas Barber, Jonathan Hazlehurst, Akhila Mallipedhi and Anjali Zalin [co-applicants] all site principal investigators, supported with grant application and protocol.

Arjun Panesar and Charlotte Summers [co-applicants] support with developing the protocol and producing the W8Buddy digital app.

Competing Interests Statement

- Dr Petra Hanson led the co-creation of W8Buddy on behalf of UHCW. She is not a stakeholder in DDM or does not have any employment contract with DDM. She is a named inventor at UHCW for W8Buddy. She has received honoraria for delivering talks from Sanofi, AstraZeneca and Royal College of Emergency Medicine.

For peer review only

- Dr Thomas M Barber has received funding for investigator-initiated research from Bayer, AstraZeneca, Shire and TDeltaS, and honoraria for educational talks and attendance at advisory board meetings for Sanofi, Novo Nordisk, Astrazeneca, Boehringer Ingelheim, MSD, Lilly and Napp.
- Prof Keith Abrams is a member of the National Institute for Health and Care Excellence [NICE] Diagnostics Advisory Committee, the NICE Decision and Technical Support Units, and is a National Institute for Health Research [NIHR] Senior Investigator Emeritus [NF-SI-0512-10159]. He has served as a paid consultant, providing unrelated methodological and strategic advice, to the pharmaceutical and life sciences industry generally, as well as to DHSC/NICE, and has received unrelated research funding from Association of the British Pharmaceutical Industry [ABPI], European Federation of Pharmaceutical Industries & Associations [EFPIA], Pfizer, Sanofi and Swiss Precision Diagnostics/Clearblue. He has also received course fees from ABPI and the University of Bristol, and is a Partner and Director of Visible Analytics Limited, a health technology assessment consultancy company.
- Arjun Panesar is the CEO and Head of AI and Ethics at DDM Health, Honorary Associate Professor at Warwick Medical School, University of Warwick, and serve as an Advisor to the Information School at the University of Sheffield, EPSRC Future Blood Testing Network, Science for Africa Foundation, NNEdPro Global Centre for Nutrition and Health in Cambridge and mentor at the Global Business School for Health at University College London. He is actively involved in the development and commercialisation of AI-based tools for digital screening, behaviour change and self-management.
- Dr Amit Kaura is the Chief Medical Officer for DDM Health but his involvement in the project is as an academic at Imperial College London. DDM Health will not be

For peer review only - http://bmjopen.bmj.com/site/about/guidelines.xhtml

paying Dr Kaura for his involvement, nor will they receive any project funds for his involvement.

- Dr Jonathan M. Hazlehurst declares advisory work previously for Novo Nordisk as well as honoraria for speaking engagements from Novo Nordisk and Eli Lilly and support for attendance at academic meetings from Novo Nordisk as well as Eli Lilly as well as academic funding from the NIHR.
- No other members of the research team have declared any conflicts of interest.

Patient and Public involvement [PPI]

PPI were involved in the design of the research, please refer to the methods section for further information

Funding Statement

This study/project is funded by the NIHR [grant number NIHR208100 https://fundingawards.nihr.ac.uk/award/NIHR208100]. The views expressed are those of the author[s] and not necessarily those of the NIHR or the Department of Health and Social Care.

Role and responsibilities of the Sponsor

The University of Warwick will act as study sponsor. The study will be conducted in accordance with Sponsor's Standard Operating procedures. The University of Warwick has a specialist insurance policy in place which would operate in the event of any participant suffering harm as a result of their involvement in the study Zurich Municipal Insurance. NHS indemnity operates in respect of the clinical treatment that is provided. Further information can be found in supplementary table 3.

For peer review only - http://bmjopen.bmj.com/site/about/guidelines.xhtml

Role and responsibilities of the Funder

This study is funded by the National Institute for Health and Care Research Invention for Innovation [NIHR i4i] programme [NIHR208100]. Supplementary figure 2 displays the design and management of the trial are entirely independent of the funder.

Data Availability Statement

Included in supplementary material table 1.

For peer review only

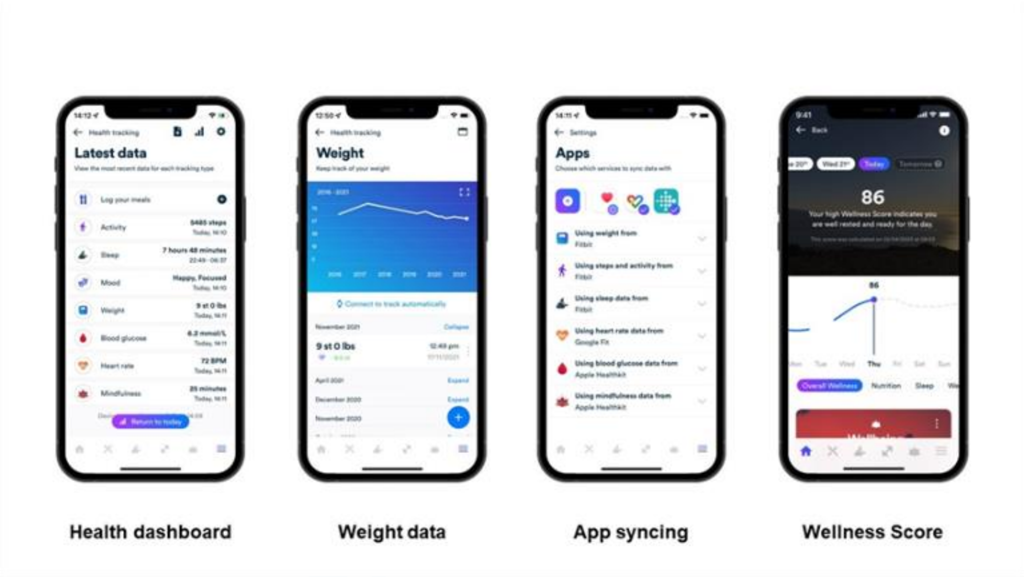

For peer review only

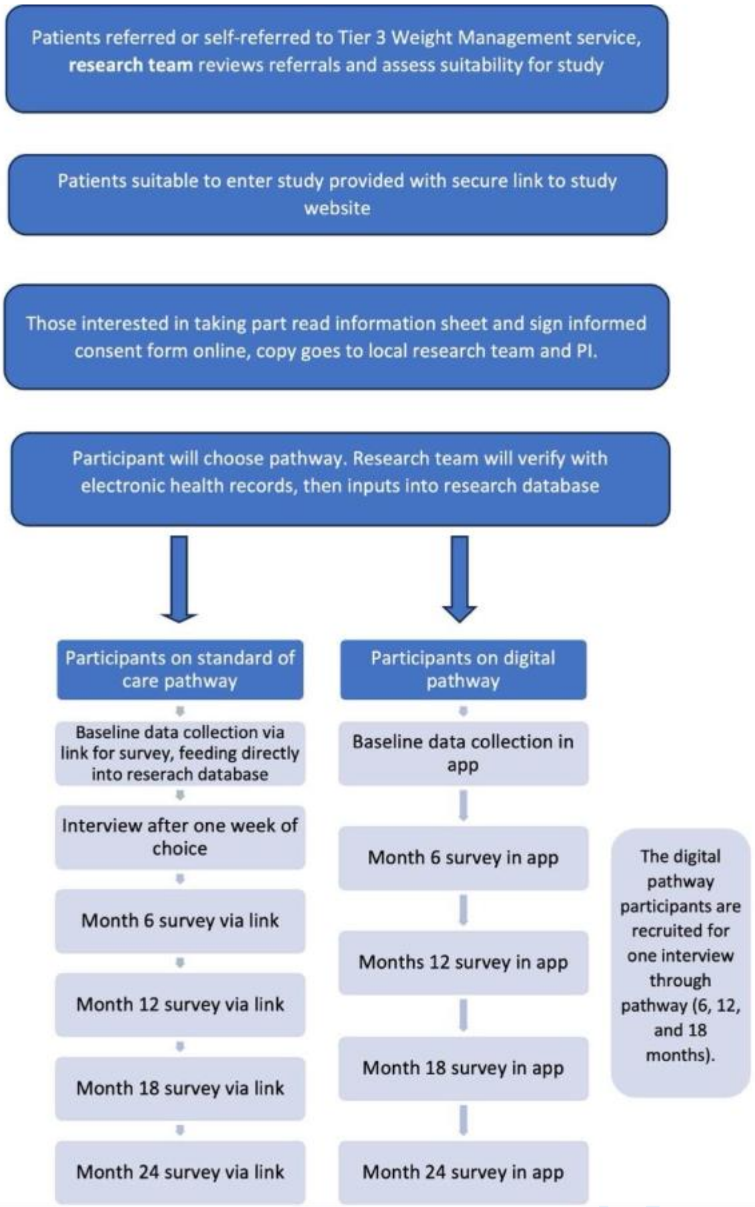
Patients referred or self-referred to Tier 3 Weight Management service, research team reviews referrals and assess suitability for study
Patients suitable to enter study provided with secure link to study website
Those interested in taking part read information sheet and sign informed consent form online, copy goes to local research team and PI.
Participant will choose pathway. Research team will verify with electronic health records, then inputs into research database
Participants on standard of care pathway
Baseline data collection via link for survey, feeding directly into reserach database
Interview after one week of choice
Month 6 survey via link
Month 12 survey via link
Month 18 survey via link
Month 24 survey via link
Participants on digital pathway
Baseline data collection in app
Month 6 survey in app
Months 12 survey in app
Month 18 survey in app
Month 24 survey in app
The digital pathway participants are recruited for one interview through pathway (6, 12, and 18 months).

Data flows between organisations within the W8Buddy Study

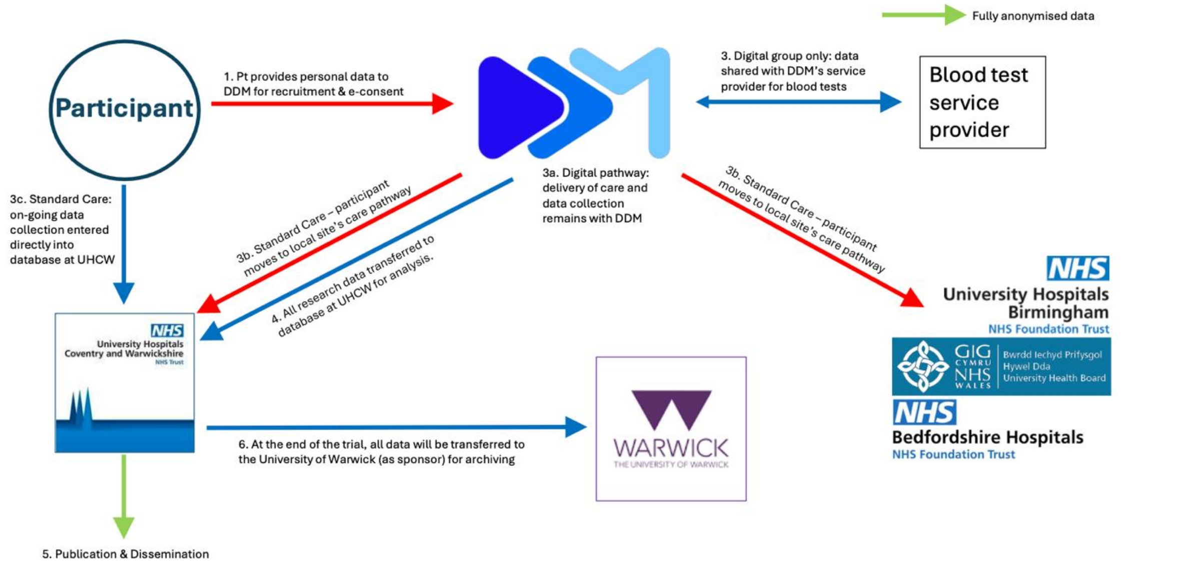

Order of data flows in W8Buddy Study

1. Participant provides personal information to DDM to facilitate online recruitment and e-consent.
2. After giving informed consent, participant chooses pathway:
   a) Digital – participant continues to provide data to DDM for delivery of care and data collection
   b) Standard Care – participant moves to local site's care pathway
   c) Standard Care – on-going data collection for study is entered directly into database at UHCW
3. Digital group only: data shared with DDM's service provider for blood tests.
4. All research data transferred to database at UHCW for analysis.
5. Following completion of the study, the findings will be published and disseminated using fully anonymised data.
6. At the end of the trial, all data will be transferred to the University of Warwick (as sponsor) for archiving

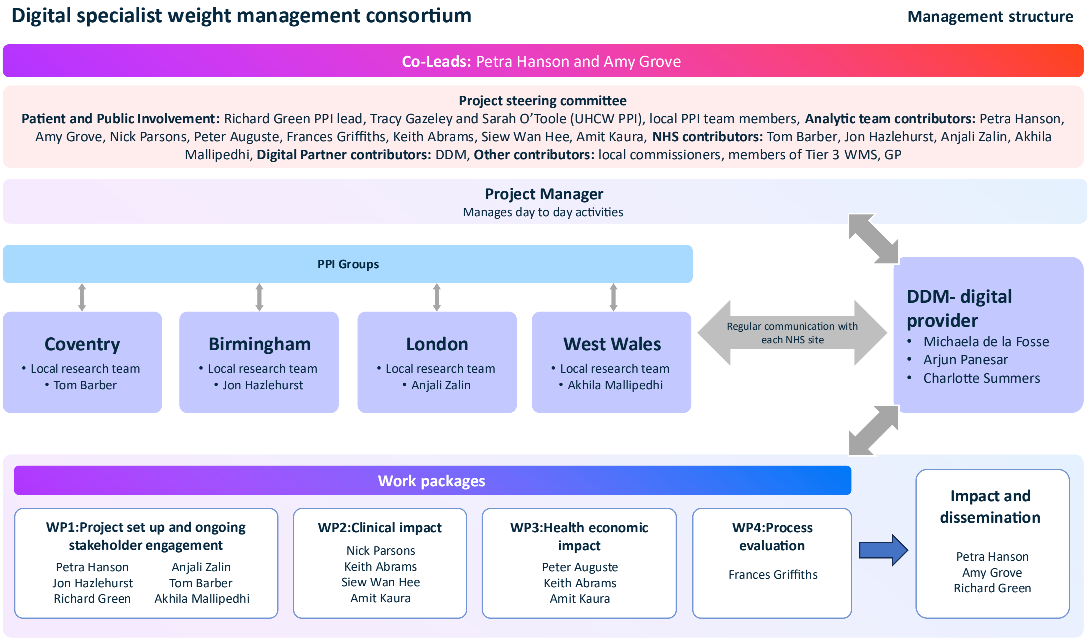
Digital specialist weight management consortium
Management structure
Co-Leads: Petra Hanson and Amy Grove
Project steering committee
Patient and Public Involvement: Richard Green PPI lead, Tracy Gazeley and Sarah O'Toole (UHCW PPI), local PPI team members, Analytic team contributors: Petra Hanson, Amy Grove, Nick Parsons, Peter Auguste, Frances Griffiths, Keith Abrams, Siew Wan Hee, Amit Kaura, NHS contributors: Tom Barber, Jon Hazlehurst, Anjali Zalin, Akhila Mallipedhi, Digital Partner contributors: DDM, Other contributors: local commissioners, members of Tier 3 WMS, GP
Project Manager
Manages day to day activities
PPI Groups
Coventry
• Local research team
• Tom Barber
Birmingham
• Local research team
• Jon Hazlehurst
London
• Local research team
• Anjali Zalin
West Wales
• Local research team
• Akhila Mallipedhi
Regular communication with each NHS site
DDM- digital provider
• Michaela de la Fosse
• Arjun Panesar
• Charlotte Summers
Work packages
WP1:Project set up and ongoing stakeholder engagement
Petra Hanson
Jon Hazlehurst
Richard Green
Anjali Zalin
Tom Barber
Akhila Mallipedhi
WP2:Clinical impact
Nick Parsons
Keith Abrams
Siew Wan Hee
Amit Kaura
WP3:Health economic impact
Peter Auguste
Keith Abrams
Amit Kaura
WP4:Process evaluation
Frances Griffiths
Impact and dissemination
Petra Hanson
Amy Grove
Richard Green

Supplementary table 1. Data management

Information	Description
Data collection tools and source document identification	Study data will be collected on the screening logs, W8Buddy App and validated questionnaires (see Table 1) electronically. Health record data for each site will be accessed by local research team. An online validated, GCP compliant, Electronic Data Capture system will be used to record and store study data, this will be the main Study Database. Individual user log-in access to this database will be granted to only those in the study team that require it for the performance of their role. The W8Buddy App will collect data for participants in the digital arm of the study, then transfer this data in a secure way to the main study database. Only research team and members of the DDM team will have access to the main study database.<br>Source data for standard care procedures will be from hospital medical records. Other outcome measures not collected routinely will either be collected directly from the patient, their clinical care team, or App collaborators and entered directly on to the App/Database.<br>Participants will be able to complete the outcome questionnaires by phone, email or online if they prefer. If done online, they will be emailed a secure, unique link to complete the questionnaires, where their answers will directly input onto the database.<br>The research team at UHCW (see page 1 for details) will maintain oversight of the Study Database during the study to ensure that any missing data is supplied in a timely manner.<br><br>Semi-structured interviews will be audio recorded using teleconferencing software (e.g., Zoom) and transcribed by an external transcription company. The data will be stored on secure University of Warwick computers and accessed only by research team located at Warwick.<br><br>Data entered into the app will be stored in secure Azure cloud.<br>DDM's Data Protection and Information Governance Policy details how we comply to and exceed UK GDPR requirements through:<br>• Consent is requested on referral (website or telephone), paper-based for face-to-face and digitally before using the app. Our privacy policy is available on our website, physically at face-to-face sessions and on in-app/platform. On signup (and signposted in-app), users are informed on how their data will be used, who will access it, their rights and consent/opt-in to

For peer review only

	sharing anonymised data (including with Commissioner and OHID). The app locks users out after several unsuccessful access attempts and logs users out after periods of inactivity. All user access is logged (IP, time, date, browser details) in case of safeguarding or data issues.<br>• Individuals have the right to access their personal data, rectify incorrect data, erase their data and restrict or object to its processing. This is specified in our privacy policy.<br>• We are aware that if we have data breaches, we report within 72 hours of becoming aware of the breach to the Information Commissioners' Office and University's Legal & Compliance Services, in accordance with the University's data protection policies and the ICO as per DDM policies. DDM has a Full Business Continuity Plan which includes a Disaster Recovery Plan tested quarterly.<br>• Service-users' personal data is never transferred outside of the UK. DDM's data servers are hosted by Microsoft Azure in the UK and are fully encrypted-at-rest and in-transit using AES256 cryptography.<br>• Staff are provided an individual account with restricted privileges, dependent on their tasks/role. Any data which is invalid, irrelevant or no longer required (including user removal requests) is securely deleted.<br>• All staff undergo mandatory data/information governance training bi-annually and perform a self-assessment (e.g. passwords, UK GDPR) to identify training needs to provide further formal training.<br>• All information collected, stored and communicated (internally/externally) is carried out in-line with the Data Protection Policy through use of secure digital patient records, encrypted email and private communication software.<br>• All data transfer is done via FTPS (Secure FTP) or encrypted data transferral method.<br>• Paper data and local servers are stored in a locked, CCTV-protected room in DDM Headquarters. All data on the servers is password protected and encrypted using 256-AES (BitLocker on Windows).<br>• One individual via USB/over FTPS takes backups daily. Backups are stored in-the-cloud on a UK-based 256-AES encrypted hard-drive. Paper data is stored in a locked room. Only the DPO has the login details and keys to the data room. All passwords used for data handling

For peer review only

	are 28 characters long and changed weekly. Access is only granted to authorised staff for limited purposes and is time restricted.
Data handling and record keeping	Screening and recruitment logs of all patients approached to take part, and participants enrolled in the study will be held at each site. Upon confirmation of eligibility, participants will be assigned a unique study ID via the study website, which will be used to identify all documents associated with that participant for the duration of the study.<br>It is the responsibility of the PI at each site to ensure that the data that has been submitted is verified and accurate in reflecting the clinical outcome of the participants.<br>This is electronic-based study, with no paper-based collection. Data will be stored in on secure cloud-based databases including the W8Buddy App, with restricted access and regular back-ups in the cloud-based server.<br>Personal data collected during the study will be handled and stored in accordance with the UK GDPR. Handling of personal data will be clearly documented in the participant information sheet and consent obtained.<br>Following all query resolution, the database will be exported for statistical analysis. These procedures along with data entry instructions will be detailed in a Data Management Plan (DMP) produced by the study coordinator and statistician.
Access to data	Direct access will be granted to authorised representatives from the Sponsor, host institution and the regulatory authorities to permit study-related monitoring, audits and inspections - in line with participant consent.<br>Delegated research team members will be responsible for entering data into the database. This may include the site Principal Investigator (PI), research nurses, and study team members based at UHCW, UHB, LTT, and HDUHBT. Access to the data will be restricted to the central study team only. Additionally, all staff granted access to the database will be listed in the delegation log and must hold Good Clinical Practice (GCP) certification.
Archiving	Following the resolution of queries and confirmation of study close-out by the Chief Investigator, all essential documentation will be transferred to a third-party archiving service, which provides suitable fire- and water-resistant facilities. Study files will be archived for a period of 10 years.

For peer review only

	Access to the study documentation will be restricted to named individuals within the study team with express permission from the Chief Investigator.

Supplementary table 2. Safety reporting

Information	Description
Adverse event	An AE is any untoward medical occurrence experienced by a patient while participating in the trial. This includes occurrences which are not necessarily caused by or related to the trial intervention.
Serious adverse event	A SAE is characterised as an untoward occurrence deemed to be related to the intervention under investigation that:<br>1. Results in death within 30 days of the intervention<br>2. Is life-threatening<br>3. Requires hospitalisation or an extension of an existing inpatients' hospitalisation<br>4. Results in persistent or significant disability or incapacity<br>5. Consists of a congenital anomaly or birth defect<br>6. Otherwise considered medically significant by the investigator
Reporting	• The Chief Investigator (or designated representative) will assess all reported SAEs for causality and expectedness.<br>• All intervention related AEs must be documented using the relevant electronic Case Report Form (eCRF), patients will report these to the research team who will then enter this data in the eCRF.<br>• SAEs should be reported for any occurrence from the time informed consent is obtained until study completion.<br>All participants will have contact details to the research team and will be able to inform the research team of any AEs/SAEs occurring between study visits. Participants will be asked to

For peer review only

	contact the research nurse as soon as possible with any potential adverse events. The research nurse will call the patient to discuss the adverse event; this will be escalated as per each standard hospital protocols and will be escalated to each site PI for investigation and recording as required<br>All SAEs must be reported to the study team within 24 hours of the Principal Investigator (or designated representative) becoming aware of the event. This should be done by completing the SAE eCRF and emailing:<br>Email address: W8Buddystudyofficeinbox@uhcw.nhs.uk
Follow up	SAEs must be followed until clinical recovery is complete or the condition stabilises. Outcomes of SAEs should be reported to the study team promptly using the relevant eCRF/CRF. These should be sent to the W8Buddy Study inbox (W8Buddystudyofficeinbox@uhcw.nhs.uk) as soon as the Principal Investigator (or designated representative) is aware of the outcome.<br><br>**EVENTS THAT DO NOT REQUIRE REPORTING AS AN ADVERSE EVENT OR SERIOUS ADVERSE EVENT**<br>This trial recruits' participants with pre-existing long-term conditions (e.g., type 2 diabetes, hypertension), likely requiring medical attention during the trial. Given:<br>• The CE marking of the W8Buddy intervention<br>• Safety not being an outcome of interest<br>A risk-based approach to reporting will apply, in which only AEs deemed related to the W8Buddy intervention will be reported.<br>In addition, the following events will not require reporting as SAEs:<br>• Scheduled hospital visits (e.g., routine outpatient appointments, elective surgeries).<br>• Visits related to pre-existing medical conditions or their progression.<br>• Foreseeable or predictable SAEs attributed to terminal illness or similar conditions.<br>• SAEs related to standard care medications

For peer review only

Supplementary table 3. Study oversight

Information	Description
Roles and responsibilities of the sponsor	The University of Warwick will act as study sponsor. The study will be conducted in accordance with Sponsor's Standard Operating procedures. The University of Warwick has a specialist insurance policy in place which would operate in the event of any participant suffering harm as a result of their involvement in the study Zurich Municipal Insurance. NHS indemnity operates in respect of the clinical treatment that is provided.
Roles and responsibilities of the funder	This study is funded by the National Institute for Health and Care Research Invention for Innovation (NIHR i4i) programme (NIHR208100).
Study manage/co-ordinator	The Trial Co-ordinator / Manager will have responsibility for overseeing day to day coordination of the trial and reporting regularly to the TSC. The Study Manager's responsibilities include, but are not limited to:<br>• Coordinating protocol development, patient and trial management documents<br>• Correspondence with study funder and tracking of progress against agreed milestones<br>• Setting up and maintaining the Trial Master File<br>• Ensuring necessary approvals are in place before the start of the trial at each site<br>• Providing training to trial personnel<br>• Providing data management support; including data input, maintenance of the trial database and raising of queries<br>• Producing trial progress reports and coordinating TSC meetings and minutes<br>• Ensuring data security and quality and ensuring data protection laws are adhered to<br>• Ensuring complete records are in place for audit and monitoring purposes<br>• Ensuring the trial is conducted in accordance with the ICH GCP<br>• Archiving all original trial documents including the data forms in line with UHCW NHS Trust policy.

For peer review only

Site principal investigators	Site Principal Investigator responsibilities include, but are not limited to:<br>• Ensuring that the trial is conducted as set out in the protocol and supporting documents<br>• Delegating trial related responsibilities only to suitably trained and qualified personnel and ensuring that those with delegated responsibilities fully understand and agree to the duties being delegated to them<br>• Ensuring that CVs and evidence of appropriate training for all Site staff are available in the Trial Site File<br>• Ensuring that all delegated duties are captured in the study Delegation Log<br>• Ensuring all Adverse Events are documented and reported promptly to the Trial Manager<br>• Accountability for trial treatments at their site<br>• Ensuring the trial is conducted in accordance with ICH GCP principles.<br>• Allowing access to source data for monitoring, audit and inspection<br>• Ensuring that all source data is complete and provided to the Trial Manager at regular intervals.
Monitoring, audit and inspection	The study will be monitored by the study management group to ensure that the study is being conducted as per protocol, adhering to Research Governance and GCP. Central monitoring activities will be performed such as data quality checks. The approach to, and extent of, monitoring will be specified in a study monitoring plan determined by the risk assessment undertaken prior to the start of the study.<br><br>The recruiting site is obliged to assist the sponsor in monitoring the study. These may include hosting site visits, providing information for remote monitoring, or putting procedures in place to monitor the study internally.<br><br>The study may be monitored by the Warwick University representative of the Sponsor, to ensure that the study is being conducted as per protocol, adhering to Research Governance and GCP. The approach to, and extent of, monitoring will be specified in a study monitoring plan determined by the risk assessment undertaken prior to the start of the study.

For peer review only

Ymchwil Iechyd a Gofal Cymru
Health and Care Research Wales

NHS
Health Research Authority

Dr Petra Hanson
Clinical Lecturer
University of Warwick
Warwick Medical School, University of Warwick,
Coventry
CV4 7AL

Email: approvals@hra.nhs.uk
HCRW.approvals@wales.nhs.uk

29 July 2025

Dear Dr Hanson

HRA and Health and Care Research Wales (HCRW) Approval Letter

**Study title:**	**Long-term health and economic impact of using digital tool Gro Health W8Buddy to support people accessing specialist weight management services**
**IRAS project ID:**	**355370**
**Protocol number:**	**1.0**
**REC reference:**	**25/EM/0147**
**Sponsor**	**Warwick University**

I am pleased to confirm that **HRA and Health and Care Research Wales (HCRW) Approval** has been given for the above referenced study, on the basis described in the application form, protocol, supporting documentation and any clarifications received. You should not expect to receive anything further relating to this application.

Please now work with participating NHS organisations to confirm capacity and capability, in line with the instructions provided in the "Information to support study set up" section towards the end of this letter.

How should I work with participating NHS/HSC organisations in Northern Ireland and Scotland?

HRA and HCRW Approval does not apply to NHS/HSC organisations within Northern Ireland and Scotland.

If you indicated in your IRAS form that you do have participating organisations in either of these devolved administrations, the final document set and the study wide governance report (including this letter) have been sent to the coordinating centre of each participating nation. The relevant national coordinating function/s will contact you as appropriate.

For peer review only

Please see IRAS Help for information on working with NHS/HSC organisations in Northern Ireland and Scotland.

How should I work with participating non-NHS organisations?
HRA and HCRW Approval does not apply to non-NHS organisations. You should work with your non-NHS organisations to obtain local agreement in accordance with their procedures.

What are my notification responsibilities during the study?

The standard conditions document "*After Ethical Review – guidance for sponsors and investigators*", issued with your REC favourable opinion, gives detailed guidance on reporting expectations for studies, including:

- Registration of research
- Notifying amendments
- Notifying the end of the study

The HRA website also provides guidance on these topics, and is updated in the light of changes in reporting expectations or procedures.

Who should I contact for further information?
Please do not hesitate to contact me for assistance with this application. My contact details are below.

Your IRAS project ID is **355370**. Please quote this on all correspondence.

Yours sincerely,
Chris Kitchen

Email: approvals@hra.nhs.uk

Copy to: *Mrs Carole Harris*

For peer review only

List of Documents

The final document set assessed and approved by HRA and HCRW Approval is listed below.

Document	*Version*	*Date*
Contract/Study Agreement template [W8Buddy IRAS 355370 mNCA_July_2022]	v2.3	
Evidence of Sponsor insurance or indemnity (non NHS Sponsors only) [UoW Insurance]	N/A	02 July 2024
GP/consultant information sheets or letters [W8Buddy_GPLetter_v1.0_ 06Jun2025]	v1.0	08 May 2025
Interview schedules or topic guides for participants [W8Buddy_ Topic guides for interviews_v1.0_06Jun2025]	v1.0	06 June 2025
Interview schedules or topic guides for participants [W8Buddy_ Topic guides for HCP interviews_v1.0_12 Jun2025]	1.0	12 June 2025
IRAS Application Form [IRAS_Form_10062025]	N/A	10 June 2025
Letter from funder [Outcome Letter NIHR208100]	N/A	03 September 2024
Letter from sponsor [SOC.11 24-25 W8Buddy Sponsorship Approval Letter]	N/A	06 June 2025
Non-validated questionnaire [W8Buddy_CRSI_v1.0_06Jun2025]	1.0	06 June 2025
Organisation Information Document [Organisation_Information_Document_NonCommercial_v1-8_April_2024+W8Buddy(2)]		01 June 2025
Other [W8Buddy_Websiteconsentprocess_v1.0_06Jun2025]	v1.0	06 June 2025
Other [W8Buddy_Storyboard_v1.0_13Jun2025]	vX.0	13 June 2025
Other [W8Buddy Website Screenshots_v1.0_13Jun2025]	v1.0	13 June 2025
Other [Ethics Response 11Jul2025]	N/A	11 July 2025
Participant consent form [W8Buddy_ConsentForm_HCP_v2.0_ 11Jul2025]	2.0	11 July 2025
Participant consent form [W8Buddy_ConsentForm_v1.0_06Jun2025]	v1.0	06 June 2025
Participant information sheet (PIS) [W8Buddy_CondensedPIS_v1.0_11Jul2025]	v1.0	11 July 2025
Participant information sheet (PIS) [W8Buddy_PIS_HCP_v2.0_11Jul2025]	v2.0	11 July 2025
Participant information sheet (PIS) [W8Buddy_PIS_v2.0_11Jul2025]	v2.0	11 July 2025
Research protocol or project proposal [W8Buddy Protocol_ v1.0_06Jun2025]	v1.0	06 June 2025
Schedule of Events or SoECAT [Funder Export_342299_SoECAT_Gro Health W8Buddy in Specialist Weight Management Services]		
Summary CV for Chief Investigator (CI) [PH CV june 2025]		06 June 2025
Validated questionnaire [PHQ-8_English-UK]		
Validated questionnaire [GAD7_English for the UK]		
Validated questionnaire [Effective_UK (English) EQ-5D-5L Paper Self-Complete v1.2]	v1.2	
Validated questionnaire [BEDS-7_AU1.0_eng-USori]		
Validated questionnaire [Satisfaction With Life Scale]		
Validated questionnaire [Karolinska Sleepiness Scale]		

For peer review only

IRAS project ID	355370

Information to support study set up

The below provides all parties with information to support the arranging and confirming of capacity and capability with participating NHS organisations in England and Wales. This is intended to be an accurate reflection of the study at the time of issue of this letter.

Types of participating NHS organisation	Expectations related to confirmation of capacity and capability	Agreement to be used	Funding arrangements	Oversight expectations	HR Good Practice Resource Pack expectations
Research activities and procedures as per the protocol and other study documents will take place at participating NHS organisations.	Research activities should not commence at participating NHS organisations in England or Wales prior to their formal confirmation of capacity and capability to deliver the study in accordance with the contracting expectations detailed.	An Organisation Information Document has been submitted but the sponsor is intending to use a separate agreement. The sponsor has supplied the unmodified model agreement and intends to use this with participating NHS organisations.	Study funding arrangements are detailed in the Organisation Information Document.	A Principal Investigator should be appointed at participating NHS organisations.	Where an external individual who does not already hold an NHS employment contract will be conducting any of the research activities that will be undertaken at this site type then they would be expected to hold an Honorary Research Contract. This should be issued be on the basis of a Research Passport (if university employed) or an NHS to NHS confirmation of pre-engagement checks letter (if NHS employed). These should confirm Occupational Health Clearance. These should confirm enhanced DBS checks and appropriate barred list checks.<br>External staff holding pre-existing NHS employment contracts, or staff only

					undertaking survey or interview activities should obtain a Letter of Access. This should be issued be on the basis of a Research Passport (if university employed) or an NHS to NHS confirmation of pre-engagement checks letter (if NHS employed). These should confirm Occupational Health Clearance if there is direct contact with participants These should confirm standard DBS checks.

Other information to aid study set-up and delivery

This details any other information that may be helpful to sponsors and participating NHS organisations in England and Wales in study set-up.
The applicant has indicated that they intend to apply for inclusion on the NIHR CRN Portfolio.

For peer review only

Supplementary Table 4

Data collected	When and how data collected from digital (D)/ Standard care (SC) group	Data used for assessment of:
**Weight, height, and BMI**	Month 0,6,12,18,24.<br><br>D: app, dashboard<br><br>SC: survey, clinical notes	Weight loss
**Metabolic markers (HBA1c, lipids)**	Month 0,6,18.<br><br>D: app, dashboard (the same standard of care blood tests will be done remotely instead using clinical phlebotomy partners and samples will be returned for analysis, then discarded as per usual clinical procedures.<br><br>SC: survey, clinical notes (only routine blood test results will be collected in the standard of care pathway, as per usual standard of care for NHS hospitals)	Metabolic impact
**Blood pressure**	Month 0,6,18.<br><br>D: app, dashboard<br><br>SC: survey, clinical notes	Cardiovascular marker
**Waist circumference**	Month 0,6,12,18,24.	Body composition changes

For peer review only

	D: app, dashboard<br><br>SC: survey, clinical notes	
**Health related quality of life (EQ-5D-5L)**	Month 0,6,12,18,24.<br><br>D: app<br><br>SC: survey	Cost-effectiveness and quality of life
**PHQ8, satisfaction with life scale, Karolinska sleepiness scale, GAD7, BEDS-7**	Month 0,6,12,18,24.<br><br>D: app<br><br>SC: survey	Biopsychosocial outcomes
**Clinical endpoints (stroke, myocardial infarction)**	Month 12, 24, 36, 48, 60<br><br>D: app, dashboard<br><br>SC: clinical notes	Major adverse cardiovascular events
**Distance to local NHS facility for participants**	Month 0.<br><br>Research team review of GP notes for D and SC	Health economic calculations
**Time needed to travel to local NHS facility for participants**	Month 0.<br><br>Research team review of GP notes for D and SC, plus	Health economic calculations

For peer review only

	D: app<br><br>SC: survey	
**Date when participants dropped out of study if applicable**	As applicable.<br><br>D: app<br><br>SC: survey	Accurate tracking
**Reason why participant on standard care dropped out of study if applicable**	SC: Contact with research nurse	Why participants drop out of standard care
**Number of consented participants**<br>• **choosing the digital pathway**<br>• **starting on the pathway**<br>• **dropping out**	Month 0-24.<br><br>Reviews of dashboard and research database	Willingness and engagement level of digital pathway<br><br>Sampling frame for participant interviews
**Participant baseline characteristics: comorbidities, sex, age, ethnicity, disabilities, geographical region, socioeconomic status**	Month 0.<br><br>Research team review of GP notes for D and SC, plus<br><br>D: app<br><br>SC: survey	Stratification of interview sample, sensitivity analyses, predictors of cost-effectiveness and engagement with digital pathway
**Comorbidities at follow up (hypertension, obstructive sleep apnoea, osteoarthritis, non-**	Month 6, 12, 18, 24	Assessment of diseases associated with obesity

For peer review only

**alcoholic fatty liver disease, polycystic ovarian syndrome, type 2 diabetes, dyslipidaemia)**	D: app, review of health records<br><br>SC: survey, review of health records	
**Type of concomitant and weight-management medication, dose, and prescription dates, whether NHS or non-NHS prescribed**	Month 0,6,12,18,24<br><br>D: app<br><br>SC: survey, clinical notes	Calculation of cost<br><br>Medication adherence
**Reason for stopping the medicine, including side effects (including physical and psychological effects as per STEP 5 trial. These include nausea, diarrhoea, constipation, vomiting, abdominal pain, dyspepsia, gastroenteritis, decreased appetite, headache, eructation, nasopharyngitis, headache, back pain, upper respiratory tract infection, gallbladder related disorders, cholelithiasis, pancreatitis, cardiovascular disorders, injection site reactions, hypoglycaemia, psychiatric disorders , reaching predefined weight loss goals, lack of adherence to prescribed medicine**	Month 0,6,12,18,24<br><br>D: app, dashboard<br><br>SC: survey, clinical notes	Cost-effectiveness, Biomedical explanation of adherence rates

For peer review only

**Use of and response to digital pathway**	Month 0-24<br><br>D: app<br><br>SC: appointment records and clinical notes<br><br>Information collected on all patients who switch from SC to D and vice versa, even though this is expected to be low	Cost-effectiveness,<br><br>Adherence to digital pathway; variation in adherence by patient characteristics<br><br>User experience and process evaluation
**Adverse events, including only intervention-related adverse effects**	Month 0-24<br><br>D: consultation with clinicians on dashboard, medication tracking, captured at real-time<br><br>SC: clinical notes, surveys at month 6,12,18,24	Cost-effectiveness, understanding the experience of users, creating blueprint for safe incorporation of digital pathway

For peer review only

**Healthcare resource use: Data from providers about the multidisciplinary service, resource use (e.g. rates of referral to bariatric surgery)**<br><br>**Data from participants on number of GP visits, unplanned admissions to A&E**	Month 0,6,12,18,24<br><br>D: app and reports from digital provider<br><br>SC: survey, clinical notes	Cost-effectiveness
**Cost associated with implementing and maintaining digital pathway**	D: Digital provider	Cost-effectiveness
**Cost estimates for specialist WMS**	Month 0,6,12,18,24<br><br>SC: clinical records, survey	Cost-effectiveness
**Implementation, adaptations, and contextual influences**	Observation of pathway delivery team meetings, meeting notes, semi-structured interviews with implementers and clinical staff delivering intervention.<br><br>During initial implementation (month 0) and in months 12 & 18	Implementation process to inform formative feedback<br><br>Manual for future NHS implementation and process evaluation
**Availability of digital pathway and medicine to participant including**<br><br>**staff training and quality assurance**	D: app and related organisational records	Fidelity of delivery of intervention<br><br>Experience of delivery of intervention

For peer review only

	SC: clinical records and related organisational records throughout delivery	Process evaluation
**Brief structured interview with study participants not choosing digital pathway**	Within approx. 1 week of choice<br><br>Remote interview	Why participants do not choose digital pathway (process evaluation)
**Brief structured interview with study participants not adherent to digital pathway and/or medicine**	Sample for participants demonstrating early/late non-adherence or on/off adherence<br><br>Remote interview	Why participants drop out/return to digital pathway/medicine process evaluation)
**Semi-structured interviews with study participants using digital pathway**	Sample stratified by gender and progress through pathway (6, 12, 18 months)<br><br>Remote interview	Understand how user engages with digital pathway, why, perceived impacts and user understanding of mechanism of impact (process evaluation).

Abbreviations: BMI, Body mass index; **EQ-5D-5L**, EuroQol five dimensions and levels; GAD, general anxiety disorder scale; PHQ-8, Patient Health Questionnaire 8, BEDS-7, Binge eating disorder screener

For peer review only

BMJ Open

A protocol for a cohort observational study using real-world evidence to evaluate the long-term health and economic impact of a digital tool Grohealth W8Buddy supporting access to specialist weight management services

Journal:	*BMJ Open*
Manuscript ID	bmjopen-2025-109111.R2
Article Type:	Protocol
Date Submitted by the Author:	11-Dec-2025

Complete List of Authors:	Deo, Pranay; Warwick Medical School<br>Grove, Amy; University of Birmingham<br>Zhang, Mengxi; Warwick Medical School,<br>Abrams, Keith; University of Warwick, Department of Statistics; University of York, Centre for Health Economics<br>Auguste, Peter; University of Birmingham, School of Social Policy and Society<br>Barber, Thomas; University of Warwick; University Hospitals Coventry and Warwickshire NHS Trust<br>Gazeley, Tracy; University Hospitals Coventry and Warwickshire NHS Trust<br>Green, Richard; University Hospitals Coventry and Warwickshire NHS Trust<br>Griffiths, Frances; University of Warwick, Warwick Medical School<br>Hazlehurst, Jonathan; Birmingham Health Partners; University Hospitals Birmingham NHS Foundation Trust, Department of Diabetes and Endocrinology; University of Birmingham<br>Hee, Siew Wan; University Hospitals Coventry and Warwickshire NHS Trust, Research and Development; University of Warwick Medical School<br>Kaura, Amit; Imperial College London, Faculty of medicine<br>Mallipedhi, Akhila; Hywel Dda Health Board<br>O'Toole, Sarah; University Hospitals Coventry and Warwickshire NHS Trust<br>Panesar, Arjun; DDM Health,<br>Parsons, Nicholas; Warwick Medical School, Warwick Medical School<br>Scott, Emma; University of Warwick - Coventry Campus, Warwick medical school<br>Summers, Charlotte; DDM Technology House University of Warwick Coventry, UK CV4 7EZ<br>Thind, Manreet; University Hospitals Coventry and Warwickshire NHS Trust<br>Thorpe, Marie; University of Warwick, Warwick medical school<br>Zalin, Anjali ; Barts Health NHS Trust<br>Hanson, Petra; University of Warwick, Applied Health; University Hospitals Coventry and Warwickshire NHS Trust; South Warwickshire NHS Foundation Trust

**Primary Subject Heading**:	Health services research
Secondary Subject Heading:	Evidence based practice
Keywords:	Obesity, Digital Technology, Clinical Protocols, Mobile Applications

SCHOLARONE™ Manuscripts

A protocol for a cohort observational study using real-world evidence to evaluate the long-term health and economic impact of a digital tool Grohealth W8Buddy supporting access to specialist weight management services

1. **Pranay Singh Deo** – University of Warwick Medical School Coventry CV4 4AL pranay.deo@warwick.ac.uk
2. **Amy Grove** - A.L.Grove@bham.ac.uk - University of Birmingham, Centre for Evidence and Implementation Science, Edgbaston, Birmingham B15 2TT
3. **Mengxi Zhang** - mengxi.zhang@warwick.ac.uk, University of Warwick Medical School Coventry CV4 4AL
4. **Keith Abrams** - keith.abrams@warwick.ac.uk, University of Warwick Medical School Coventry CV4 4AL
5. **Peter Auguste** - p.e.auguste.1@bham.ac.uk, University of Birmingham Health Services Management Centre, Birmingham B15 2TT
6. **Thomas M Barber** - t.barber@warwick.ac.uk, Division of Biomedical Sciences, Warwick Medical School, University of Warwick, Coventry, CV4 7AL, UK Warwickshire Institute for the Study of Diabetes, Endocrinology and Metabolism, University Hospitals Coventry and Warwickshire, Clifford Bridge Road, Coventry, CV2 2DX, UK Research Centre for Health and Life Sciences, Coventry University, Richard Crossman Building, Jordan Well, Coventry, CV1 5RW, UK
7. **Tracy Gazeley** - tracy.gazeley@uhcw.nhs.uk, University Hospitals Coventry & Warwickshire NHS trust, Clifford Bridge Road, Coventry CV2 2DX PPI
8. **Richard Green** - rmguk@hotmail.com, University Hospitals Coventry & Warwickshire NHS trust, Clifford Bridge Road, Coventry CV2 2DX PPI
9. **Frances Griffiths** - f.e.griffiths@warwick.ac.uk University Of Warwick Medical School Coventry CV4 4AL
10. **Jonathan Hazlehurst** - jonathan.hazlehurst3@uhb.nhs.uk, University Hospital Birmingham Mindelsohn Way, Birmingham B15 2GW
11. **Siew Wan Hee** - s.w.hee@warwick.ac.uk, Warwick Medical School, University of Warwick, Coventry CV4 7AL, and Research and Development, University Hospitals Coventry and Warwickshire NHS Trust, Clifford Bridge Road, Coventry CV2 2DX
12. **Amit Kaura** - a.kaura@imperial.ac.uk, Imperial College London Exhibition Road, London SW7 2AZ , Faculty of Medicine
13. **Akhila Mallipedhi** - akhila.mallipedhi@wales.nhs.uk, Hyweldda Health Board, 1 Penlan Road, Carmarthen SA31 1DS
14. **Sarah O'Toole** - sarah.otoole@uhcw.nhs.uk, University Hospitals Coventry & Warwickshire NHS trust, Clifford Bridge Road, Coventry CV2 2DX
15. **Arjun Panesar** - arjun@ddm.health, Diabetes Digital Management [DDM] Technology House Sir William Lyons Road, Coventry CV4 7EZ

For peer review only

For peer review only - http://bmjopen.bmj.com/site/about/guidelines.xhtml

16. **Nicholas Parsons** - nick.parsons@warwick.ac.uk, University Of Warwick Medical School Coventry CV4 4AL
17. **Emma Scott -** e.j.scott@warwick.ac.uk, University Of Warwick Medical School Coventry CV4 4AL
18. **Charlotte Summers** - charlotte@ddm.health, Diabetes Digital Mangement [DDM] Technology House Sir William Lyons Road, Coventry CV4 7EZ
19. **Manreet Thind** - manreet.thind@uhcw.nhs.uk, University Hospitals Coventry & Warwickshire NHS trust, Clifford Bridge Road, Coventry CV2 2DX
20. **Marie Thorpe** - marie.thorpe@warwick.ac.uk, University Of Warwick Medical School Coventry CV4 4AL
21. **Anjali Zalin** - anjali.zalin3@nhs.net, Barts Health NHS Trust, 9 Prescot Street, London E1 8PR
22. **Petra Hanson - Petra.hanson.1@warwick.ac.uk, University Of Warwick Medical School Coventry CV4 4AL** *

Corresponding Author: Petra Hanson - Petra.hanson.1@warwick.ac.uk, University Of Warwick Medical School Coventry CV4 4AL *

Trial Sponsor: University of Warwick, Coventry, CV4 7AL, 024 7652 3523

Version/11.12.25

For peer review only - http://bmjopen.bmj.com/site/about/guidelines.xhtml

Abstract

Introduction: Obesity affects over a quarter of the UK population and can lead to serious health issues. NHS Specialist Weight Management Services offer treatments including lifestyle advice, psychological support, and medications, but access and availability vary by region. Although around 4 million people could be eligible for NHS Specialist Weight Management Services [WMS] annually, capacity is limited to 35,000, severely limiting overall access for those who need it. While digital technology has started to be used in WMS, more evidence is needed to confirm its long-term effectiveness, acceptability, and cost-effectiveness. This study explores the use of Gro Health W8Buddy, a digital platform and app providing remote Specialist WMS. It aims to determine the long-term health benefits of remote WMS pathway Gro Health W8Buddy compared to standard NHS WMS delivered in hospitals, and to improve patients access to services.

Methods and analysis: The study is a real-world evaluation with observational data collection. We will recruit 450 study participants from four NHS specialist WMS who will choose either standard NHS WMS or the digital pathway Gro Health W8Buddy. Participants are being given the option to choose their pathway to generate real world evidence. We will measure and analyse health outcomes including weight loss, time taken to be treated and cost-effectiveness, at 18 months and follow up at 24 months for later analysis [outside of this core funding]. We will gather experiential data from patients and healthcare professionals through surveys, observation, and interviews.

Ethics and dissemination: Ethical approval has been obtained from NHS Health Research Authority [HRA] and Health and Care Research Wales [HCRW] [Supplementary Figure 3] [REC reference: 25/EM/0147]. Our findings will be disseminated through academic publications, conference presentations and stakeholder engagement.

For peer review only - http://bmjopen.bmj.com/site/about/guidelines.xhtml

Strengths and limitations of this study:

- The study is structured around four distinct work packages, each employing a unique research methodology
- The digital intervention has been co-created with NHS staff and patients and is backed by research
- This research methodology has been shaped by PPI input from all four NHS sites
- The study methods enable real world evidence to be generated by allowing participants to choose their own pathway of care. Therefore, we will provide credible evidence for further roll out of digital technology in weight management services across the UK
- Allocation is not randomised which may lead to more participants in digital care or vice versa, this approach inevitably introduces complexity into the analysis, but it is a design choice advised by patient contributors.

For peer review only - http://bmjopen.bmj.com/site/about/guidelines.xhtml

Introduction

Background

In the UK, a quarter of adults live with obesity, with higher rates among people from Black and Asian ethnic groups and people living in the most deprived areas [1]. People living with obesity are at increased risk of chronic diseases including Type 2 Diabetes Mellitus [T2DM] and cardiovascular disease as well as at risk of shorter life expectancy [2]. Treatment of obesity cost the NHS £6.5bn in 2022, with an associated ill health annual full cost of £58bn [3]. The NHS and Office for Life Sciences [OLS] identified treatment and prevention of obesity as one of its healthcare missions [4].

A tiered approach to weight management exists in the UK. Tiers 1 and 2 are delivered in community and primary care. Tier 3 is delivered by specialist Weight Management Services [WMS] which are often based in a hospital setting. WMS comprise of multidisciplinary teams who provide dietetics, psychology, and consultation by specialist doctors. Tier 4 services include bariatric surgery [5]. In the UK, NICE recommends that people with BMI over 40 kg/m^2 or over 35 kg/m^2 with medical problems [e.g., T2DM, hypertension] are eligible for referral to WMS [6]. However, there is substantial geographical and service variation, with many areas lacking access to services [7].

Outcomes from traditional face-to-face WMS are mixed, with 50% of patients in most NHS WMS achieving a 5% weight loss over 6-12 months according to a recent review [8]. Retention rates are low [<55%] and there is little long-term [>12 months] data on sustainability of weight loss [8]. WMS have limited capacity for just 35,000 patients [9], yet 4 million people are eligible for services. This represents a significant capacity issue [10]. Due to these access constraints only 3.13% of this population receive a referral to WMS [9]. There is also marked regional variation in service design and accessibility [7]. High risk groups such as individuals from deprived areas in the UK often face barriers to accessing in

For peer review only - http://bmjopen.bmj.com/site/about/guidelines.xhtml

person Tier 3 services due to costs of transport and having support with childcare [11]. This is important to note as the obesity prevalence is 37.4% in deprived areas compared to 19.8% in less deprived areas [12].

In March 2023, NICE recommended the obesity medication semaglutide [Wegovy [TM]] for people accessing WMS [13]. Additionally, tirzpatide was recommended for obesity treatment from December 2024 [14]. These treatments represent a step change in Tier 3 WMS services and are part of the government-backed pilot to widen access to this medication with the use of digital technology [15]. However, guidance on the incorporation of digital technology to address how to safely incorporate the drugs remains unclear. We aim to fill the gap with this study.

Digital technology for managing chronic disease, including obesity, is expanding rapidly in the NHS [16]. Acceptability of digital technology for weight loss is high in both community and WMS settings, which is important to note considering the current barriers to WMS uptake and accessibility [17-20]. However, effectiveness varies. Evidence shows weight loss ranging from 13% at 4-month [digital + meal replacements] [21] to 7.6% at 12-month [digital + coaching] [22]. A community based digital weight management intervention recorded 5% weight loss at 2 years in 18% of users [20]. According to the evidence, there is a gap and need for further digital weight management research to investigate standard and digital care, alongside the integration of pharmacotherapy [e.g., semaglutide] and the use of a multi-disciplinary team [21]. Currently the evidence on the digital divide created by digital technology for weight management is lacking [23]. Therefore, innovative approaches are needed in the NHS WMS to reduce health inequality, address efficiency, increase capacity, and improve access to pharmacotherapy for obesity.

For peer review only - http://bmjopen.bmj.com/site/about/guidelines.xhtml

As a result of the above evidence, we co-created a digital support tool *Gro Health W8Buddy* [W8Buddy] with a digital company Diabetes Digital Media [DDM] to provide digital technology to deliver NHS WMS [7]. W8Buddy provides personalised WMS to support patients to achieve their self-selected health goals and support weight loss with real-time remote monitoring. The creation of W8Buddy was supported by an *HEE Topol Digital Fellowship* and *NHS Clinical Entrepreneur scheme* [24]. Evidence has shown that W8Buddy helps to reduce weight, improve glycaemic control and wellbeing among people living with obesity, overweight or with T2DM [25, 26]. W8Buddy was endorsed by *NICE Early Value Assessment* for Digital Technologies in Specialist Weight Management in October 2023 [27].

Our initial 1-year evaluation of 412 patients showed that 220 patients using W8Buddy [version 1 – information resource] *alongside* traditional WMS lost significantly more weight [0.75 kg/month] than 192 patients using traditional WMS [28]. Since then, W8Buddy has been adapted to provide a fully remote WMS. This study aims to provide longer term real-world evidence [RWE] of the clinical and cost-effectiveness of the adapted W8Buddy in the NHS.

Research question: What is the long-term [18-month] impact on clinical outcomes, including health-related quality of life [QoL] and cost-effectiveness of W8Buddy with and without weight management medication and lifestyle support to people living with obesity, compared with WMS standard care?

Objectives

1. To generate longitudinal evidence of the clinical and cost-effectiveness of the W8Buddy from four NHS WMS, University Hospital Coventry & Warwickshire [UHCW], University Hospitals Birmingham [UHB], London Luton Trust [LLT] and

For peer review only - http://bmjopen.bmj.com/site/about/guidelines.xhtml

Hywel Dda University Health Board Trust [HDUHBT], using the digital pathway W8Buddy.

2. To generate sufficient evidence to satisfy NICE guidance requirements [27].
3. To understand the process of implementation and use of W8Buddy within WMS.
4. To create a manual for safe incorporation of a digital pathway delivering weight management medication into existing WMS across the UK.
5. To make recommendations for how we can optimise existing infrastructure of WMS to improve ongoing real world evidence generation.

For peer review only

For peer review only - http://bmjopen.bmj.com/site/about/guidelines.xhtml

Methods

Study Design

Multi-site prospective comparative observational cohort study with embedded cost-effectiveness analysis and process evaluation. Comprising of four work packages [WP] to assess the following; 1] Set up, Patient and Public Involvement and Engagement [PPIE] & Stakeholder engagement, 2] Clinical impact, 3] Health economic impact and 4] Process evaluation.

Eligibility criteria to enter WMS in the UK

Inclusion criteria reflect the eligibility of people to enter the clinical services participating in this research, including services with additional eligibility criteria restrictions [subject to local processes that may differ slightly]. However, whilst individual services may have further restrictive criteria, which is reflective of many WMS all participants are included within:

- Adults aged 18 years and above
- Class 2 obesity [BMI $\geq$ 35kg/m^2] with at least 1 medical complication [e.g., prediabetes, type 2 diabetes [T2DM]]; or patients with obesity class 3 [BMI $\geq$ 40kg/m^2], without any complications.

The eligibility criteria for access to Tier 3 WMS for adults from South Asian, Chinese, other Asian, Middle Eastern, Black African, or African-Caribbean background have BMI cut offs 37.5 kg/m^2 without comorbidities.

Exclusion criteria:

For peer review only - http://bmjopen.bmj.com/site/about/guidelines.xhtml

- Pregnant or breastfeeding
- Type 1 diabetes mellitus
- End stage renal disease [eGFR<15, receiving renal replacement therapy]
- Active or untreated alcohol or drug dependency
- Decompensated liver disease
- History of anorexia, undergoing treatment or awaiting treatment for DSM-V classified eating disorder
- Severe mental health disease, including acute mental health crisis or self-harm in the last 12 months
- Unstable mental health disease including unstable personality disorder, schizophrenia, and bipolar disorder
- Unwilling or unable to commit to participation across the length of the research study.
- Patients targeting bariatric surgery within the length of the research study.
- Participating in another weight management research intervention
- Participating in the diabetes remission program

Study setting and sites

This study will run at four NHS sites [UHCW, UHB, LLT, HDUHBT]. Standard care differs across the recruiting sites, including the mixed provision of obesity medication. Sites without medication as standard care will be noted as the digital pathway *without medication.*

For peer review only - http://bmjopen.bmj.com/site/about/guidelines.xhtml

Patient and public involvement [PPI]

A W8Buddy PPI Group has been established with a minimum of four PPI Representatives with lived experience of WMS from each site. Potential PPI representatives have been identified through WMS teams at each participating site and obesity charities. Those interested in joining the groups are invited to meet with the PPIE Lead and/or UHCW PPIE Team to discuss the opportunity, commitment required, and support offered and to find out about their experience and background. PPI representatives selected represent the diverse population of the WMS users in each area. The Group meet quarterly to ensure ongoing input from people with lived experience, including identifying outcome measures that are meaningful for people with obesity, providing feedback on participant facing documents, working on creating local pathways and training materials. The PPI Group will also shape analysis and risk mitigation, may take part in conducting semi-structured interviews as part of qualitative research, and support dissemination of findings afterwards. This research methodology has been shaped by PPI input from all four NHS sites.

Sample size calculation

We will collect weight data at baseline and a minimum of three occasions during the study, for both pathways. Powering the study to show that the digital pathway is non-inferior to the standard of care pathway, with a difference less than 4kg, at 90% power and a two-sided 5% level, gives a sample size of approximately 180 participants. Further assuming a correlation between the repeated weight measurements in the range 0.2 to 0.5, which previously collected data suggests is reasonable [29], based on a longitudinal mixed model/repeated measures ANCOVA gives a total sample size of 360. Conservatively assuming 20% loss during follow-up, the target sample size is 450.

For peer review only - http://bmjopen.bmj.com/site/about/guidelines.xhtml

Given the current widespread use of the digital [W8Buddy] intervention at the lead centre [UHCW], the control [standard of care; SC] intervention at this site will be different from that offered at the other sites. For this reason, we plan to characterise the control intervention at UHCW as a "hybrid" digital intervention. We will aim to recruit sufficient participants [i.e. 360] at the three other sites [UHB, LLT, HDUHB] to fully power the study for the planned comparison between the digital and standard of care groups. Additionally, we will collect data from the "hybrid" digital intervention at UHCW to augment and further refine the primary analysis.

Screening, recruitment, and consent

Potential participants will be identified by research nurses once they have been referred [or self-referred] to the local Tier 3 WMS. Participants will be screened electronically against pre-specified criteria.

Eligible participants will be able to choose how they receive their WMS - either in the standard NHS delivery, or via fully digital platform Gro Health W8Buddy. Site specific standard of care pathways will be delivered by the usual health care professionals, employed by each NHS site. The digital pathway will be delivered by health care professionals employed by DDM [providers of Gro Health W8Buddy]. We aim to recruit a total of 450 participants across both arms from September 2025 to February 2026.

Those who satisfy the study inclusion criteria will be contacted by the research team via telephone. Brief information about the study will be provided to potential participants, including a link to a website with information about the study. This website will be password protected as only eligible participants will be able to view it. Digital information sheets and consent forms will be hosted on the website.

For peer review only - http://bmjopen.bmj.com/site/about/guidelines.xhtml

If a patient is unable to access the website [therefore, unable to complete electronic consent] the local site research nurse will invite them to the hospital. They will show the participant the research website and help them with electronic consent. Later, the research nurse will print out the consent form [signed copy] only for the patient if they do not have access to email.

Electronic consent for data collection, with an option to consent for ongoing data collection, will be obtained. Following informed consent, participants will be able to choose the digital or standard care pathway via the website. A copy of the signed consent form will be sent to the participants, local research team and site Principal Investigator [PI].

To support data collection in the standard care pathway, participants will be offered a £10 voucher for each completed survey at month 0, 6, 12, 18 and 24; vouchers will not be provided for sign up only, surveys must be completed by participants.

Intervention Gro Health W8Buddy

Gro Health W8Buddy is a virtual specialist WMS pathway. It is accessed through a digital behavioural change platform called the Gro Health app [see, Figure 1]. Participants on the W8Buddy digital arm will receive 52 weeks of support from health care professionals employed by DDM. At week 52 each participant on the digital pathway will be assessed and the following options offered: discharge to primary care, onward referral to local Tier 4 if interested in surgery, onward referral for local Tier 3 MDT discussion, extension of digital pathway by 6-months, referral to local Tier 2 weight management service.

Figure 1: Gro Health W8Buddy platform and app – description and visual representation of the W8Buddy platform in an app format

For peer review only - http://bmjopen.bmj.com/site/about/guidelines.xhtml

For peer review only

Standard care

Participants in the standard care arm will receive the Tier 3 WMS that is delivered by their local NHS trust. All four NHS trusts included in the research have differences in their Tier 3 service, which will be explored in detail as part of the study. The differences range from the amount of input from healthcare professionals [psychologist, dietitian, physician], modality of sessions [1-1 or group sessions] and frequency. Post study care for participants in standard care will be delivered in accordance to their NHS trust policies for Tier 3 weight management. As discussed previously, the W8Buddy intervention is included in the standard of care at the lead centre [UHCW]. It is used as a digital support tool only [no access to clinicians via W8Buddy], enhancing the usual face to face care, and as such, differs from entirely remote digital pathway. For this reason, the model of delivery of care is hybrid at UHCW [refer to table 1 for further detail].

Table 1. Standard of care

NHS Site	Standard of care	Weight management medication offered in current tier 3 service* *please note this information is accurate at time of publication*
UHCW	Hybrid standard of care with digital W8Buddy input and in person MDT support. Mixture of one-to-one and group sessions delivered by dietitians, psychologists, and consultant endocrinologists.	No
UHB	Mixture of group sessions and one-to-one appointments delivered by dietitians, psychologists and consultant endocrinologists following a face-to-face introductory group session.	No

For peer review only - http://bmjopen.bmj.com/site/about/guidelines.xhtml

LLT	Mixture of face to face and group sessions delivered by psychologists, dietitians and consultant endocrinologist.	No
HDUHBT	Mixture of face to face and group sessions delivered by psychologists and dietitians. After completion they are seen by consultant.	Yes

Figure 2: Participant flow chart, the flow chart describes where participants will be providing data in both the digital and standard of care arm.

Assessment and management of risk

Risks relate to data management/reporting across four sites and management of the digital pathway delivered by DDM. The latter is assured by DDM. Table 2 lists these. Further information on data management and flow can be found in supplementary material table 1 (ST1), adverse events (ST2) and supplementary figure 1 (SF1).

Table 2 Assessment and management of risk

Data reporting	Not reporting data by participants, mitigated by regular support via the app or contact from research team if not completing surveys.
Data protection	As with any online service proper data handling is assured by high security data storage in line with NHS Digital policies. Data from NHS sites transferred to analytical partner using secure pathways.
Delivery	The site research team will oversee all participants, including those accessing digital pathway. The first contact for any queries for participants on digital pathway will be the digital provider, and if the query is not resolved the research site team will be contacted. The project will be managed by PH and AG via weekly WP meetings between sites and project manager, with monthly oversight by each site PI. Three-monthly meetings of PPIE group will ensure ongoing input from patients. Annual project steering committee meetings will review risk registers.
Imbalanced recruitment	More patients opting for digital rather than standard of care pathway [or vice-versa], mitigated by over-recruiting standard of care [or digital] patients and adjusting as per analysis plan using propensity-weighted approach, purposive sampling and ongoing monitoring of recruitment, communication to sites, telephone and offline packs as another option for those choosing digital pathway.

For peer review only

For peer review only - http://bmjopen.bmj.com/site/about/guidelines.xhtml

Engagement with the digital pathway	Low engagement from high-risk/target groups resulting in lower enrolments and participation from those most in need of the service. Mitigated by patient friendly resources, onboarding support, research nurse at each site to act as a point of call.
Patient safety	Without face-to-face contact, healthcare professionals may miss important physical signs and symptoms that might indicate a health concern for study participants. Participants might also misunderstand or misuse instructions regarding their medication, increasing the risk of side effects or adverse events. This is mitigated through symptom tracking, face-to-face videoconferencing/virtual calls, identifying signs of body language, and recording sessions for audit to ensure ongoing patient safety. Digital phenotyping of patients occurs in real-time, and any thresholds of anxiety or depression are noted in the clinical dashboard and the clinical team are notified. Omni-directional communication through the platform allows clinicians to engage directly with patients and vice-versa with up-to-date, real-time data. Patient expectations are managed by informing the patient if their coach is offline and encouraging them to seek emergency medical assistance if at risk.
Medication safety on digital pathway	Prescribing medication remotely requires a comprehensive understanding of the patient's current health status, medications, and lifestyle, which can be challenging to obtain in a virtual environment. There is also the risk of potential drug interactions that might be overlooked without a detailed in-person evaluation. This is mitigated through patient completion of psychological assessments, medication eligibility assessment, and approval for medication from the multidisciplinary team [MDT] team providing the patient's care. Regular reviews ensure medication is not mismanaged and appropriate for the patient. Patient is provided with videos and resources on how to inject.
Psychological safety on digital pathway	Weight management is closely tied to self-esteem and body image, which can be negatively impacted if the focus is solely on weight loss. Additionally, the digital nature of virtual services can exacerbate feelings of isolation and lack of support. This is mitigated through support from a psychotherapist/psychologist throughout the programme, access to private/group coaching for on-demand support, and artificial intelligence [AI] algorithms that provide digital phenotype measurements for a patient's likelihood of anxiety, stress, and depression based on conversations/engagements in the community.
Eating disorders	Weight loss can trigger or exacerbate eating disorders. For individuals with a history of eating disorders, weight loss medications could potentially be misused in a harmful way. This is mitigated through patient completion of psychological assessments, medication eligibility

For peer review only

For peer review only - http://bmjopen.bmj.com/site/about/guidelines.xhtml

	assessment, and approval for medication from the MDT team providing the patient's care. On the digital pathway, regular reviews with a pharmacist and psychologist ensure medication is not mismanaged and appropriate for the patient. Signposting and referral back to the GP will be provided to any patients who display signs of disordered eating. On the standard of care pathway, local protocols will be followed.
Engagement with the standard of care pathway	High drop out from the standard of care pathway: there is a high drop out from the standard of care service in Tier 3 Weight Management, resulting in high loss to follow up. This will be mitigated by having a dedicated research nurse who will contact patients to ensure they complete all data collection in timely fashion. Additionally, participants in the standard of care will receive vouchers to compensate them for the additional surveys they will be asked to do.

Outcomes

The primary outcome is changes in body weight after 18-months of using either [i] a fully digital weight management programme [remote digital pathway Gro Health W8Buddy] or [ii] site-specific delivered weight management programmes incorporating face to face and/or virtual group sessions [standard care].

Secondary outcomes are metabolic markers [HBA1c, lipids], blood pressure, waist circumference and health related quality of life [EQ-5D-5L] [30].

For peer review only

Data collection

In Supplementary Table 4 (ST4) we detail what baseline and follow up data will be collected to assess primary and secondary outcomes. During the economic evaluation, additional cost, and process evaluation qualitative data will be collected.

For peer review only

For peer review only - http://bmjopen.bmj.com/site/about/guidelines.xhtml

Monitoring

The study will be monitored by the study management group and the University of Warwick representative of the Sponsor to ensure that the study is being conducted as per protocol, adhering to Research Governance and Good Clinical Practice [GCP]. Central monitoring activities will be performed such as data quality checks. The recruiting site is obliged to assist the sponsor in monitoring the study. These may include hosting site visits, providing information for remote monitoring, or putting procedures in place to monitor the study internally. The approach to, and extent of, monitoring will be specified in a study monitoring plan determined by the risk assessment undertaken prior to the start of the study.

Analysis

Statistical analysis plan A detailed statistical analysis plan will be prepared by the statisticians, and agreed with the study team, prior to final analysis, and which will follow the NICE RWE Framework [28]. Comparisons will be primarily on an as-started [intention to treat, ITT] basis. We do not expect any or only minor levels of treatment switching or discontinuation for the W8Buddy group. Therefore, we would expect the on-treatment and as-started treatment effect estimates to be equivalent in this population. We will report the result according to the STROBE guideline [29].

Summary of baseline data Demographic and baseline data will be summarised overall and by treatment arms and sites. Categorical data will be summarised as count and percentage, and continuous data will be summarised as number of missing data, mean with standard deviation, and median with 25th and 75th percentiles.

For peer review only - http://bmjopen.bmj.com/site/about/guidelines.xhtml

Missing data Patterns of missing data/discontinuation will be reported and assessed if missingness was at random or provided useful information on treatment efficacy.

Primary and secondary outcome analysis The primary analysis will compare temporal changes in weight for the W8Buddy and standard care group, from baseline to completion of follow-up [at 18 months], using longitudinal mixed effects regression models. The effectiveness of the W8Buddy will be quantified by differences in rates of weight change compared to standard of care during follow-up. All the secondary outcomes will be analysed individually with longitudinal mixed effects models.

For all analyses, we will consider adjusting for a range of demographics and clinical characteristics [e.g. age, gender, ethnicity, Index of Multiple Deprivation/Welsh Index of Multiple Deprivation, comorbidities, NHS site, mode and distance of travel and other confounders identified using Directed Acyclic Graphs with stakeholders PPI members and members of the steering committee] [33] using propensity-weighted longitudinal mixed models. We expect the fixed effects to be demographics and clinical characteristics as mentioned above and the random effects to be the sites and individuals. A sensitivity analysis using *distance to NHS site* as an instrumental variable [34] will also be conducted to assess the assumption of no unmeasured confounders.

The comparison between the hybrid intervention and both the standard of care and fully digital intervention will be an unpowered secondary analysis, that we hope will offer additional insights and help to characterise the magnitude of intervention effects from the primary analysis.

Economic evaluation

For peer review only - http://bmjopen.bmj.com/site/about/guidelines.xhtml

We will develop a costing toolkit to provide transparent resource use and costs for the delivery of the standard care and digital delivery pathways. The toolkit development process will comprise three stages: planning, data collection, synthesis and analysis [35, 36]. We will require resource use and cost estimates for delivery of W8Buddy providing weight management medication and standard of care [Tier 3 WMS]. We will produce a systematic review of cost effectiveness literature to inform the development of a decision-analytic model. The model will be used to estimate the cost-effectiveness of Tier 3 WMS in addition to W8Buddy compared to weight management services delivering weight management medication to support people with obesity. This will be fundamental to help to understand the long-term cost-effectiveness of weight management programmes as they allow for the extrapolation of the short-term benefits of interventions.

Long-term follow-up analysis Outcomes collected beyond the 18-month primary outcome will be analysed at a later stage, subject to further funding. The aim of the longer term follow-up is to explore the long-term impact of digital weight management interventions. We expect the analyses will be done similarly to our primary and secondary clinical, and health economic analyses.

Process Evaluation

A mixed methods process evaluation will be conducted to understand the implementation of W8Buddy. Process evaluation data is listed in Supplementary Table 4.

Electronic data from intervention and control pathways: We will collate data from the electronic clinical records, intervention, and study records of all study participants data on:

For peer review only

For peer review only - http://bmjopen.bmj.com/site/about/guidelines.xhtml

- Fidelity: delivery of coaching sessions, check-ins and physical activity sessions and progress reviews
- Adherence: Including biomedical explanations of adherence [comorbidities], we will assess user adherence to each consultation in the digital pathway [a patient attending 75% of consultations or more is considered adherent]
- Who chooses the digital pathway or not
- Who engages with the digital pathway, their level of engagement and predictors of engagement

Qualitative data collection and analysis

We will record observation data as field notes and collate documents. Qualitative data will be obtained from participants interviews via telephone or videoconference. We will audio record the interviews, transcribe using software and check the transcripts against the audio recording. We will undertake thematic analysis, with independent quality checks. [29]. This analysis will be undertaken independently of the quantitative analysis team.

Initial implementation and health professional experience of delivering digital pathway

We will observe site team meetings and implementation of the intervention, access team documents and undertake interviews with team members implementing the digital pathway to understand how they are meeting implementation challenges, training delivered, and quality assurance processes put in place. During the observations, the patient will be asked for permission for the researcher to remain present during the health professional-patient interaction. If this is refused, the researcher will leave.

For peer review only - http://bmjopen.bmj.com/site/about/guidelines.xhtml

In months 12 and 18 we will interview those delivering the digital pathway to understand their experiences, how they adapt WMS and any unexpected pathways or consequences [estimate 5/site]. We will use implementation frameworks [37] to guide thematic analysis of implementation data whilst remaining alert to new themes. Early analysis of data on implementation will be used to provide formative feedback for subsequent intervention site teams.

User experience of digital pathway

In semi-structured interviews with users, we will explore their experiences of engaging with the digital pathway, influences on their engagement, challenges and how these were met, adaptations, impact, and mechanisms of impact [estimate 10/site, total 40].

We will undertake brief structured interviews with participants not choosing the digital pathway [5/site] and participants not adherent to the digital pathway and/or medicine [5/site]. We will ask participants why they did not choose the digital pathway/drop out or return to the digital pathway/medicine. We will develop our brief structured questions with input from the study PPI group to ensure neutral, non-judgemental phrasing. We will sample participants consecutively across study sites and approach them for recruitment at least a week after their decision.

Describing the control intervention patient pathway

A researcher will visit each site to observe the control intervention. The team providing the control intervention will provide a written description of the control intervention patient pathway. From observation and conversation, the researcher will add additional details and variation on that pathway. The study team will analyse the observation notes to compile a description of the control intervention and check this with staff delivering the control

For peer review only - http://bmjopen.bmj.com/site/about/guidelines.xhtml

intervention. Study sites will extract from the appointment system and relevant clinical records of their control intervention pathway, patient adherence data in terms of attendance at appointments, whether they are individual or group and duration of appointments, and use of medication.

Ethics and dissemination Ethical approval for this study has been obtained from the Health Research Authority [HRA] Research Ethics Committee [REC] and Health and Care Research Wales [HCRW] [REC reference: 25/EM/0147][Supplementary Figure 3]. Informed consent will be required to enter the study, as well as to share anonymised data with local health care team and study team. Participants will be consented for long term follow-up using routinely collected data. Protocol amendments [if any] will be reported and discussed with National Institute for Health and Care Research [NIHR] and local ethics committee.

Study data will be collected on the screening logs, W8Buddy App, and validated questionnaires [see Supplementary Table 4] electronically. Health record data for each site will be accessed by local research team. An Electronic Data Capture system will be used to record and store study data transferred securely from the W8Buddy App and hospital medical records. This will be the main Study Database. Only research team and members of the DDM team will have access to it. They will be listed in the delegation log and must hold Good Clinical Practice [GCP] certification. Audio recordings of interviews will be stored on secure University of Warwick computers and accessed only by research team located at Warwick.

We have a tiered approach to dissemination to ensure our findings reach participants, policy makers and practitioners. We will host a stakeholder engagement dissemination event at the end of the study to maximise the reach of our finding.

For peer review only

For peer review only - http://bmjopen.bmj.com/site/about/guidelines.xhtml

Our results will inform UK guidelines [incorporation of digital tool delivering pharmacotherapy into obesity management guideline]. Academic publications will include protocol paper, main results of RWE evaluation, the health economic assessment and a paper describing the scale-up in NHS services and impact on multiple long-term conditions. We will create NHS business case, build on ongoing partnership developed across four adoption sites, produce equality and health inequality impact assessment, and have evidence for health and financial impact to support NHS adoption. We will improve NHS services by facilitating the testing and scale-up of our digital innovation which can support other Trusts wanting to deliver digitally enabled services. This will include recommendations to other service providers, targeted education, and creation of a virtual learning environment [hosted by an establish Warwick University platform]. Our study outputs will provide evidence to apply for a NICE review of clinical guidelines and an update to NHS reimbursement criteria, which will open the door to national adoption by weight management services.

For peer review only

Discussion

Research is needed to determine how we can most effectively deliver WMS to more people [21]. One potential solution to address issues of patient access is the introduction of digital services [14-18]. However, long term evidence about clinical and cost-effectiveness is

For peer review only - http://bmjopen.bmj.com/site/about/guidelines.xhtml

required before digital pathways are fully integrated into NHS WMS across the whole country.

In this study we aim to provide longer term real-world evidence [RWE] of the clinical and cost-effectiveness, as well as barriers and facilitators of uptake of W8Buddy digital pathway implemented in four NHS sites. All these potential findings are essential considering the current data on the demand for WMS, as well as the health and economic impact of obesity [2,3]. Guidance on the incorporation of weight management drugs alongside digital interventions is also an unclear gap we intend to fill with our research. With capturing the variation that exists amongst our four NHS sites, we will provide guidance on implementation into further NHS sites.

Allowing study participants to choose their own pathway will allow RWE to understand uptake of the digital pathway when incorporated into standard care. Furthermore, we are assessing whether W8Buddy can decrease NHS waiting times and costs. Individuals living with obesity could in time be supported virtually without the need to attend in person specialist services with the associated costs and challenges of access. Investigating pathways, waiting times and accessibility is important for Obesity research considering the low retention rates and high demand but low availability in WMS [7,11,12]. The use of a digital pathway will also provide further insight for innovative diagnostic pathways for obesity management, such as moving from acute to home care and being offered to more patients in a cost-effective way, which would further contribute to the literature base.

We acknowledge the challenges that may occur with the adoption of a digital pathway into NHS sites such as recruitment rates, levels of engagement and differences in WMS at NHS sites. However, capturing any challenges that may arise and providing solutions will benefit the future implementation of W8Buddy and contribute to generalisable knowledge. Our

For peer review only - http://bmjopen.bmj.com/site/about/guidelines.xhtml

design means that we will not have matched cohorts across treatment arms, meaning both known confounders [e.g. age, BMI] and unknown factors may influence outcomes. To address this, our planned analyses will incorporate statistical adjustments to account for these differences as far as possible. We recognise the limitations of this approach, but it is consistent with the pragmatic design and will allow us to report findings that are representative of real-world practice.

We aim to improve NHS Specialist WMS services by facilitating the evidence generation and scale-up of digital innovation Gro Health W8Buddy. This could in turn support other NHS organisations wanting to deliver digitally enabled services. The findings of this study will be used to generate recommendations for other service providers, targeted education, and creation of a virtual learning environment. It is aimed the study outputs will provide evidence needed for full NICE technology appraisal, as well as evidence for NHS commissioners to facilitate national adoption of technology in specialist WMS.

For peer review only

For peer review only - http://bmjopen.bmj.com/site/about/guidelines.xhtml

References

1. Baker C. Obesity statistics 2023 [Available from: https://researchbriefings.files.parliament.uk/documents/SN03336/SN03336.pdf.
2. Peeters A, Barendregt JJ, Willekens F, Mackenbach JP, Al Mamun A, Bonneux L. Obesity in adulthood and its consequences for life expectancy: a life-table analysis. Ann Intern Med. 2003;138[1]:24-32.
3. Economics F. Estimating the full costs of obesity 2022 [Available from: https://www.frontier-economics.com/media/hgwd4e4a/the-full-cost-of-obesity-in-the-uk.pdf.
4. Sciences OfL. Life Sciences Vision, Build Back Better: our plan for growth 2021 [Availablefrom:https://assets.publishing.service.gov.uk/media/612763b4e90e0705437230c3/life-sciences-vision-2021.pdf]
5. NHSEngland. Appendix 9 Guidance for Clinical Commissioning Groups [CCGs]: Service Specification Guidance for Obesity Surgery 2016 [Available from: https://www.england.nhs.uk/wp-content/uploads/2016/05/appndx-9-serv-spec-ccg-guid.pdf.

For peer review only - http://bmjopen.bmj.com/site/about/guidelines.xhtml

6. NICE. Obesity: Identification, assessment and management 2014 [Available from: https://www.nice.org.uk/guidance/cg189/chapter/1-Recommendationsassessment.

7. Elhariry M, Iyer P, Isack N, Sousa B, Singh P, Abbott S, Wiggins T, Nirantharakumar K, Bellary S, Flint SW, Pournaras DJ, Hazlehurst JM. Variation in the commissioning of specialist weight management services and bariatric surgery across England: Results of a freedom of information-based mapping exercise across the 42 integrated Care Systems of England. Clin Obes. 2025 Jun;15[3]:e12731. doi: 10.1111/cob.12731. Epub 2025 Jan 19. PMID: 39828897; PMCID: PMC12096057.

8. Hazlehurst JM, Logue J, Parretti HM, Abbott S, Brown A, Pournaras DJ, et al. Developing Integrated Clinical Pathways for the Management of Clinically Severe Adult Obesity: a Critique of NHS England Policy. Curr Obes Rep. 2020;9[4]:530-43.

9. GOV.UK. New Drugs pilot to tackle obesity and cut NHS waiting list 2023 [Available from: https://www.gov.uk/government/news/new-drugs-pilot-to-tackle-obesity-and-cut-nhs-waiting-lists.

10. Desogus D, Menon V, Singhal R, Oyebode O. An Examination of Who Is Eligible and Who Is Receiving Bariatric Surgery in England: Secondary Analysis of the Health Survey for England Dataset. Obes Surg. 2019;29[10]:3246-51.

11. Office for Health Improvement and Disparities. Obesity Profile: Short Statistical Commentary – May 2025 [Available from:https://www.gov.uk/government/statistics/obesity-profile-may-2025-update/obesity-profile-short-statistical-commentary-may-2025].

12. Public Health England. Weight Management Services: National Mapping Exercise Report 2017[https://assets.publishing.service.gov.uk/media/5a7f74d2e5274a2e8ab4c4b1/Final_Weight_Management_Mapping_Report.pdf].

For peer review only

For peer review only - http://bmjopen.bmj.com/site/about/guidelines.xhtml

13. NICE. NICE recommended weight-loss drug to be made available in specialist NHS services 2023 [Available from: https://www.nice.org.uk/news/articles/nice-recommended-weight-loss-drug-to-be-made-available-in-specialist-nhs-services.

14. NICE. Tirzepatide for managing overweight and obesity [ID6179] 2024 [Available from: https://www.nice.org.uk/guidance/indevelopment/gid-ta11156.

15. Centre DoHaSCM. Accessing Wegovy for weight loss: everything you need to know. 2023 [Available from: https://healthmedia.blog.gov.uk/2023/09/04/accessing-wegovy-for-weight-loss-everything-you-need-to-know/.

16. Seixas AA, Olaye IM, Wall SP, Dunn P. Optimizing Healthcare Through Digital Health and Wellness Solutions to Meet the Needs of Patients With Chronic Disease During the COVID-19 Era. Front Public Health. 2021;9:667654.

17. Batsis JA, Petersen CL, Clark MM, Cook SB, Kotz D, Gooding TL, et al. Feasibility and acceptability of a technology-based, rural weight management intervention in older adults with obesity. BMC Geriatr. 2021;21[1]:44.

18. Huntriss R, Haines M, Jones L, Mulligan D. A service evaluation exploring the effectiveness of a locally commissioned tier 3 weight management programme offering face-to-face, telephone and digital dietetic support. Clin Obes. 2021;11[3]:e12444.

19. Hanson P, Summers C, Panesar A, Oduro-Donkor D, Lange M, Menon V, et al. Low Carb Program Health App Within a Hospital-Based Obesity Setting: Observational Service Evaluation. JMIR Form Res. 2021;5[9]:e29110.

20. Richards R, Wren G, Whitman M. The Potential of a Digital Weight Management Program to Support Specialist Weight Management Services in the UK National Health Service: Retrospective Analysis. JMIR Diabetes. 2024;9:e52987.

For peer review only - http://bmjopen.bmj.com/site/about/guidelines.xhtml

21. Senecal C, Collazo-Clavell M, Larrabee BR, de Andrade M, Lin W, Chen B, et al. A digital health weight-loss intervention in severe obesity. Digit Health. 2020;6:2055207620910279.

22. Silberman JM, Kaur M, Sletteland J, Venkatesan A. Outcomes in a digital weight management intervention with one-on-one health coaching. PLoS One. 2020;15[4]:e0232221.

23. Szinay D, Forbes CC, Busse H, DeSmet A, Smit ES, König LM. Is the uptake, engagement, and effectiveness of exclusively mobile interventions for the promotion of weight-related behaviors equal for all? A systematic review. Obes Rev. 2023;24[3]:e13542.

24. NHS. NHS Clinical Entrepreneur 2022 [Available from: https://nhscep.com/2022/07/04/our-entrepreneurs-petra-hanson/.

25. Saslow LR, Summers C, Aikens JE, Unwin DJ. Outcomes of a Digitally Delivered Low-Carbohydrate Type 2 Diabetes Self-Management Program: 1-Year Results of a Single-Arm Longitudinal Study. JMIR Diabetes. 2018;3[3]:e12.

26. Abdelhameed F, Pearson E, Parsons N, Barber TM, Panesar A, Summers C, et al. Health Outcomes Following Engagement With a Digital Health Tool Among People With Prediabetes and Type 2 Diabetes: Prospective Evaluation Study. JMIR Diabetes. 2023;8:e47224.

27. NICE. Digital technologies for delivering specialist weight-management services to manage weight-management medicine: early value assessment 2023 [Available from: https://www.nice.org.uk/guidance/hte14/resources/digital-technologies-for-delivering-specialist-weightmanagement-services-to-manage-weightmanagement-medicine-early-value-assessment-pdf-50261971277509.

For peer review only

For peer review only - http://bmjopen.bmj.com/site/about/guidelines.xhtml

28. NICE. NICE real-world evidence framework 2022 [Available from: https://www.nice.org.uk/corporate/ecd9/chapter/overview.

29. Ghaferi AA, Schwartz TA, Pawlik TM. STROBE Reporting Guidelines for Observational Studies. JAMA Surgery. 2021;156[6]:577.

30. Hanson P, Abdelhameed F, Sahir M, Parsons N, Panesar A, de la Fosse M, Summers C, Kaura A, Randeva H, Menon V, Barber T Evaluation of the Digital Support Tool Gro Health W8Buddy as Part of Tier 3 Weight Management Service: Observational Study J Med Internet Res 2025;27:e62661URL: https://www.jmir.org/2025/1/e62661 DOI: 10.2196/62661

31. Braun V, Clarke V. Can I use TA? Should I use TA? Should I not use TA? Comparing reflexive thematic analysis and other pattern-based qualitative analytic approaches. Counselling and Psychotherapy Research. 2021;21[1]:37-47.

32. Hernández Alava M, Wailoo A, Pudney S, Gray L, Manca A. Mapping clinical outcomes to generic preference-based outcome measures: development and comparison of methods. Health Technol Assess. 2020 Jun;24[34]:1-68. doi: 10.3310/hta24340. PMID: 32613941; PMCID: PMC7357250.

33. Tennant PWG, Murray EJ, Arnold KF, Berrie L, Fox MP, Gadd SC, et al. Use of directed acyclic graphs [DAGs] to identify confounders in applied health research: review and recommendations. Int J Epidemiol. 2021;50[2]:620-32.

34. Greenland S. An introduction to instrumental variables for epidemiologists. Int J Epidemiol. 2000;29[4]:722-9.

35. Anderson DM, Wren Tracy J, Cronk R, Schram H, Behnke N, Bartram J. A toolkit for costing environmental health services in healthcare facilities. J Water Sanit Hyg Dev. 2021;11[4]:668-75.

For peer review only - http://bmjopen.bmj.com/site/about/guidelines.xhtml

36. Boardman F. Reevaluating the remote: a realist evaluation of the practical, epistemological and ethical status of remote qualitative data collection in health. Funder MRC [Available from: https://gtr.ukri.org/projects?ref=MR%2FW021161%2F1.

37. Damschroder LJ, Aron DC, Keith RE, Kirsh SR, Alexander JA, Lowery JC. Fostering implementation of health services research findings into practice: a consolidated framework for advancing implementation science. Implement Sci. 2009;4:50.

Figure 1: Gro Health W8Buddy platform and app – description and visual representation of the W8Buddy platform in an app format

Figure 2: Participant flow chart, the flow chart describes where participants will be providing data in both the digital and standard of care arm.

For peer review only

For peer review only - http://bmjopen.bmj.com/site/about/guidelines.xhtml

Author's contributions

Petra Hanson [chief investigator and guarantor] initiated the research project, wrote the grant application and developed protocol, Amy Grove [chief investigator] initiated the research project wrote the grant application and developed protocol.

Emma Scott and Marie Thorpe [project managers] supported with writing grant application, securing funding, and developing the protocol.

Pranay Singh Deo [author] and Menxi Zhang [post-doctoral researchers] supported writing the protocol and are involved in conducting and analysing the research.

Manreet Thind, Keith Abrahams, Peter Auguste, Frances Griffiths, Amit Kaura, Nick Parsons and Siew Wan Hee [all co-applicants] are all involved in the methodology and analysis of the research, they also supported developing the protocol and grant applications.

Tracy Gazeley, Richard Green and Sarah O'Toole [co-applicants] supported with PPI involvement, supported with grant applications and writing the protocol.

Thomas Barber, Jonathan Hazlehurst, Akhila Mallipedhi and Anjali Zalin [co-applicants] all site principal investigators, supported with grant application and protocol.

Arjun Panesar and Charlotte Summers [co-applicants] supported with developing the protocol and producing the W8Buddy digital app.

Competing Interests Statement

- Dr Petra Hanson led the co-creation of W8Buddy on behalf of UHCW. She is not a stakeholder in DDM or does not have any employment contract with DDM. She is a named inventor at UHCW for W8Buddy. She has received honoraria for delivering talks from Sanofi, AstraZeneca and Royal College of Emergency Medicine.

For peer review only

For peer review only - http://bmjopen.bmj.com/site/about/guidelines.xhtml

- Dr Thomas M Barber has received funding for investigator-initiated research from Bayer, AstraZeneca, Shire and TDeltaS, and honoraria for educational talks and attendance at advisory board meetings for Sanofi, Novo Nordisk, Astrazeneca, Boehringer Ingelheim, MSD, Lilly and Napp.
- Prof Keith Abrams is a member of the National Institute for Health and Care Excellence [NICE] Diagnostics Advisory Committee, the NICE Decision and Technical Support Units, and is a National Institute for Health Research [NIHR] Senior Investigator Emeritus [NF-SI-0512-10159]. He has served as a paid consultant, providing unrelated methodological and strategic advice, to the pharmaceutical and life sciences industry generally, as well as to DHSC/NICE, and has received unrelated research funding from Association of the British Pharmaceutical Industry [ABPI], European Federation of Pharmaceutical Industries & Associations [EFPIA], Pfizer, Sanofi and Swiss Precision Diagnostics/Clearblue. He has also received course fees from ABPI and the University of Bristol, and is a Partner and Director of Visible Analytics Limited, a health technology assessment consultancy company.
- Arjun Panesar is the CEO and Head of AI and Ethics at DDM Health, Honorary Associate Professor at Warwick Medical School, University of Warwick, and serve as an Advisor to the Information School at the University of Sheffield, EPSRC Future Blood Testing Network, Science for Africa Foundation, NNEdPro Global Centre for Nutrition and Health in Cambridge and mentor at the Global Business School for Health at University College London. He is actively involved in the development and commercialisation of AI-based tools for digital screening, behaviour change and self-management.
- Dr Amit Kaura is the Chief Medical Officer for DDM Health but his involvement in the project is as an academic at Imperial College London. DDM Health will not be

For peer review only

paying Dr Kaura for his involvement, nor will they receive any project funds for his involvement.

- Dr Jonathan M. Hazlehurst declares advisory work previously for Novo Nordisk as well as honoraria for speaking engagements from Novo Nordisk and Eli Lilly and support for attendance at academic meetings from Novo Nordisk as well as Eli Lilly as well as academic funding from the NIHR.
- No other members of the research team have declared any conflicts of interest.

Patient and Public involvement [PPI]

PPI were involved in the design of the research, please refer to the methods section for further information

Funding Statement

This study/project is funded by the NIHR [grant number NIHR208100 https://fundingawards.nihr.ac.uk/award/NIHR208100]. The views expressed are those of the author[s] and not necessarily those of the NIHR or the Department of Health and Social Care.

Role and responsibilities of the Sponsor

The University of Warwick will act as study sponsor. The study will be conducted in accordance with Sponsor's Standard Operating procedures. The University of Warwick has a specialist insurance policy in place which would operate in the event of any participant suffering harm as a result of their involvement in the study Zurich Municipal Insurance. NHS indemnity operates in respect of the clinical treatment that is provided. Further information can be found in supplementary table 3.

For peer review only - http://bmjopen.bmj.com/site/about/guidelines.xhtml

Role and responsibilities of the Funder

This study is funded by the National Institute for Health and Care Research Invention for Innovation [NIHR i4i] programme [NIHR208100]. Supplementary figure 2 displays the design and management of the trial are entirely independent of the funder.

Data Availability Statement

Included in supplementary material table 1.

For peer review only

For peer review only - http://bmjopen.bmj.com/site/about/guidelines.xhtml

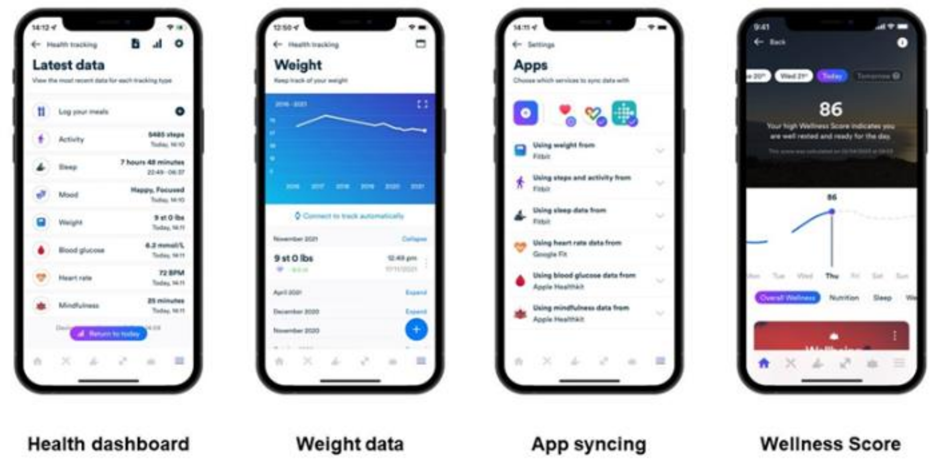

For peer review only

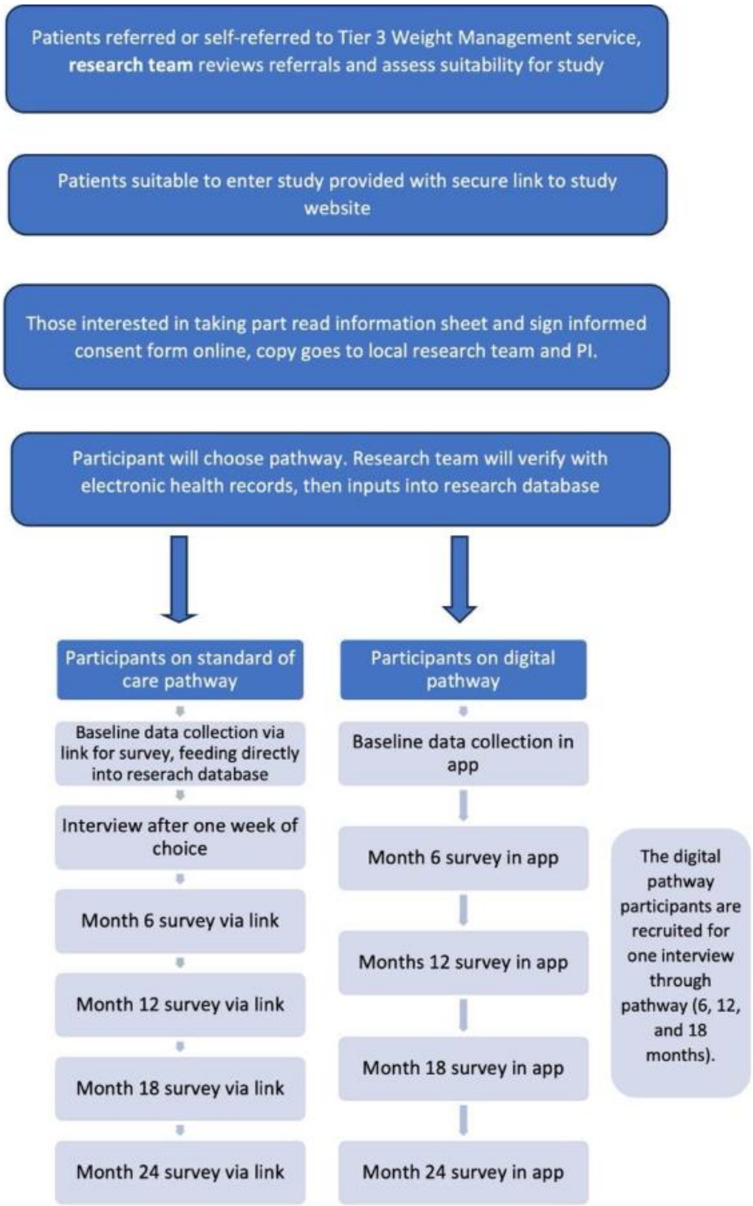
Patients referred or self-referred to Tier 3 Weight Management service, research team reviews referrals and assess suitability for study
Patients suitable to enter study provided with secure link to study website
Those interested in taking part read information sheet and sign informed consent form online, copy goes to local research team and PI.
Participant will choose pathway. Research team will verify with electronic health records, then inputs into research database
Participants on standard of care pathway
Baseline data collection via link for survey, feeding directly into reserach database
Interview after one week of choice
Month 6 survey via link
Month 12 survey via link
Month 18 survey via link
Month 24 survey via link
Participants on digital pathway
Baseline data collection in app
Month 6 survey in app
Months 12 survey in app
Month 18 survey in app
Month 24 survey in app
The digital pathway participants are recruited for one interview through pathway (6, 12, and 18 months).

Data flows between organisations within the W8Buddy Study

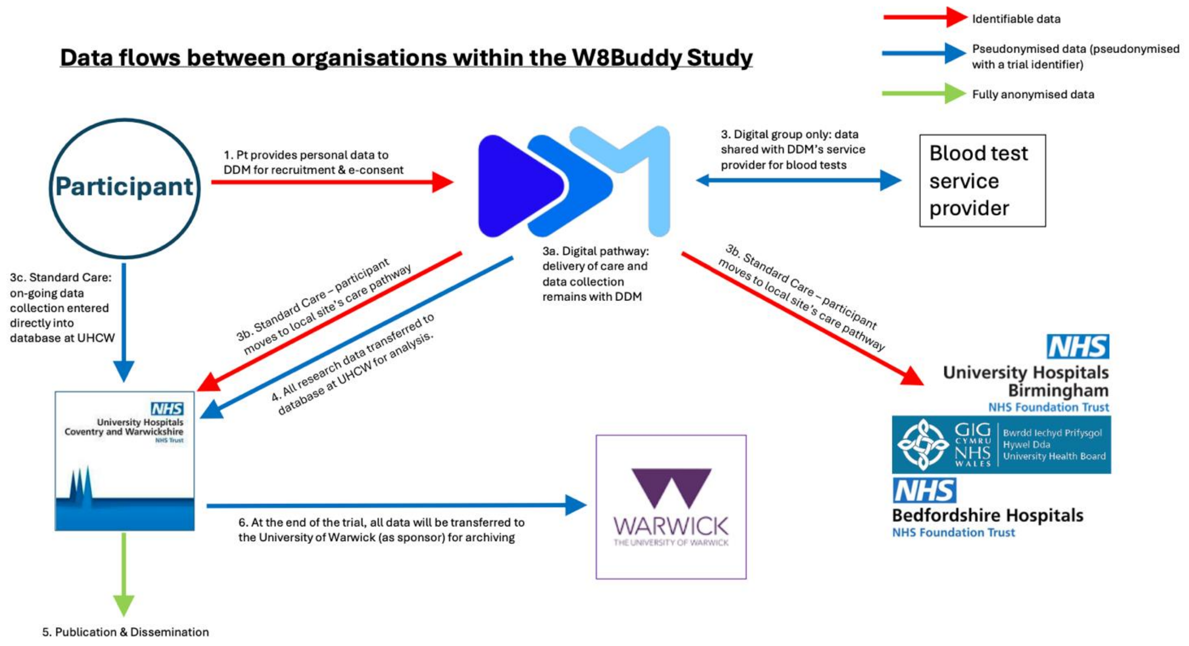

Order of data flows in W8Buddy Study

1. Participant provides personal information to DDM to facilitate online recruitment and e-consent.
2. After giving informed consent, participant chooses pathway:
   a) Digital – participant continues to provide data to DDM for delivery of care and data collection
   b) Standard Care – participant moves to local site's care pathway
   c) Standard Care – on-going data collection for study is entered directly into database at UHCW
3. Digital group only: data shared with DDM's service provider for blood tests.
4. All research data transferred to database at UHCW for analysis.
5. Following completion of the study, the findings will be published and disseminated using fully anonymised data.
6. At the end of the trial, all data will be transferred to the University of Warwick (as sponsor) for archiving

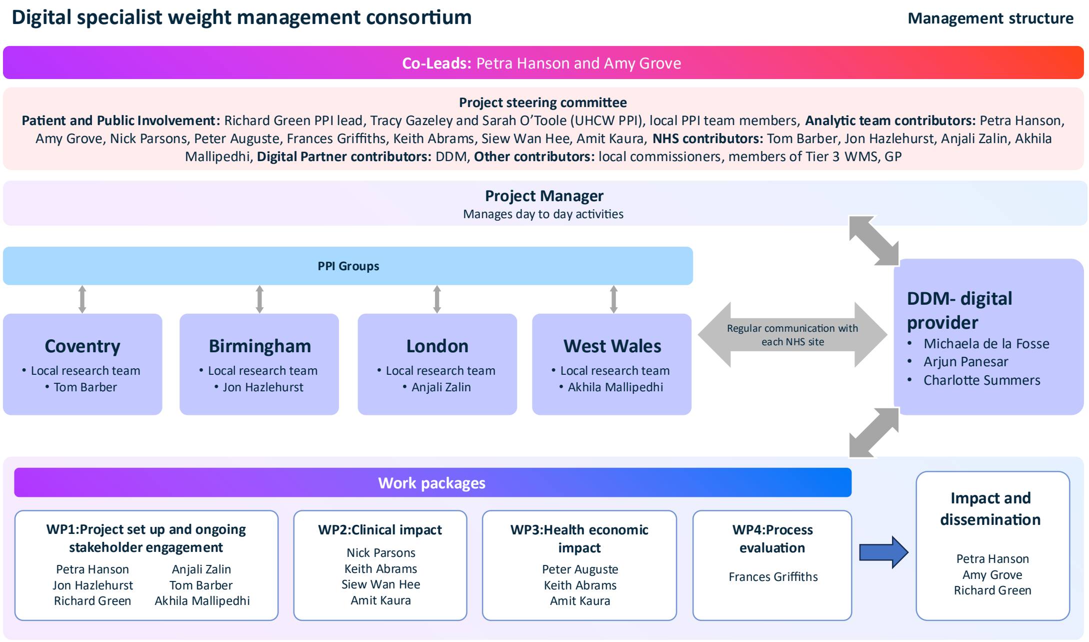
Digital specialist weight management consortium
Management structure
Co-Leads: Petra Hanson and Amy Grove
Project steering committee
Patient and Public Involvement: Richard Green PPI lead, Tracy Gazeley and Sarah O'Toole (UHCW PPI), local PPI team members, Analytic team contributors: Petra Hanson, Amy Grove, Nick Parsons, Peter Auguste, Frances Griffiths, Keith Abrams, Siew Wan Hee, Amit Kaura, NHS contributors: Tom Barber, Jon Hazlehurst, Anjali Zalin, Akhila Mallipedhi, Digital Partner contributors: DDM, Other contributors: local commissioners, members of Tier 3 WMS, GP
Project Manager
Manages day to day activities
PPI Groups
Coventry
• Local research team
• Tom Barber
Birmingham
• Local research team
• Jon Hazlehurst
London
• Local research team
• Anjali Zalin
West Wales
• Local research team
• Akhila Mallipedhi
Regular communication with each NHS site
DDM- digital provider
• Michaela de la Fosse
• Arjun Panesar
• Charlotte Summers
Work packages
WP1:Project set up and ongoing stakeholder engagement
Petra Hanson
Jon Hazlehurst
Richard Green
Anjali Zalin
Tom Barber
Akhila Mallipedhi
WP2:Clinical impact
Nick Parsons
Keith Abrams
Siew Wan Hee
Amit Kaura
WP3:Health economic impact
Peter Auguste
Keith Abrams
Amit Kaura
WP4:Process evaluation
Frances Griffiths
Impact and dissemination
Petra Hanson
Amy Grove
Richard Green

Supplementary table 1. Data management

Information	Description
Data collection tools and source document identification	Study data will be collected on the screening logs, W8Buddy App and validated questionnaires (see Table 1) electronically. Health record data for each site will be accessed by local research team. An online validated, GCP compliant, Electronic Data Capture system will be used to record and store study data, this will be the main Study Database. Individual user log-in access to this database will be granted to only those in the study team that require it for the performance of their role. The W8Buddy App will collect data for participants in the digital arm of the study, then transfer this data in a secure way to the main study database. Only research team and members of the DDM team will have access to the main study database.<br>Source data for standard care procedures will be from hospital medical records. Other outcome measures not collected routinely will either be collected directly from the patient, their clinical care team, or App collaborators and entered directly on to the App/Database.<br>Participants will be able to complete the outcome questionnaires by phone, email or online if they prefer. If done online, they will be emailed a secure, unique link to complete the questionnaires, where their answers will directly input onto the database.<br>The research team at UHCW (see page 1 for details) will maintain oversight of the Study Database during the study to ensure that any missing data is supplied in a timely manner.<br><br>Semi-structured interviews will be audio recorded using teleconferencing software (e.g., Zoom) and transcribed by an external transcription company. The data will be stored on secure University of Warwick computers and accessed only by research team located at Warwick.<br><br>Data entered into the app will be stored in secure Azure cloud.<br>DDM's Data Protection and Information Governance Policy details how we comply to and exceed UK GDPR requirements through:<br>• Consent is requested on referral (website or telephone), paper-based for face-to-face and digitally before using the app. Our privacy policy is available on our website, physically at face-to-face sessions and on in-app/platform. On signup (and signposted in-app), users are informed on how their data will be used, who will access it, their rights and consent/opt-in to

For peer review only

	sharing anonymised data (including with Commissioner and OHID). The app locks users out after several unsuccessful access attempts and logs users out after periods of inactivity. All user access is logged (IP, time, date, browser details) in case of safeguarding or data issues.<br>• Individuals have the right to access their personal data, rectify incorrect data, erase their data and restrict or object to its processing. This is specified in our privacy policy.<br>• We are aware that if we have data breaches, we report within 72 hours of becoming aware of the breach to the Information Commissioners' Office and University's Legal & Compliance Services, in accordance with the University's data protection policies and the ICO as per DDM policies. DDM has a Full Business Continuity Plan which includes a Disaster Recovery Plan tested quarterly.<br>• Service-users' personal data is never transferred outside of the UK. DDM's data servers are hosted by Microsoft Azure in the UK and are fully encrypted-at-rest and in-transit using AES256 cryptography.<br>• Staff are provided an individual account with restricted privileges, dependent on their tasks/role. Any data which is invalid, irrelevant or no longer required (including user removal requests) is securely deleted.<br>• All staff undergo mandatory data/information governance training bi-annually and perform a self-assessment (e.g. passwords, UK GDPR) to identify training needs to provide further formal training.<br>• All information collected, stored and communicated (internally/externally) is carried out in-line with the Data Protection Policy through use of secure digital patient records, encrypted email and private communication software.<br>• All data transfer is done via FTPS (Secure FTP) or encrypted data transferral method.<br>• Paper data and local servers are stored in a locked, CCTV-protected room in DDM Headquarters. All data on the servers is password protected and encrypted using 256-AES (BitLocker on Windows).<br>• One individual via USB/over FTPS takes backups daily. Backups are stored in-the-cloud on a UK-based 256-AES encrypted hard-drive. Paper data is stored in a locked room. Only the DPO has the login details and keys to the data room. All passwords used for data handling

For peer review only

	are 28 characters long and changed weekly. Access is only granted to authorised staff for limited purposes and is time restricted.
Data handling and record keeping	Screening and recruitment logs of all patients approached to take part, and participants enrolled in the study will be held at each site. Upon confirmation of eligibility, participants will be assigned a unique study ID via the study website, which will be used to identify all documents associated with that participant for the duration of the study.<br>It is the responsibility of the PI at each site to ensure that the data that has been submitted is verified and accurate in reflecting the clinical outcome of the participants.<br>This is electronic-based study, with no paper-based collection. Data will be stored in on secure cloud-based databases including the W8Buddy App, with restricted access and regular back-ups in the cloud-based server.<br>Personal data collected during the study will be handled and stored in accordance with the UK GDPR. Handling of personal data will be clearly documented in the participant information sheet and consent obtained.<br>Following all query resolution, the database will be exported for statistical analysis. These procedures along with data entry instructions will be detailed in a Data Management Plan (DMP) produced by the study coordinator and statistician.
Access to data	Direct access will be granted to authorised representatives from the Sponsor, host institution and the regulatory authorities to permit study-related monitoring, audits and inspections - in line with participant consent.<br>Delegated research team members will be responsible for entering data into the database. This may include the site Principal Investigator (PI), research nurses, and study team members based at UHCW, UHB, LTT, and HDUHBT. Access to the data will be restricted to the central study team only. Additionally, all staff granted access to the database will be listed in the delegation log and must hold Good Clinical Practice (GCP) certification.
Archiving	Following the resolution of queries and confirmation of study close-out by the Chief Investigator, all essential documentation will be transferred to a third-party archiving service, which provides suitable fire- and water-resistant facilities. Study files will be archived for a period of 10 years.

For peer review only

	Access to the study documentation will be restricted to named individuals within the study team with express permission from the Chief Investigator.

Supplementary table 2. Safety reporting

Information	Description
Adverse event	An AE is any untoward medical occurrence experienced by a patient while participating in the trial. This includes occurrences which are not necessarily caused by or related to the trial intervention.
Serious adverse event	A SAE is characterised as an untoward occurrence deemed to be related to the intervention under investigation that:<br>1. Results in death within 30 days of the intervention<br>2. Is life-threatening<br>3. Requires hospitalisation or an extension of an existing inpatients' hospitalisation<br>4. Results in persistent or significant disability or incapacity<br>5. Consists of a congenital anomaly or birth defect<br>6. Otherwise considered medically significant by the investigator
Reporting	• The Chief Investigator (or designated representative) will assess all reported SAEs for causality and expectedness.<br>• All intervention related AEs must be documented using the relevant electronic Case Report Form (eCRF), patients will report these to the research team who will then enter this data in the eCRF.<br>• SAEs should be reported for any occurrence from the time informed consent is obtained until study completion.<br>All participants will have contact details to the research team and will be able to inform the research team of any AEs/SAEs occurring between study visits. Participants will be asked to

For peer review only

	contact the research nurse as soon as possible with any potential adverse events. The research nurse will call the patient to discuss the adverse event; this will be escalated as per each standard hospital protocols and will be escalated to each site PI for investigation and recording as required<br>All SAEs must be reported to the study team within 24 hours of the Principal Investigator (or designated representative) becoming aware of the event. This should be done by completing the SAE eCRF and emailing:<br>Email address: W8Buddystudyofficeinbox@uhcw.nhs.uk
Follow up	SAEs must be followed until clinical recovery is complete or the condition stabilises. Outcomes of SAEs should be reported to the study team promptly using the relevant eCRF/CRF. These should be sent to the W8Buddy Study inbox (W8Buddystudyofficeinbox@uhcw.nhs.uk) as soon as the Principal Investigator (or designated representative) is aware of the outcome.<br><br>**EVENTS THAT DO NOT REQUIRE REPORTING AS AN ADVERSE EVENT OR SERIOUS ADVERSE EVENT**<br>This trial recruits' participants with pre-existing long-term conditions (e.g., type 2 diabetes, hypertension), likely requiring medical attention during the trial. Given:<br>• The CE marking of the W8Buddy intervention<br>• Safety not being an outcome of interest<br>A risk-based approach to reporting will apply, in which only AEs deemed related to the W8Buddy intervention will be reported.<br>In addition, the following events will not require reporting as SAEs:<br>• Scheduled hospital visits (e.g., routine outpatient appointments, elective surgeries).<br>• Visits related to pre-existing medical conditions or their progression.<br>• Foreseeable or predictable SAEs attributed to terminal illness or similar conditions.<br>• SAEs related to standard care medications

For peer review only

Supplementary table 3. Study oversight

Information	Description
Roles and responsibilities of the sponsor	The University of Warwick will act as study sponsor. The study will be conducted in accordance with Sponsor's Standard Operating procedures. The University of Warwick has a specialist insurance policy in place which would operate in the event of any participant suffering harm as a result of their involvement in the study Zurich Municipal Insurance. NHS indemnity operates in respect of the clinical treatment that is provided.
Roles and responsibilities of the funder	This study is funded by the National Institute for Health and Care Research Invention for Innovation (NIHR i4i) programme (NIHR208100).
Study manage/co-ordinator	The Trial Co-ordinator / Manager will have responsibility for overseeing day to day coordination of the trial and reporting regularly to the TSC. The Study Manager's responsibilities include, but are not limited to:<br>• Coordinating protocol development, patient and trial management documents<br>• Correspondence with study funder and tracking of progress against agreed milestones<br>• Setting up and maintaining the Trial Master File<br>• Ensuring necessary approvals are in place before the start of the trial at each site<br>• Providing training to trial personnel<br>• Providing data management support; including data input, maintenance of the trial database and raising of queries<br>• Producing trial progress reports and coordinating TSC meetings and minutes<br>• Ensuring data security and quality and ensuring data protection laws are adhered to<br>• Ensuring complete records are in place for audit and monitoring purposes<br>• Ensuring the trial is conducted in accordance with the ICH GCP<br>• Archiving all original trial documents including the data forms in line with UHCW NHS Trust policy.

For peer review only

Site principal investigators	Site Principal Investigator responsibilities include, but are not limited to:<br>• Ensuring that the trial is conducted as set out in the protocol and supporting documents<br>• Delegating trial related responsibilities only to suitably trained and qualified personnel and ensuring that those with delegated responsibilities fully understand and agree to the duties being delegated to them<br>• Ensuring that CVs and evidence of appropriate training for all Site staff are available in the Trial Site File<br>• Ensuring that all delegated duties are captured in the study Delegation Log<br>• Ensuring all Adverse Events are documented and reported promptly to the Trial Manager<br>• Accountability for trial treatments at their site<br>• Ensuring the trial is conducted in accordance with ICH GCP principles.<br>• Allowing access to source data for monitoring, audit and inspection<br>• Ensuring that all source data is complete and provided to the Trial Manager at regular intervals.
Monitoring, audit and inspection	The study will be monitored by the study management group to ensure that the study is being conducted as per protocol, adhering to Research Governance and GCP. Central monitoring activities will be performed such as data quality checks. The approach to, and extent of, monitoring will be specified in a study monitoring plan determined by the risk assessment undertaken prior to the start of the study.<br><br>The recruiting site is obliged to assist the sponsor in monitoring the study. These may include hosting site visits, providing information for remote monitoring, or putting procedures in place to monitor the study internally.<br><br>The study may be monitored by the Warwick University representative of the Sponsor, to ensure that the study is being conducted as per protocol, adhering to Research Governance and GCP. The approach to, and extent of, monitoring will be specified in a study monitoring plan determined by the risk assessment undertaken prior to the start of the study.

For peer review only

Ymchwil Iechyd a Gofal Cymru
Health and Care Research Wales

NHS
Health Research Authority

Dr Petra Hanson
Clinical Lecturer
University of Warwick
Warwick Medical School, University of Warwick,
Coventry
CV4 7AL

Email: approvals@hra.nhs.uk
HCRW.approvals@wales.nhs.uk

29 July 2025

Dear Dr Hanson

HRA and Health and Care Research Wales (HCRW) Approval Letter

**Study title:**	**Long-term health and economic impact of using digital tool Gro Health W8Buddy to support people accessing specialist weight management services**
**IRAS project ID:**	**355370**
**Protocol number:**	**1.0**
**REC reference:**	**25/EM/0147**
**Sponsor**	**Warwick University**

I am pleased to confirm that **HRA and Health and Care Research Wales (HCRW) Approval** has been given for the above referenced study, on the basis described in the application form, protocol, supporting documentation and any clarifications received. You should not expect to receive anything further relating to this application.

Please now work with participating NHS organisations to confirm capacity and capability, in line with the instructions provided in the "Information to support study set up" section towards the end of this letter.

How should I work with participating NHS/HSC organisations in Northern Ireland and Scotland?

HRA and HCRW Approval does not apply to NHS/HSC organisations within Northern Ireland and Scotland.

If you indicated in your IRAS form that you do have participating organisations in either of these devolved administrations, the final document set and the study wide governance report (including this letter) have been sent to the coordinating centre of each participating nation. The relevant national coordinating function/s will contact you as appropriate.

For peer review only

Please see IRAS Help for information on working with NHS/HSC organisations in Northern Ireland and Scotland.

How should I work with participating non-NHS organisations?
HRA and HCRW Approval does not apply to non-NHS organisations. You should work with your non-NHS organisations to obtain local agreement in accordance with their procedures.

What are my notification responsibilities during the study?

The standard conditions document "*After Ethical Review – guidance for sponsors and investigators*", issued with your REC favourable opinion, gives detailed guidance on reporting expectations for studies, including:

- Registration of research
- Notifying amendments
- Notifying the end of the study

The HRA website also provides guidance on these topics, and is updated in the light of changes in reporting expectations or procedures.

Who should I contact for further information?
Please do not hesitate to contact me for assistance with this application. My contact details are below.

Your IRAS project ID is **355370**. Please quote this on all correspondence.

Yours sincerely,
Chris Kitchen

Email: approvals@hra.nhs.uk

Copy to: *Mrs Carole Harris*

For peer review only

List of Documents

The final document set assessed and approved by HRA and HCRW Approval is listed below.

Document	*Version*	*Date*
Contract/Study Agreement template [W8Buddy IRAS 355370 mNCA_July_2022]	v2.3	
Evidence of Sponsor insurance or indemnity (non NHS Sponsors only) [UoW Insurance]	N/A	02 July 2024
GP/consultant information sheets or letters [W8Buddy_GPLetter_v1.0_ 06Jun2025]	v1.0	08 May 2025
Interview schedules or topic guides for participants [W8Buddy_ Topic guides for interviews_v1.0_06Jun2025]	v1.0	06 June 2025
Interview schedules or topic guides for participants [W8Buddy_ Topic guides for HCP interviews_v1.0_12 Jun2025]	1.0	12 June 2025
IRAS Application Form [IRAS_Form_10062025]	N/A	10 June 2025
Letter from funder [Outcome Letter NIHR208100]	N/A	03 September 2024
Letter from sponsor [SOC.11 24-25 W8Buddy Sponsorship Approval Letter]	N/A	06 June 2025
Non-validated questionnaire [W8Buddy_CRSI_v1.0_06Jun2025]	1.0	06 June 2025
Organisation Information Document [Organisation_Information_Document_NonCommercial_v1-8_April_2024+W8Buddy(2)]		01 June 2025
Other [W8Buddy_Websiteconsentprocess_v1.0_06Jun2025]	v1.0	06 June 2025
Other [W8Buddy_Storyboard_v1.0_13Jun2025]	vX.0	13 June 2025
Other [W8Buddy Website Screenshots_v1.0_13Jun2025]	v1.0	13 June 2025
Other [Ethics Response 11Jul2025]	N/A	11 July 2025
Participant consent form [W8Buddy_ConsentForm_HCP_v2.0_ 11Jul2025]	2.0	11 July 2025
Participant consent form [W8Buddy_ConsentForm_v1.0_06Jun2025]	v1.0	06 June 2025
Participant information sheet (PIS) [W8Buddy_CondensedPIS_v1.0_11Jul2025]	v1.0	11 July 2025
Participant information sheet (PIS) [W8Buddy_PIS_HCP_v2.0_11Jul2025]	v2.0	11 July 2025
Participant information sheet (PIS) [W8Buddy_PIS_v2.0_11Jul2025]	v2.0	11 July 2025
Research protocol or project proposal [W8Buddy Protocol_ v1.0_06Jun2025]	v1.0	06 June 2025
Schedule of Events or SoECAT [Funder Export_342299_SoECAT_Gro Health W8Buddy in Specialist Weight Management Services]		
Summary CV for Chief Investigator (CI) [PH CV june 2025]		06 June 2025
Validated questionnaire [PHQ-8_English-UK]		
Validated questionnaire [GAD7_English for the UK]		
Validated questionnaire [Effective_UK (English) EQ-5D-5L Paper Self-Complete v1.2]	v1.2	
Validated questionnaire [BEDS-7_AU1.0_eng-USori]		
Validated questionnaire [Satisfaction With Life Scale]		
Validated questionnaire [Karolinska Sleepiness Scale]		

For peer review only

IRAS project ID	355370

Information to support study set up

The below provides all parties with information to support the arranging and confirming of capacity and capability with participating NHS organisations in England and Wales. This is intended to be an accurate reflection of the study at the time of issue of this letter.

Types of participating NHS organisation	Expectations related to confirmation of capacity and capability	Agreement to be used	Funding arrangements	Oversight expectations	HR Good Practice Resource Pack expectations
Research activities and procedures as per the protocol and other study documents will take place at participating NHS organisations.	Research activities should not commence at participating NHS organisations in England or Wales prior to their formal confirmation of capacity and capability to deliver the study in accordance with the contracting expectations detailed.	An Organisation Information Document has been submitted but the sponsor is intending to use a separate agreement. The sponsor has supplied the unmodified model agreement and intends to use this with participating NHS organisations.	Study funding arrangements are detailed in the Organisation Information Document.	A Principal Investigator should be appointed at participating NHS organisations.	Where an external individual who does not already hold an NHS employment contract will be conducting any of the research activities that will be undertaken at this site type then they would be expected to hold an Honorary Research Contract. This should be issued be on the basis of a Research Passport (if university employed) or an NHS to NHS confirmation of pre-engagement checks letter (if NHS employed). These should confirm Occupational Health Clearance. These should confirm enhanced DBS checks and appropriate barred list checks.<br>External staff holding pre-existing NHS employment contracts, or staff only

For peer review only

					undertaking survey or interview activities should obtain a Letter of Access. This should be issued be on the basis of a Research Passport (if university employed) or an NHS to NHS confirmation of pre-engagement checks letter (if NHS employed). These should confirm Occupational Health Clearance if there is direct contact with participants These should confirm standard DBS checks.

Other information to aid study set-up and delivery

This details any other information that may be helpful to sponsors and participating NHS organisations in England and Wales in study set-up.
The applicant has indicated that they intend to apply for inclusion on the NIHR CRN Portfolio.

For peer review only

Supplementary Table 4

Data collected	When and how data collected from digital (D)/ Standard care (SC) group	Data used for assessment of:
**Weight, height, and BMI**	Month 0,6,12,18,24.<br><br>D: app, dashboard<br><br>SC: survey, clinical notes	Weight loss
**Metabolic markers (HBA1c, lipids)**	Month 0,6,18.<br><br>D: app, dashboard (the same standard of care blood tests will be done remotely instead using clinical phlebotomy partners and samples will be returned for analysis, then discarded as per usual clinical procedures.<br><br>SC: survey, clinical notes (only routine blood test results will be collected in the standard of care pathway, as per usual standard of care for NHS hospitals)	Metabolic impact
**Blood pressure**	Month 0,6,18.<br><br>D: app, dashboard<br><br>SC: survey, clinical notes	Cardiovascular marker
**Waist circumference**	Month 0,6,12,18,24.	Body composition changes

For peer review only

	D: app, dashboard<br><br>SC: survey, clinical notes	
**Health related quality of life (EQ-5D-5L)**	Month 0,6,12,18,24.<br><br>D: app<br><br>SC: survey	Cost-effectiveness and quality of life
**PHQ8, satisfaction with life scale, Karolinska sleepiness scale, GAD7, BEDS-7**	Month 0,6,12,18,24.<br><br>D: app<br><br>SC: survey	Biopsychosocial outcomes
**Clinical endpoints (stroke, myocardial infarction)**	Month 12, 24, 36, 48, 60<br><br>D: app, dashboard<br><br>SC: clinical notes	Major adverse cardiovascular events
**Distance to local NHS facility for participants**	Month 0.<br><br>Research team review of GP notes for D and SC	Health economic calculations
**Time needed to travel to local NHS facility for participants**	Month 0.<br><br>Research team review of GP notes for D and SC, plus	Health economic calculations

For peer review only

	D: app<br><br>SC: survey	
**Date when participants dropped out of study if applicable**	As applicable.<br><br>D: app<br><br>SC: survey	Accurate tracking
**Reason why participant on standard care dropped out of study if applicable**	SC: Contact with research nurse	Why participants drop out of standard care
**Number of consented participants**<br>• **choosing the digital pathway**<br>• **starting on the pathway**<br>• **dropping out**	Month 0-24.<br><br>Reviews of dashboard and research database	Willingness and engagement level of digital pathway<br><br>Sampling frame for participant interviews
**Participant baseline characteristics: comorbidities, sex, age, ethnicity, disabilities, geographical region, socioeconomic status**	Month 0.<br><br>Research team review of GP notes for D and SC, plus<br><br>D: app<br><br>SC: survey	Stratification of interview sample, sensitivity analyses, predictors of cost-effectiveness and engagement with digital pathway
**Comorbidities at follow up (hypertension, obstructive sleep apnoea, osteoarthritis, non-**	Month 6, 12, 18, 24	Assessment of diseases associated with obesity

**alcoholic fatty liver disease, polycystic ovarian syndrome, type 2 diabetes, dyslipidaemia)**	D: app, review of health records<br><br>SC: survey, review of health records	
**Type of concomitant and weight-management medication, dose, and prescription dates, whether NHS or non-NHS prescribed**	Month 0,6,12,18,24<br><br>D: app<br><br>SC: survey, clinical notes	Calculation of cost<br><br>Medication adherence
**Reason for stopping the medicine, including side effects (including physical and psychological effects as per STEP 5 trial. These include nausea, diarrhoea, constipation, vomiting, abdominal pain, dyspepsia, gastroenteritis, decreased appetite, headache, eructation, nasopharyngitis, headache, back pain, upper respiratory tract infection, gallbladder related disorders, cholelithiasis, pancreatitis, cardiovascular disorders, injection site reactions, hypoglycaemia, psychiatric disorders , reaching predefined weight loss goals, lack of adherence to prescribed medicine**	Month 0,6,12,18,24<br><br>D: app, dashboard<br><br>SC: survey, clinical notes	Cost-effectiveness, Biomedical explanation of adherence rates

For peer review only

**Use of and response to digital pathway**	Month 0-24<br><br>D: app<br><br>SC: appointment records and clinical notes<br><br>Information collected on all patients who switch from SC to D and vice versa, even though this is expected to be low	Cost-effectiveness,<br><br>Adherence to digital pathway; variation in adherence by patient characteristics<br><br>User experience and process evaluation
**Adverse events, including only intervention-related adverse effects**	Month 0-24<br><br>D: consultation with clinicians on dashboard, medication tracking, captured at real-time<br><br>SC: clinical notes, surveys at month 6,12,18,24	Cost-effectiveness, understanding the experience of users, creating blueprint for safe incorporation of digital pathway

For peer review only

**Healthcare resource use: Data from providers about the multidisciplinary service, resource use (e.g. rates of referral to bariatric surgery)**<br><br>**Data from participants on number of GP visits, unplanned admissions to A&E**	Month 0,6,12,18,24<br><br>D: app and reports from digital provider<br><br>SC: survey, clinical notes	Cost-effectiveness
**Cost associated with implementing and maintaining digital pathway**	D: Digital provider	Cost-effectiveness
**Cost estimates for specialist WMS**	Month 0,6,12,18,24<br><br>SC: clinical records, survey	Cost-effectiveness
**Implementation, adaptations, and contextual influences**	Observation of pathway delivery team meetings, meeting notes, semi-structured interviews with implementers and clinical staff delivering intervention.<br><br>During initial implementation (month 0) and in months 12 & 18	Implementation process to inform formative feedback<br><br>Manual for future NHS implementation and process evaluation
**Availability of digital pathway and medicine to participant including**<br><br>**staff training and quality assurance**	D: app and related organisational records	Fidelity of delivery of intervention<br><br>Experience of delivery of intervention

For peer review only

	SC: clinical records and related organisational records throughout delivery	Process evaluation
**Brief structured interview with study participants not choosing digital pathway**	Within approx. 1 week of choice<br><br>Remote interview	Why participants do not choose digital pathway (process evaluation)
**Brief structured interview with study participants not adherent to digital pathway and/or medicine**	Sample for participants demonstrating early/late non-adherence or on/off adherence<br><br>Remote interview	Why participants drop out/return to digital pathway/medicine process evaluation)
**Semi-structured interviews with study participants using digital pathway**	Sample stratified by gender and progress through pathway (6, 12, 18 months)<br><br>Remote interview	Understand how user engages with digital pathway, why, perceived impacts and user understanding of mechanism of impact (process evaluation).

Abbreviations: BMI, Body mass index; **EQ-5D-5L**, EuroQol five dimensions and levels; GAD, general anxiety disorder scale; PHQ-8, Patient Health Questionnaire 8, BEDS-7, Binge eating disorder screener

For peer review only
